# Quality of Life and a Surveillant Endocannabinoid System

**DOI:** 10.3389/fnins.2021.747229

**Published:** 2021-10-28

**Authors:** Ricardo Augusto de Melo Reis, Alinny Rosendo Isaac, Hércules Rezende Freitas, Mariana Macedo de Almeida, Patricia Fernanda Schuck, Gustavo Costa Ferreira, Belmira Lara da Silveira Andrade-da-Costa, Isis Hara Trevenzoli

**Affiliations:** ^1^Laboratory of Neurochemistry, Institute of Biophysics Carlos Chagas Filho, Universidade Federal do Rio de Janeiro, Rio de Janeiro, Brazil; ^2^Laboratory of Neuroenergetics and Inborn Errors of Metabolism, Institute of Medical Biochemistry Leopoldo de Meis, Universidade Federal do Rio de Janeiro, Rio de Janeiro, Brazil; ^3^Laboratory of Molecular Endocrinology, Institute of Biophysics Carlos Chagas Filho, Universidade Federal do Rio de Janeiro, Rio de Janeiro, Brazil; ^4^Physiology and Pharmacology Department, Center for Biosciences, Universidade Federal de Pernambuco, Recife, Brazil

**Keywords:** THC – tetrahydrocannabinol, cannabidiol, diet, exercise, meditation, anandamide, BDNF, metabolic programming

## Abstract

The endocannabinoid system (ECS) is an important brain modulatory network. ECS regulates brain homeostasis throughout development, from progenitor fate decision to neuro- and gliogenesis, synaptogenesis, brain plasticity and circuit repair, up to learning, memory, fear, protection, and death. It is a major player in the hypothalamic-peripheral system-adipose tissue in the regulation of food intake, energy storage, nutritional status, and adipose tissue mass, consequently affecting obesity. Loss of ECS control might affect mood disorders (anxiety, hyperactivity, psychosis, and depression), lead to drug abuse, and impact neurodegenerative (Alzheimer’s, Parkinson, Huntington, Multiple, and Amyotrophic Lateral Sclerosis) and neurodevelopmental (autism spectrum) disorders. Practice of regular physical and/or mind-body mindfulness and meditative activities have been shown to modulate endocannabinoid (eCB) levels, in addition to other players as brain-derived neurotrophic factor (BDNF). ECS is involved in pain, inflammation, metabolic and cardiovascular dysfunctions, general immune responses (asthma, allergy, and arthritis) and tumor expansion, both/either in the brain and/or in the periphery. The reason for such a vast impact is the fact that arachidonic acid, a precursor of eCBs, is present in every membrane cell of the body and on demand eCBs synthesis is regulated by electrical activity and calcium shifts. Novel lipid (lipoxins and resolvins) or peptide (hemopressin) players of the ECS also operate as regulators of physiological allostasis. Indeed, the presence of cannabinoid receptors in intracellular organelles as mitochondria or lysosomes, or in nuclear targets as PPARγ might impact energy consumption, metabolism and cell death. To live a better life implies in a vigilant ECS, through healthy diet selection (based on a balanced omega-3 and -6 polyunsaturated fatty acids), weekly exercises and meditation therapy, all of which regulating eCBs levels, surrounded by a constructive social network. Cannabidiol, a diet supplement has been a major player with anti-inflammatory, anxiolytic, antidepressant, and antioxidant activities. Cognitive challenges and emotional intelligence might strengthen the ECS, which is built on a variety of synapses that modify human behavior. As therapeutically concerned, the ECS is essential for maintaining homeostasis and cannabinoids are promising tools to control innumerous targets.

## Introduction

The endocannabinoid system (ECS) controls a widespread and abundant metabolic network. It impacts on many symptoms experienced by adults or children during the COVID-19 pandemics, including chronic pain, lack of exercise, poor diet and gain of weight, mood disorders, as depression, anxiety (Micale et al., 2013, 2015; Kucerova et al., 2014) or increased stress due to lockdown, social distancing, and job loss, as well as due to exhausting work shifts for intensive care staff (Rogers et al., 2020; Bennett et al., 2021). The ECS has been studied systematically since the elucidation of the structure of tetrahydrocannabinol (THC) from Cannabis (Mechoulam and Gaoni, 1965), and later recognized as a physiological circuit breaker with the discovery of membrane receptors, enzymes, and endocannabinoid-like mediators (De Petrocellis et al., 2004; Katona and Freund, 2008). Alternatively, more people became interested in meditation and mindfulness healings suggesting that alternative therapies might improve measures of anxiety, depression and pain scores, and possibly on the mechanisms of plastic brain changes on people with a long-term traditional meditation practice (Behan, 2020). As the receptors were initially cloned and mapped in the 1990s, it became clear that two major branches emerged from the ECS: one highly enriched in the brain (Herkenham et al., 1991) and its peripheral nerves and the other in the immune system (Facci et al., 1995). Today, a multitude of direct and indirect intra- and extracellular targets in almost all physiological systems constitutes the endocannabinoidome, an ensemble of eCBs and their receptors and metabolic enzymes (Di Marzo and Piscitelli, 2015) to form a multi-facet therapeutic platform (Kaur et al., 2016). This is the core of the recent cannabinoid medicine field that claims to improve several maladies as chronic pain and spasticity (Whiting et al., 2015), but that still raises many concerns due to controversies of the matter and the adverse effects shown by phytocannabinoids.

## What Does the Endocannabinoid System Consist Of?

**FIGURE 1:**
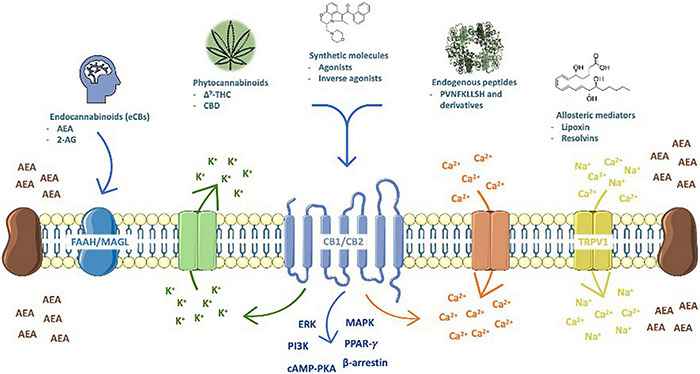
The endocannabinoid system (ECS) is composed of lipid endocannabinoids (eCBs), allosteric (lipoxins and resolvins) and peptide (hemopressin derivatives) mediators, their receptors (the cannabinoid type 1 (CB1) and type 2 (CB2), which are activated by phyto- (THC, CBD, and possibly many others) and synthetic cannabinoids (represented by WIN55,212-2, a mixed agonist), metabolic enzymes (FAAH and MAGL, and others) and membrane transporters. Upon activation, CB1 and CB2 signal through fast (Ca^2+^ and K^+^ currents) and/or slow pathways, as cyclic AMP-protein kinase A (cAMP-PKA), ıextracellular signal-regulated (ERK), beta-arrestin, mitogen-activated protein kinase (MAPK) and PI3K; in addition, gene transcription is also turned on by nuclear receptors (PPARγ and others).

The ECS is composed of lipid endocannabinoids (eCBs) and peptide (hemopressin derivatives) mediators, their receptors [the most prominent are the type 1 (CB1) and type 2 (CB2) cannabinoid receptors], metabolic enzymes and membrane transporters (Figure 1). CB1 and CB2 are G-protein coupled receptors (Mallipeddi et al., 2017) highly concentrated on major brain areas (Herkenham et al., 1990) such as neurogenic niches (Xapelli et al., 2013), that upon activation signal through fast (Ca^2+^ and K^+^ currents) (Kano et al., 2009) and/or slow pathways, as cyclic AMP-protein kinase A (cAMP-PKA), extracellular signal-regulated (ERK), beta-arrestin, mitogen-activated protein kinase (MAPK) and PI3K (Priestley et al., 2017; Haspula and Clark, 2020); gene transcription is also turned on by nuclear receptors (of the PPAR family), which increases plasticity (Pistis and O’Sullivan, 2017), and are targeted by the ECS.

Lipid eCBs are endogenously generated from membrane phospholipids that contain arachidonic acid (AA) (Freitas et al., 2018) to derive the N-arachidonoylethanolamide (anandamide or AEA) and 2-arachidonoylglycerol (2-AG). AEA is a partial agonist of both CB1 and CB2 receptors with a higher relative intrinsic efficacy and affinity for CB1 receptors. Alternatively, 2-AG shows a higher potency compared to AEA as a cannabinoid receptor (CB) agonist, binding with the same affinity to both receptors (Pertwee, 2010). Other “weak” eCB [2-arachidonoyl glyceryl ether, O-arachidonoylethanolamide (OEA) and derivatives of long-chain N-acyl-amides, including N-acyl-taurines, N-acyl-serotonins, N-acyl-dopamines, and fatty acid primary amides] might also contribute with different responses, depending on the tissue or the metabolic condition (Ramer et al., 2019; Cristino et al., 2020). Indeed, palmitoyl-ethanolamide (PEA), one of these eCB, when given as a dietetic powder, rescues learning and memory impairments in a triple transgenic mouse model of Alzheimer’s disease (AD) by exerting anti-inflammatory and neuroprotective effects (Scuderi et al., 2018).

Plant-derived phytocannabinoids, i.e., THC and cannabidiol (CBD), the two most acknowledged metabolites out of hundreds of molecules present in *Cannabis sativa* L., are highly studied due to their general effects on the brain. Both THC and CBD were isolated in the mid-1960s and display many important effects. THC, for instance, is psychoactive and known to induce relaxation, euphoria, and memory impairment (Mechoulam and Parker, 2013). However, the misuse of Cannabis might affect the function of the brain and/or induce psychosis at critical developmental stages as pregnancy or adolescence (Volkow et al., 2014; Alpár et al., 2016). Indeed, cannabinoid exposure during prenatal/perinatal and adolescent periods might alter synaptic plasticity in neurodevelopmental processes, in which the ECS plays an essential role (Bara et al., 2021). On the other hand, CBD is a potent anti-inflammatory, anxiolytic, antidepressant, antipsychotic, anticonvulsant, anti-nausea, antioxidant, antiarthritic, and antineoplastic agent (Ligresti et al., 2016). Although non-psychotomimetic, CBD presents promising therapeutic effects on the brain, known to reduce brain damage associated with neurodegenerative and/or ischemic conditions. It also has positive effects on attenuating psychotic-, anxiety-, and depressive-like behaviors (Campos et al., 2016). Indeed, CBD was able to prevent the development of molecular and behavioral schizophrenia (SCZ)-like alterations in neurodevelopmental animal models, without inducing side effects (Stark et al., 2019, 2020). This latter paper shows for the first time that CBD seems both to normalize the D3 receptor expression in gestational methylazoxymethanol acetate (MAM) model of SCZ and to bind preferentially to dopamine D3 receptors, as novel potential mechanism of action. In addition, CBD treatment may normalize perinatal THC-exposed male rats-induced psychopathology by modulating the altered dopaminergic activity and transcriptional regulation of the genes encoding for the cannabinoid CB1 receptor (Cnr1) and the dopamine D2 receptor (Drd2) (Di Bartolomeo et al., 2021). Cannabinoids are not the only compounds that can influence eCB tone; aptly called *cannabimimetic*, there are several foods, such as dietary and omega (n-3 and n-6) fatty acids as important intermediaries for energy metabolism, influencing feeding behavior, neural plasticity, physical activity (PA), and cognition during aging and activities that can signal through our ECS for optimal health (Freitas et al., 2017).

In addition to the lipid agents, a new class of endogenous peptides derived from hemopressin (HP), PVNFKLLSH, a fragment derived of the α-chain of hemoglobin has been recently investigated. HP acts as an inverse agonist of the CB1 receptor, consequently regulating the antinociceptive activity (Toniolo et al., 2014), food intake (Dodd et al., 2010), and inducing oligodendrocyte differentiation (Xapelli et al., 2014) in the subventricular region of the newborn mice (Xapelli et al., 2013; reviewed in Heimann et al., 2020a). HP extended forms (RVD- and VD-HP) are agonists of CB1 receptors (Gomes et al., 2009). An HP fragment NFKF was recently shown to promote analgesia, delay seizure induced by pilocarpine, and prevent neurodegeneration in an experimental model of autoimmune encephalomyelitis (Heimann et al., 2020b). These effects have caught the attention of pharmaceutical companies. In addition, the recent wave of Cannabis legalization in several western countries and the surge of the so-called marijuana stocks have attracted investors and are worth billions of dollars transforming a once abused illicit drug field into a promising area for investors.

## Endocannabinoid System Fine-Tune Physiology Regulation

The ECS is an underlying system contributing to homeostasis in many of our body’s physiological and cognitive processes (Alteba et al., 2016), including but not limited to mood, memory, appetite, energy, pain, cardiovascular and respiratory function, and neuro-immune modulation. Cannabinoid receptors are highly expressed in the brain and in virtually all peripheral tissues regulating physiological functions directly or indirectly through the autonomic nervous system. Regarding the expression of CB receptors, it is well known that the CB1 receptors are present at very high levels on inhibitory (GABAergic interneurons) and at a lesser extent on excitatory (glutamatergic) terminals (Marsicano and Lutz, 1999), as well as on neurons expressing dopamine D1 receptors, playing a specific role in the repertoire of different emotional behaviors including social and cognitive activity, which are affected in psychiatric disorders (Terzian et al., 2011, 2014; Llorente-Berzal et al., 2015; Micale et al., 2017). Cannabinoid signaling is found in all players of the quadripartite synapse, formed by pre- and postsynaptic neurons, astrocytes, and microglia, in a highly interacting device, adjusting many functions in the Central nervous system (CNS; Ligresti et al., 2016). CB1 and CB2 receptors are located both on neurons and glial cells and are considered the main circuit breakers, as activation of pre-synaptic CB1 (and possibly CB2) receptors inhibits the release of the major neurotransmitters glutamate (excitatory) and GABA (inhibitory) (Katona and Freund, 2008). Also, ATP, the major signal secreted by astrocytes (Rodrigues et al., 2015) and microglia (Ferrari et al., 1997) signals through P2Y and P2X purinergic receptors which are modulated by ECs through hemichannels (Labra et al., 2018).

In addition to the canonical pathway mediated by CB1 and/or CB2 receptors, additional targets are also considered as GPR18 and GPR55 (Irving et al., 2017), the transient receptor potential vanilloid 1, TRPV1 (Muller et al., 2018) or heterodimers for many different receptors (dopamine, serotonin, and hormones) (Wellman and Abizaid, 2015), which might increase the complexity of spatial-temporal responses. Indeed, experimental evidence points to the fine-tuning of membrane receptor-interacting proteins, as the cannabinoid receptor-interacting protein 1a (CRIP1a) (Oliver et al., 2020), increasing the complexity in terms of cellular localization and functions, ranging from food intake regulation and energy balance to mechanisms of brain plasticity and cancer. Signaling devices are expressed in different cell types, which could act as frameworks to modulate G protein-mediated signaling (Ritter and Hall, 2009) and explain several of the conflicting effects exerted by eCBs, phyto-, and synthetic cannabinoids.

N-arachidonoylethanolamide levels are degraded by the fatty acid amide hydrolase (FAAH) enzyme, a serine hydrolase found in cell bodies and dendrites of postsynaptic neurons in major areas of the brain (Egertová et al., 2003; Otrubova et al., 2011). eCBs, are known as retrograde messengers [most of the synthetic enzymes are centered post-synaptically, operating on demand, activated by electrical activity, and/or calcium shifts (Regehr et al., 2009)]. FAAH inhibitors have attracted interest from the pharmaceutical industry as they prolong the accurately regulated pro-homeostatic actions of AEA (Petrosino and Di Marzo, 2010), inducing, for instance, powerful analgesia (Tripathi, 2020). A recent case emerged from a Scottish native that faced a lifelong record of adversities resulting in painful events (hand surgery due to arthritis, joint degeneration, cuts, and burns that healed briefly), with no complaint of discomfort. That lead to the identification of a microdeletion in FAAH conferring reduced expression and activity resulting in high AEA concentrations and pain insensitivity (Habib et al., 2019). As chronic pain is one of the major topics of the XXI century, this might open a new avenue for treatment by targeting drugs or by the use of medical Cannabis (Vučković et al., 2018).

## Healthy Diet, Supplements, and Natural Products

Balanced diets in macro and micronutrients are fundamental to the correct CNS development and maturation, for allowing structural changes and specific metabolic signals in homeostatic or pathological conditions (Cusick and Georgieff, 2016). ECS represents a link between dietary lipids and synaptic activity, and it is involved in several mechanisms related to the development and neuroplasticity (Lafourcade et al., 2011; Freitas et al., 2018; Andrade-da-Costa et al., 2019). An increasing number of studies have suggested its participation in antioxidant, anti-inflammatory, and cytoprotective mechanisms, indicating the potential therapeutic of this system in some neurological diseases (Velayudhan et al., 2014), as well as in conditions of systemic inflammation and obesity (Simopoulos, 2016). Experimental studies of selected diets such as the Mediterranean, which consists of unsaturated lipids from fish, olive oil, fruits, vegetables, whole grains, and legumes/nuts, suggest better physiological parameters decreasing the burden, or avoiding the outcome of cardiovascular disease, stroke, depression, several types of cancer, diabetes, obesity, and dementia (Widmer et al., 2015; Geisler, 2016; Assmann et al., 2018; Radd-Vagenas et al., 2018). The Mediterranean diet impacts on the plasma concentrations of eCBs, altering N-acylethanolamines, and their specific ratios in people with lifestyle risk factors for metabolic diseases (Tagliamonte et al., 2021), causing changes in the gut microbiome and metabolome (Meslier et al., 2020). This is important due to conditions faced by hundreds of millions around the globe exposed to dietary inequalities. In Brazil, for example, a change has been noticed from an undernutrition status in impoverished areas from the Pernambuco State in the 1970–1980s and modeled as the Regional Basic Diet (RBD) to western-type high-fat foods nowadays (de Aquino et al., 2019; Jannuzzi et al., 2021). This nutritional transition from chronic consumption of hypoproteic (RBD) or high-fat diets may have consequences to the general health of the population (de Aquino et al., 2019).

**FIGURE 2:**
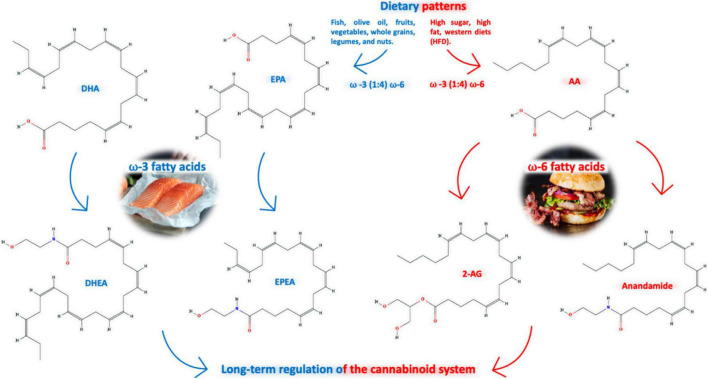
Dietary patterns, as the Mediterranean (n-3 series enriched) are linked to better physiological parameters decreasing the outcome of several types of diseases, which consists of unsaturated lipids from fish (and olive oil, fruits, vegetables, whole grains and legumes/nuts), impacting on the levels of eCBs; they are influenced by the content of n-3 series derived from alpha-linolenic acid (ALA, 18:3n-3) and n-6 series (enriched in Western diet) derived from linoleic acid (LA, 18:2n-6), essential polyunsaturated fatty acids (PUFA); both AEA and 2-AG are derived from AA of the n-6 family, while N-docosahexaenoyl-ethanolamine (DHEA) and N-eicosapentaenoyl-ethanolamine (EPEA), are derived from the n-3 PUFAs docosahexaenoic acid (DHA) and eicosapentanoic acid (EPA).

The levels of eCBs and their activity at CBs are influenced by the content of n-6 series derived from linoleic acid (LA, 18:2n-6), and n-3 series derived from alpha-linolenic acid (ALA, 18:3n-3), essential polyunsaturated fatty acids (PUFAs) in the diet (Freitas et al., 2018); in addition, activity of biosynthetic and catabolic enzymes of the ECS and the way they exert important roles impact the regulation of appetite and metabolism (Banni and Di Marzo, 2010). Both AEA and 2-AG are derived from AA of the n-6 family (Tsuyama et al., 2009), while N-docosahexaenoyl-ethanolamine (DHEA), N-eicosapentaenoyl-ethanolamine (EPEA), 2-acylglycerols, 2-docosahexaenoylglycerol (2-DHG), and 2-eicosapentaenoylglycerol (EPG) are derived from the n-3 PUFAs docosahexaenoic acid (DHA) and eicosapentanoic acid (EPA) (Figure 2; Bisogno and Maccarrone, 2014). Alpha-linolenoylethanolamide (ALEA) is another eCB produced from the n-3 ALA, which is detected in human plasma, and it is responsive to dietary supplementation (Jones et al., 2014).

Another class of n-3 PUFA-derived lipid metabolites present in the brain and peripheral organs comes from the crosstalk between eCB and cytochrome P450 (CYP) epoxygenase metabolic pathways. The n-3 eCB epoxides are originated from DHA and EPA to form epoxyeicosatetraenoic acid-ethanolamide (EEQ-EA) and epoxydocosapentaenoic acid-ethanolamide (EDP-EA), respectively. These n-3 endocannabinoid epoxides have anti-inflammatory and vasodilatory properties and can modulate platelet aggregation (McDougle et al., 2017).

Considering that diet is the largest source of substrate for the biosynthesis of eCBs in mammals, dietary changes in the n-6/n-3 ratio can directly modulate their levels in tissues and, consequently, their biological functions (Bisogno and Maccarrone, 2014). Evidence has shown that deficiency in n-3 during pregnancy and lactation is capable of negatively altering functions mediated by the ECS in the offspring. Young mice, submitted to a maternal n-3 deficient diet, showed an inhibition of long-term depression (LTD) mediated by n-6 eCB and a reduced sensitivity of the CB1 receptor in the prefrontal cortex and nucleus accumbens (Lafourcade et al., 2011); in addition, changes in long-term potentiation (LTP) and LTD in the hippocampus (Thomazeau et al., 2017) and in the mitogen activated protein kinases (MAPK) signaling pathway after activation with CB1/CB2 agonists were also detected in the prefrontal cortex and hypothalamus (Larrieu et al., 2012). These studies suggest that such changes in synaptic plasticity mechanisms may be related to the increase in anxiety- and depression-like behaviors observed in n-3 deprived animals. Thus, n-3 PUFA and the ECS modulate several functions through neurodevelopment including synaptic plasticity mechanisms. Our group has recently shown that changes in maternal dietary DHA levels may impact differently on the ECS and molecular markers highlighted by increased synaptophysin levels in the neonate brain, CB1/2 receptor levels in dams and neonates’ brain, glial fibrillary acidic protein (GFAP) levels, and protein kinase A (PKA) phosphorylation in the cortex and ERK phosphorylation in the hippocampus of the progeny (Isaac et al., 2021).

*In vitro* studies with long chain polyunsaturated fatty acids (LC-PUFAs) DHA or EPA were also able to modulate the ECS. DHA supplementation in hippocampal neuron cultures promoted increased mRNA and protein levels of CB1 and TRPV1 receptors, in a dose-dependent manner (Pan et al., 2011). Indeed, it was reported that addition of DHA and EPA promotes increased levels of 2-AG in neural stem cell cultures. The presence of EPA also increased cell proliferation and activation of the p38-MAPK pathway, showing a relationship between proliferation, eCBs and n-3 derivatives (Dyall et al., 2016).

Endocannabinoid system has systemic effects in the regulation of food acquisition, energy sensing and metabolism (Banni and Di Marzo, 2010). Overactivation of the 2-AG and AEA (derived from AA) stimulate neural mechanisms involved in the appetite and can favor food-related disorders such as obesity and inflammation (D’Angelo et al., 2020). Therefore, a competition for shared biosynthetic pathways between n-3 and n-6 fatty acids and the opposite systemic effects of these lipids might modulate the final action of eCBs in a range of tissues. n-3 derived eCBs from DHA or EPA have anti-inflammatory properties and their chronic supplementation in humans or animals reduces 2-AG and AEA levels (Batetta et al., 2009; Banni and Di Marzo, 2010; Berge et al., 2013). As a consequence, a lower body mass index (Thorsdottir et al., 2007) and a preventive effect on the development of obesity have been reported (Rossmeisl et al., 2012; Simopoulos, 2020). The recommended n-6/n-3 ratio for optimal eCB production is 4:1 (Freitas et al., 2018). Nevertheless, the current Western diet adopted in many countries with high amounts of vegetable oils enriched with alpha-linoleic fatty acid usually lends itself to ratios closer or higher than 16:1. Thus, dietary supplementation with popular food sources of n-3 such as fish (mackerel, salmon, seabass, and sardines), seaweed, edamame, hemp, chia, and flax seeds, are suggested as a part of a balanced lifestyle. Additionally, the anti-inflammatory, anti-cancer, and hypotriglyceridemic effects of these fatty acids and derived n-3 endocannabinoids are also involved in reproduction control and in the stress response which reinforce actions which are co-preventative and co-therapeutic in the management of several diseases (D’Angelo et al., 2020).

Medicinal plants are part of diet since early stages of human civilization. Thus, evidence-based alternative medicine of the cannabimimetic activity of many natural products, their wide availability and low side effects stimulate future studies for their inclusion as a part of a balanced dietary lifestyle. This could be especially relevant for targeting endocannabinoid dysregulation. Complementary to dietary interventions using balanced levels of essential fatty acids, natural bioactive compounds obtained in several plants can act as phytocannabinoids, showing affinity, adequate potency, and efficacy on CB receptors, and some of them might also act on metabolizing enzymes, thus modulating the ECS activity (Gertsch et al., 2010). Compared to synthetically derived cannabinoids, naturally derived molecules induce few adverse effects and their use as promising and emerging therapeutic alternative has been investigated for treatment of several metabolic or neurodegenerative diseases (Sharma et al., 2015).

**FIGURE 3:**
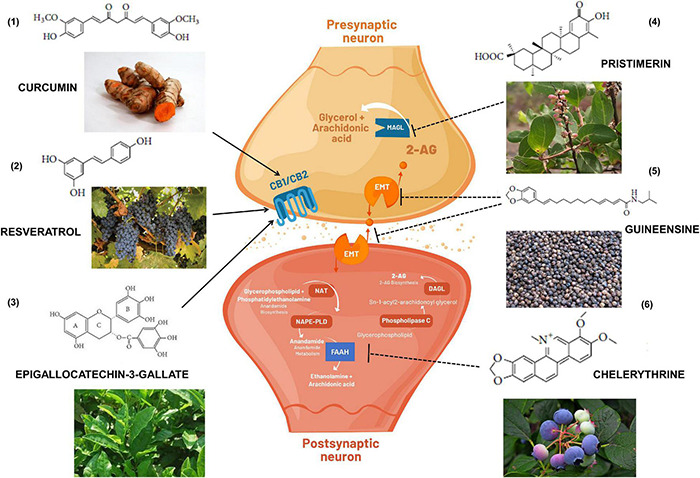
Cannabimimetic activity of bioactive substances obtained in foods and medicinal plants that if included in the diet could indirectly exert immunomodulatory and beneficial effects to the health. (1) turmeric root; (2) grapes; (3) green tea; (4) *Maytenus senegalensis* (Lam.) fruit and roots; (5) black peppers; and (6) blueberries. Arrows indicate activation of CB1 and/or CB2 receptors. Dashed arrows indicate inhibition of endocannabinoid transporters (EMT) or enzymatic metabolism *via* FAAH (Fatty acid amide hydrolase) or MAGL (Monoacylglycerol lipase). DAGL: Diacylglycerol lipase; NAPE-PLD: N-acyl phosphatidylethanolamine phospholipase D; NAT: N-acetyltransferase; 2-AG: 2-Arachidonoylglycerol.

The diverse chemical classes of these phytocannabinoids ligands (Figure 3) include alkaloids, terpenes, terpenoids, and polyphenols (Sharma et al., 2015). The sesquiterpene β-caryophyllene, for example, can be found in essential oil of cloves, oregano, cinnamon, black pepper, hemp, rosemary, and hops (Gertsch et al., 2008). It is commonly used in food, cosmetics, and fragrances as flavoring agent and exerts potent cannabimimetic anti-inflammatory actions including CB2-dependent therapeutic effects in cerebral ischemia (Choi et al., 2013), insulin resistance (Suijun et al., 2014), glutamate neurotoxicity (Assis et al., 2014), renal injury (Horváth et al., 2012), anxiety and depression (Bahi et al., 2014), neuropathic pain (Klauke et al., 2014), and AD (Cheng Y. et al., 2014).

Polyphenol compounds found in the leaves of teas, in several fruits and legumes, such as catechins, exhibit binding properties with CB1 and CB2 receptors in a dose-dependent manner (Korte et al., 2010). In addition, curcumin, another polyphenol that inhibits tumor growth by increasing ROS levels and the antioxidant glutathione (GSH) (Larasati et al., 2018) has been linked to a cannabinoid activity in multiple physiological systems, such as alternative treatments for inflammatory bowel disease, other digestive diseases or liver fibrosis (Zhang et al., 2013; Quezada and Cross, 2019), alone or in the presence of hemopressin (El Swefy et al., 2016).

Resveratrol is a compound present in fruits and plants with beneficial effects for the health, whose pharmacological properties have been widely investigated. Resveratrol exhibits peripheral antinociception through opioid (μOR) and cannabinoid (CB1) receptor activation in hyperalgesia induced by carrageenan in the paw withdrawal method (Oliveira et al., 2019). The extract of several medicinal plants have been analyzed regarding their ability to bind on CBs (Sharma et al., 2015). Cannabinoid-dependent beneficial effects of these extracts have been indicated on neuropathic pain, immunomodulation, inflammation, lung injury, obesity, colon cancer, osteoporosis, and diabetes (Palu et al., 2008; Cotrim et al., 2012; Styrczewska et al., 2012; Velusami et al., 2013; Liu et al., 2014; Lim et al., 2015).

## Exercise

Routine PA has the potential to improve several physiological parameters at different organs, leading to modifications in metabolic, cardiovascular, and immune routes. It is common sense that PA provides a sharp memory, better cognition, and helps with sleep cycle regulation. Indeed, PA has been shown to revert some of the deleterious effects of a sedentary lifestyle, delay brain aging, and neurodegenerative pathologies such as AD, diabetes, and multiple sclerosis (Di Liegro et al., 2019). Aerobic fitness (essential for endurance activity) and aerobic capacity (measured as maximal oxygen consumption during exercise, VO_2_ max) results in major adaptations of the cardiorespiratory and neuromuscular systems that increase the distribution of oxygen to the mitochondria and enable a tighter regulation of muscle metabolism (Jones and Carter, 2000), normalizing blood pressure with less risk of stroke, preventing, and treating cardiometabolic diseases like obesity and type 2 diabetes and cardiovascular diseases. Also, it prevents other chronic disorders (cancer, hypertension, obesity, depression, and osteoporosis) and premature death (Warburton et al., 2006). It is a common belief that most of the reward induced by acute or chronic exercises (reward, nociception, mood behavior, anxiety, and performance) are in part related to the release of endorphins and interactions with multiple opioid (mu, kappa, and delta) receptors and/or sensitivity shifts on the receptors (Arida et al., 2015).

**FIGURE 4:**
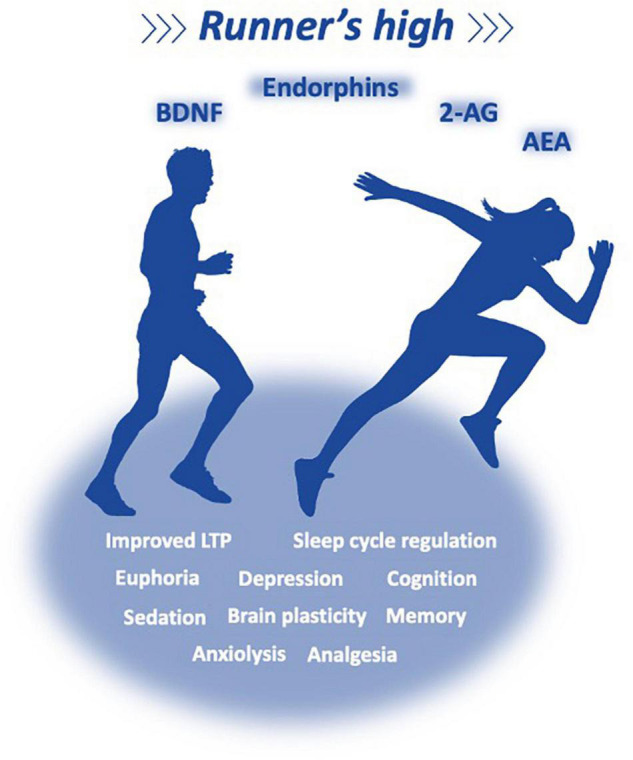
Exercise reverts some of the collateral effects of a sedentary lifestyle, and has the potential to improve metabolic, cardiovascular, and immune parameters, providing a better memory, cognition, and sleep cycle regulation, delaying brain aging and chronic and neurodegenerative pathologies. It is a common belief that most of the reward induced by acute or chronic exercises (reward, nociception, mood behavior, anxiety, and performance) are related to the release of endorphins and eCBs, which interact with multiple opioid (mu, kappa, and delta) and cannabinoid receptors; Irrefutable evidence demonstrate that the ECS is a major player in systemic energy metabolism, inflammation, appetite control, and pleasure (acute anxiolysis, analgesia, antidepressant effects, sedation, and euphoria) of the so-called runner’s high.

However, in the last two decades, irrefutable evidence demonstrated that the ECS is also a major player in systemic energy metabolism, inflammation, appetite control, and pleasure (acute anxiolysis, analgesia, antidepressant effects, sedation, and euphoria) of the so-called runner’s high (Fuss et al., 2015). In terms of mechanisms, voluntary exercise controls hippocampal plasticity independently to the ECS. Voluntary exercise increased the proliferation of progenitor cells, as evidenced by the increase in the number of Ki-67 positive cells in the granule cell layer of the dentate gyrus (DG) in the hippocampus. However, this effect was abrogated by concurrent treatment with AM251, a CB1 antagonist, indicating that the increase in endocannabinoid signaling in the hippocampus is required for the exercise-induced increase in cell proliferation. These data demonstrate that the ECS in the hippocampus is sensitive to environmental changes and suggest that it is a mediator of exercise-induced plasticity (Hill et al., 2010). Rats submitted to forced exercise (treadmill-running training) show an improved expression of LTP in the DG and enhanced object recognition learning (O’Callaghan et al., 2007). Functional changes are linked with an increase in the expression of brain-derived neurotrophic factor (BDNF), a key player for exercise-induced brain plasticity (O’Callaghan et al., 2007; Soya et al., 2007; Wrann et al., 2013). As higher BDNF levels and ECS activation could have positive effects on depression, an investigation was made on intense exercise in 11 healthy trained male cyclists. The plasma levels of AEA and BDNF were increased, whereas 2-AG concentrations remained stable during exercise and the 15 min recovery (Heyman et al., 2012). This indicates that an increase in AEA during exercise might be one of the factors involved in the exercise-induced increase in peripheral BDNF levels and that AEA high levels during recovery might delay the return of BDNF to basal levels (Figure 4). Indeed, recent data described that aerobic exercise induced increases in peripheral AEA and BDNF which play a role in enhancing consolidation of fear extinction learning (Crombie et al., 2021). Therefore, an increase in the peripheral levels of AEA and BDNF might be a mechanism underlying neuroplasticity and antidepressant effects of exercise (Heyman et al., 2012) and might be a promising candidate to reduced threat expectancies following reinstatement among women with posttraumatic stress disorder (Crombie et al., 2021).

Curiously, eCBs are elevated not only with acute exercise but also with obesity. Transcriptomic response of skeletal muscle to acute and chronic aerobic and resistance exercise confirms the expression of major cannabinoid players in the synthesis and breakdown of eCBs, possibly involved with the anti-inflammatory effect of exercise (Schonke et al., 2020). Recent multi-omic studies (metabolome, lipidome, immunome, proteome, and transcriptome) performed on plasma and peripheral blood mononuclear cells from volunteers subjected to acute PA (before and after a controlled session of symptom-limited exercise) revealed thousands of changes on analytes and a coordinated strategy of procedures involving energy metabolism, oxidative stress, inflammation, tissue repair, and growth factor response, as well as regulatory pathways (Contrepois et al., 2020). An increase in eCBs levels is correlated with metabolic disorders as higher lipogenesis is found in the liver and adipocytes, and lower insulin sensitivity in peripheral tissues (Mazier et al., 2015). Finally, Cannabis use has increased in the recent past, in large part due to decriminalization. Even though the ECS is central to the benefits induced by PA, it is currently unknown if Cannabis users present different athletic performance and recovery (Docter et al., 2020). Based on the literature, Cannabis does not appear to be an enhancer to affect performance, neither is known regarding the use among elite athletes (Ware et al., 2018). Recently, the discussion has been centered on CBD, a phytocannabinoid that was removed from the list of prohibited substances – in or out of competition – from the World Anti-Doping Agency (WADA) and the United States Anti-Doping Agency (USADA). Although CBD is not prohibited, athletes should be alerted some CBD oils and tinctures extracted from Cannabis plants may also contain THC and other cannabinoids that could result in a positive test for a prohibited cannabinoid.^1^

## Meditation

Meditation is a multifaceted process that allies strength, endurance, flexibility and enables self-control to create an awareness of concentration, calmness, and well-being, presenting both physical and mental health benefits (Woodyard, 2011). It impacts cognition, memory, social, and emotional control, which enhances the autonomic control of the nervous system and peripheral targets as cardiovascular, neuroimmune, and renal physiology (Jindal et al., 2013). Mind-body exercises control several brain structures, altering neural activity and functional connectivity, predominantly in the prefrontal cortex, hippocampus/medial temporal lobe, lateral temporal lobe, insula, and the cingulate cortex (Zhang et al., 2021). Although the molecular mechanisms involved are not fully understood, it is clear that several transmitter systems and brain areas are involved (Jindal et al., 2013) and the ECS has gained attention in the pursue of happiness or treat diseases (Ghaffari and Kluger, 2014; Sadhasivam et al., 2020; Tsuboi et al., 2020). Regular mindfulness practice has consequences on physiological and psychological functioning with performance outcomes in sports (Bühlmayer et al., 2017) and regular yoga has improved sleep quality and work stress (Fang and Li, 2015). Data on regular yoga users (transcendental meditation) faced modest average reductions in blood pressure (Brook et al., 2013). Depression or anxiety have also been alternatively treated with non-conventional interventions, including exercise, yoga and meditation (Cramer et al., 2013; Field et al., 2013; Saeed et al., 2019). In patients with mild-to-moderate Parkinson’s disease (PD), mindfulness yoga has been shown to be effective in improving motor dysfunction and mobility (Kwok et al., 2019). Curiously, a novel concept labeled as *Spiritual Fitness* which pursues stress reduction, basic and psycho/spiritual wellbeing is being used in AD prevention (Khalsa and Newberg, 2021). Adults under cancer treatment have also gained benefits under yoga practice for improving psychological outcomes, possibly also improving physical symptoms (Danhauer et al., 2017); however, more rigorous and large groups designed randomized trials are needed (Ford et al., 2020) to address the psychosocial needs of cancer patients.

From a millennial background in the Indian culture with a focus on the four foundations of mindfulness – *body, feelings, mind, and dhammas* – the sense of truth, healing named as yoga, meditation has become widely praised in the Western societies, including used as medical and psychological therapies for stress-related physical and mental disorders (Woodyard, 2011). Although the biological mechanisms in terms of the effect on the brain and body are poorly understood, the molecular correlates of these effects operate through the major neurochemical system, amines (acetylcholine, dopamine, and serotonin) and the putative release of endogenous cannabinoids and endorphins, which may exert salutary effects on mood/anhedonia (Muzik and Diwadkar, 2019). In a double-blind, randomized, placebo-controlled study with 15 healthy experienced mindfulness meditation practitioners, participants rated the pain of a cold stimulus before and after a mindfulness meditation session. Participants were randomized to receive either intravenous naloxone or saline, after which they meditated again, and rated the same stimulus. The conclusion was that meditation involves endogenous opioid pathways mediating its analgesic effect, which could hold promising therapeutic implications and elucidation for the mechanisms involved in human pain modulation (Sharon et al., 2016).

**FIGURE 5:**
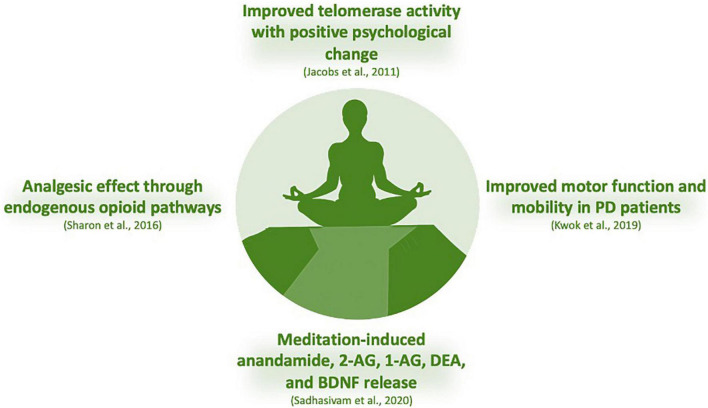
Regular mindfulness practice has consequences on physiological and psychological functioning with performance outcomes in sports, improving sleep quality and work stress. Data on regular yoga users (transcendental meditation) faced modest average reductions in blood pressure. Depression or anxiety have also been alternatively treated with non-conventional interventions, including exercise, yoga, and meditation. In addition, patients with mild-to-moderate Parkinson’s disease, mindfulness yoga has been shown to be effective in improving motor dysfunction and mobility. An experimental study conducted on adults before and after the 4-day Isha Yoga Bhava Spandana Program evaluated AEA, 2-AG, 1-arachidonoylglycerol (1-AG), DEA, oleoylethanolamide (OLA), and BDNF on anxiety and depression through psychological scales. Authors reported changes in major eCBs levels, with increase in AEA, 2-AG, 1-AG, DEA, and BDNF after meditation, suggesting a participation for these biomarkers in the underlying mechanism of meditation (Sadhasivam et al., 2020). Indeed, increased BDNF levels has been linked in meditative practices and brain health in a 3-month yoga and meditation retreat assessed with psychometric measures, circadian salivary cortisol levels, and pro- and anti-inflammatory cytokines (Cahn et al., 2017). In addition, a 3-month meditation retreat has been evaluated on telomerase activity and the experience of stress, with participants controlled in concentrative meditation techniques and collection of peripheral blood mononuclear cell samples for telomerase activity. Authors reported a clear link between meditation and positive psychological change with telomerase activity (Jacobs et al., 2011).

It is suggested that volitional changes in breathing patterns can activate primary control centers for descending pain/cold stimuli in periaqueductal gray, initiating a stress-induced analgesic response mediated by eCB/endorphin release. The analgesic effects and the feelings of euphoria generated by eCB release are prolonged *via* a top-down “outcome expectancy” control mechanism regulated by cortical areas (Muzik and Diwadkar, 2019). An experimental study conducted on adults before and after the 4-day Isha Yoga Bhava Spandana Program evaluated AEA, 2-AG, 1-arachidonoylglycerol (1-AG), DEA, oleoylethanolamide (OLA), and BDNF on anxiety and depression through psychological scales. Authors reported changes in major eCBs levels (Figure 5), with increase in AEA, 2-AG, 1-AG, DEA, and BDNF after meditation, suggesting a participation for these biomarkers in the underlying mechanism of meditation (Sadhasivam et al., 2020). Indeed, increased BDNF levels has been linked in meditative practices and brain health in a 3-month yoga and meditation retreat assessed with psychometric measures, circadian salivary cortisol levels, and pro- and anti-inflammatory cytokines (Cahn et al., 2017). In addition, a 3-month meditation retreat has been evaluated on telomerase activity and the experience of stress, with participants controlled in concentrative meditation techniques and collection of peripheral blood mononuclear cell samples for telomerase activity. Authors reported a clear link between meditation and positive psychological change with telomerase activity (Jacobs et al., 2011).

The suggested participation of the ECS on health benefits of meditation may have direct and undirect roles of the cannabinoid signaling. The undirect effects may arise from the ECS regulation upon the “stress axis” hypothalamus-pituitary-adrenal (HPA) that controls glucocorticoid (cortisol or corticosterone) release (Gjerstad et al., 2018). Corticotropin-releasing hormone (CRH) neurons of the paraventricular hypothalamic nucleus (PVN) receive and integrate inputs coming from brain areas comprising the limbic system that are responsible for processing psychological stressors, such as pre-frontal cortex, medial amygdala, paraventricular thalamic nucleus, among others (Herman et al., 2002). The ECS is widely expressed in all components of the limbic system and HPA axis (Micale and Drago, 2018). The afferences from the limbic system establish synaptic contact with local interneurons of the PVN that inhibit or stimulate the CRHergic neurons through GABAergic or glutamatergic synapses (Darlington et al., 1989; Herman et al., 2002; Camille Melon and Maguire, 2016). Recent studies demonstrated that the eCBs act like a gatekeeper of the HPA axis, decreasing the activity of the CRHergic neurons tonically, acting like a stress-buffer system (Micale and Drago, 2018).

Experimental evidence supports the buffering role of the ECS on stress response. Treatment with CB1 antagonist (SR141716A) results in increased corticosterone release in mice (Wade et al., 2006) and, in agreement, CB1 knockout mice have increased basal secretion of adrenocorticotropin hormone and corticosterone (Barna et al., 2004). Additionally, a mice model of CB deficiency (*Cnr*^–/–^) is highly sensitive to chronic social defeat stress protocol with altered glucocorticoid levels, suggesting dysregulation of the HPA axis (Beins et al., 2021).

## Endocannabinoid System Meets Mitochondria: Relevance for the Brain

In order to maintain its cellular processes (including neurotransmission, protein and lipid synthesis, and others), CNS presents a high metabolic activity. Therefore, continuous energy and oxygen supply is required (McKenna et al., 2019). Mitochondria are then pivotal for normal brain function. Despite the notorious role of mitochondria for cellular energetics and redox homeostasis, these organelles are also involved in a myriad of other physiological and pathophysiological mechanisms in the cells (Niquet et al., 2006; Thornton and Hagberg, 2015; Devine and Kittler, 2018; Belenguer et al., 2019). Mitochondria respond in a dynamic fashion to cope with cellular demands (Bénard et al., 2012; Labbé et al., 2014).

Although the mainstream signaling of CBs initiates at the plasma membrane and invades the cytoplasm and intracellular organelles, CB1 expression is predominantly intracellular (Rozenfeld, 2011). Functional CB1 receptors are found on intracellular compartments as endosomes (Rozenfeld and Devi, 2008) and mitochondria (Bénard et al., 2012). The biological relevance of this unorthodox localization of CB1 receptors, particularly in mitochondria, is still a matter of debate.

The seminal work from Bénard et al. (2012) showed that either endocannabinoids or exogenous cannabinoids can activate mitochondrial CB1 receptors in the brain. Such activation tones down respiration, as well as PKA activity and the intramitochondrial levels of the second messenger cyclic AMP (cAMP). Genetic manipulation tools allowed the observation that the activation of mitochondrial CB1 receptors in the hippocampus leads to memory impairment (Hebert-Chatelain et al., 2016). Activation of the astrocytic mitochondrial CB1 receptors decreases glucose metabolism and lactate formation in the brain, impacting neuronal functioning, and animal behavior (Jimenez-Blasco et al., 2020). It should be considered that higher brain functions present demanding energy budget and mitochondria are pivotal to the maintenance of brain bioenergetics and the metabolism of neurotransmitters (Dienel, 2019). The ECS system seem to be differentially affected depending on the stage of brain development (Volkow et al., 2014; Alpár et al., 2016; Bara et al., 2021), which represents a period of challenging metabolic demands (McKenna et al., 2015). Therefore, these observations indicate that alterations on mitochondrial CB1 receptors in the brain could represent a novel therapeutical tool, as well as a possible mechanism underlying the behavioral alterations elicited by cannabinoid consumption.

It has been reported that the levels of the eCB 2-oleoylglycerol are low in the brain of mice lacking carnitine palmitoyltransferase-1c (CPT1c) (Lee and Wolfgang, 2012). CPT1c modulates energy homeostasis (Wolfgang et al., 2006) and shows high homology with the isozymes CPT1a and CPT1b but is restricted to neurons (Price et al., 2002). Whilst CPT1a and CPT1b are found in mitochondria, where they bind acyl moieties to carnitine (Ferreira and McKenna, 2017), CPT1c is expressed in endoplasmic reticulum and its biological function is still uncertain (Sierra et al., 2008). Whether on the one hand it is still to be defined if the effect of CPT1c on the metabolism of endogenous cannabinoids is either direct or indirect, on the other hand recent reports implicate CPT1c with mitochondrial function (Wang et al., 2020; Chen et al., 2021). Mitochondrial adaptations also seem to be involved in the modulation of feeding behavior elicited by ligands of CB1 receptors (Koch et al., 2015); for a deeper discussion on cannabinoids affecting feeding behavior refer to the section “Endocannabinoid System and the Neuroendocrine Regulation of Energy Metabolism.”

## Cannabis and the Endocannabinoid System

Cannabis use dates to the ancient Eurasian societies, with evidence pointing to the territories of modern China and Romania as the oldest sites of Cannabis use (plant and seeds) for general purposes (Holland, 2010). A recent study found burned traces of the plant in wooden braziers from a cemetery in western China. The artifacts dated back 2500 years (500 BCE). Further phytochemical analyses revealed an abundance of psychoactive compounds in the samples, thus suggesting that Cannabis was smoked as a way of provoking ritualistic or religious experiences (Ren et al., 2019). Since then, Cannabis consumption has undergone a series of social transformations, going from a frequently prescribed medicine up until the first decades of the 20th century to a highly illegal drug. Cannabis is regaining space in health care (Cunha et al., 1980), quite possibly, starting a wave of legal precedents toward recreational use worldwide (Bridgeman and Abazia, 2017). How does Cannabis modulate the ECS? Which are the main consequences of marijuana consumption on the classically recognized ECS properties?

An important concept to have in mind while discussing Cannabis is that plants have been the main source of medicine prior to the industrial revolution. In such context, the overall effects of *C. sativa* in the human body were well-known long before it elicited any interest from the scientific community. In general, Cannabis consumption extenuates the physiological effects attributed to the ECS, that is, “relax, eat, sleep, forget and protect” (Di Marzo, 1998). After a single administration of THC, it rapidly migrates from the blood to the brain and other high perfusion tissues. Then, it takes up to 2 days for the substance to reach the highest concentrations in low perfusion tissues, and up to 10 days until it is fully stored in the adipose tissue (Blesching, 2020). Coinciding with the presence of THC in the brain, the psychotropic effects of Cannabis also start within minutes after use and can last for a few hours.

The first and most reported effects of Cannabis start right after consumption, and they are highly associated with the relaxation component of the ECS. Effects on mood are generally described as feelings of decreased anxiety, alertness, depression, and tension. Also, it seems to promote sociability if the user is exposed to a favorable environment. At higher doses, Cannabis users may experience somewhat opposed effects such as increased anxiety, paranoia, psychosis, and panic. Perception of color, time, and space are also distorted, and may include hallucinations with high doses. Decreased cognitive and motor skills were repeatedly shown to affect even the experienced user, increasing by a significant proportion the risk of motor vehicle accidents. The consumption of alcohol and other CNS depressants are additive to the cognitive and motor effects of Cannabis, as reviewed by Ashton (2001).

The ability of Cannabis to positively regulate food intake in humans raised considerable controversy in the 1970s, mainly because most animal-derived data pointed toward decreased instead of increased consumption after exposure to marijuana (Abel, 1975). Later, investigations revealed that, in fact, after crossing a certain threshold of Cannabis, humans tend to increase their daily food intake by up to 1,000 calories. Interestingly, in all scenarios examined the high caloric consumption was achieved through eating more snacks instead of bigger meals (Foltin et al., 1986). Indeed, chronic Cannabis use can increase adiposity and insulin resistance, possibly through its sustained orexigenic properties (Muniyappa et al., 2013). More recent studies with knockout animals and receptor antagonists were able to include the cannabinoid and endocannabinoid agents in the orexigenic substance category (Kirkham and Williams, 2001). Hypothalamic centers are stimulated by phytocannabinoids to induce food-seeking behavior and to modulate hormone release (Pacher et al., 2006). In the nucleus accumbens it increases motivation for palatable food. Finally, cannabinoids control several endocrine mechanisms in the liver, adipose tissue, muscles, and the gastrointestinal tract, as reviewed by Pagotto et al. (2006).

Regarding the sleep-inducing effects of Cannabis, studies have yielded mixed results. This apparent heterogeneity may stem from the variable THC/CBD proportions and concentrations found in Cannabis samples across studies. Overall, research indicate that marijuana consumption has short-term benefits for the treatment of sleep conditions, and that it progressively builds tolerance on the user up to a point where chronicity causes complete habituation. Some suggest that a higher proportion of CBD may reduce tolerance and extend the sleep benefits of cannabinoid-based treatments (Babson et al., 2017). In fact, phase I–III studies with a 1:1 THC:CDB compound showed improved sleep quality for patients with relevant pain conditions (Russo et al., 2007). On a different perspective, sleep deprivation is shown to correlate with increased likelihood of marijuana use among teenagers, revealing that the benefits associated with marijuana have reached public knowledge and may influence behavior and substance use (Choi et al., 2020). Another highly reported effect of marijuana is its ability to impair short- and long-term memory. Schwartz et al. (1989) found that teenagers exposed to marijuana develop short-term memory deficits lasting for up to 6 weeks, which provided support to the previous evidence and helped consolidate the clinical landscape for adolescent Cannabis consumption (Deahl, 1991). A newly published meta-analysis confirmed a relevant association between marijuana and both short- and long-term memory loss. The study, however, highlights that the effect sizes emerging from these correlations were considerably small, suggesting a contrast with neuroimaging studies associating Cannabis-induced memory loss and the structural changes found in areas such as the hippocampus (Figueiredo et al., 2020). In addition to memory impairment, it is reasonable to associate Cannabis with problems in attention and the ability to process complex information. This effect may persist for weeks, months, or years, depending on the chronicity and frequency of use (Solowij and Michie, 2007). In a functional magnetic resonance study from 2006, working memory and selective attention of frequent but moderate Cannabis users were compared to that of healthy non-users. Except for an alteration in brain activity on the left parietal superior cortex, researchers found no support for the hypothesis of memory and attention deficits emerging from moderate Cannabis use (Jager et al., 2006). A later review of the evidence regarding the chronic effects of marijuana abuse has shown that, although most effects emerging from the plant are acute and tend to fade away with time, there seems to exist some risk of decision-making impairment for the long-term heavy user (Crean et al., 2011).

Some Voxel-based morphometry studies, on the other hand, have shown that chronic users may be subject to reduction of gray matter (GM) in several areas of the brain. For instance, a decreased GM was reported on the medial temporal cortex (cognitive and emotional functions), temporal pole (emotional and social behavior), parahippocampal gyrus (spatial memory), insula (roles in addiction and psychiatric disorders), and the orbitofrontal cortex (emotion and memory) of regular Cannabis users (Battistella et al., 2014). Demirakca and coworkers investigated GM from the hippocampus of recreational marijuana users. Accordingly, the group found reduced GM volume on the right anterior hippocampus, with further correlation analyses showing a potential protective role for CBD among study participants (Demirakca et al., 2011).

Corroborating with (Battistella et al., 2014), a study from Filbey et al. (2014) showed that chronic exposure to marijuana reduces the volume of GM in the orbitofrontal cortex. Further, the brain of frequent Cannabis users revealed complex structural changes emerging as a function of onset and duration of use (Filbey et al., 2014). Despite these reported alterations, the debate remains on whether they are fully or partially reversible under complete abstinence. This is especially important for patients presenting with PTSD and chronic pain, conditions in which treatment with Cannabis is effective, but may promote tolerance after prolonged use (Cuttler et al., 2020; LaFrance et al., 2020).

Finally, an important discussion is necessary when long-term effects on cognition of medical cannabis (MC) use is compared to recreational cannabis, especially in those with adolescent onset. Comparison of MC patients from recreational consumers considers diverse factors as motives for use, product selection, and age of onset, and a recent study evaluated cognitive and clinical measures in well-characterized MC patients over 1 year (Sagar et al., 2021). MC patients finalized a baseline visit prior to initiating MC and evaluations following 3, 6, and 12 months of treatment, performing a neurocognitive battery assessing executive function, verbal learning/memory, and clinical scales assessing mood, anxiety, and sleep. Exposure to THC and CBD was also measured. Authors reported that MC patients exhibited significant enhancements on measures of executive function and clinical state over the course of 12 months; clinical improvement was associated with higher CBD use. Therefore, MC patients may show improvements rather than impaired executive function over time (Sagar et al., 2021). As Cannabis research remains in its infancy (Zolotov and Gruber, 2021), further studies are necessary to evaluate differences between recreational and MC use to identify potential mechanisms related to cognitive changes and the role of clinical improvement.

Although the molecular mechanisms underlying Cannabis-induced cognitive deficits are still elusive, three mechanisms have been proposed as necessary for these effects to emerge. First, hippocampal activation of CB1 receptors seems to be more pronounced on GABAergic than on glutamatergic populations of neurons, thus inducing excess activation of glutamate receptors in the hippocampus, which leads to molecular signals that impair cognitive processing. Second, cannabinoids interfere with choline, adenosine (A2 receptors) and serotonin signaling, thus affecting the fine tuning of memory consolidation. Third, the decrease in cell metabolism by activation of mitochondrial CB1 receptors may extenuate the first and second mechanisms (Prini et al., 2020).

## Fighting Neurodegenerative Diseases With a Strong Endocannabinoid System

There is a growing interest to reveal novel active compounds in the pharmaceutical field to improve health and longevity of the elderly population. The average life expectancy of the global population increased to 80 years in the developed countries, compared to 50 years in the early 20th century (Jin et al., 2014). People can expect to live into their 60s and beyond, as a result in large reductions in mortality at younger ages. As high-income countries continue to increase in life expectancy among those who are older, a child born in Brazil can expect to live 20 years longer than one born just 50 years ago (World Health Organization, 2015). However, rising life expectancy in developed countries has as consequence the emergency of primary risk factors for neurodegenerative diseases associated with aging. Aging is the primary risk factor for most neurodegenerative diseases, and one in 10 individuals aged more than 65 years manifest symptoms of AD and its prevalence continues to advance with increasing age. PD and AD are among the most common neurodegenerative disorders, affecting millions of people worldwide (Selkoe, 2011; Wirdefeldt et al., 2011; Tysnes and Storstein, 2017). Both diseases have no cure, thus the current treatments only reduce the main symptoms. In this sense, searching new targets to prevent and/or impair the progression of these diseases is highly desirable.

Components of the ECS are expressed in the basal ganglia neural circuits, which modulate dopaminergic, GABAergic and glutamatergic signaling. This network is specially impaired during PD due to death of dopaminergic neurons of the *substantia nigra pars compacta* (SNpc) (Dauer and Przedborski, 2003; Benarroch, 2007). Disturbances in the ECS homeostasis have already been observed in cerebral areas associated with PD pathology in humans, as well as in animal models. CB1 receptor mRNA is reduced in basal ganglia of *post-mortem* brain of individuals with PD (Hurley et al., 2003); in addition, levels of AEA are increased in the cerebrospinal fluid in untreated PD patients endogenous (Pisani et al., 2005). Similarly, in the 6-hydroxydopamine (6-OHDA)-induced lesion model in rats the expression of the CB1 receptor was significantly reduced in the *substantia nigra pars reticulata* (SNpr) (Walsh et al., 2010), while CB2 receptor increased in the striatum, followed by an enhance in microglial and astrocyte activation (Concannon et al., 2015). Additionally, using the same animal model, AEA levels are increased while FAAH activity is reduced in the striatum, supporting a boost of the ECS, and probably reflecting a compensatory mechanism to counteract chronic dopamine depletion (Gubellini et al., 2002). Similarly, as Huntington’s disease progress, CBs are also severely reduced in all regions of the basal ganglia implying a potential role for cannabinoids in the progression of neurodegeneration in Huntington’s disease (Glass et al., 2000; Scotter et al., 2010).

Modulatory effects of the ECS in nigrostriatal pathway support studies targeting this system as a therapeutic strategy in PD. In animal models of PD, CB1 or CB2 synthetic agonists as well as inhibitors of FAAH or MAGL improved motor impairments and induced neuroprotection (Price et al., 2009; Fernández-Suárez et al., 2014; Celorrio et al., 2016; Javed et al., 2016). Likewise, treatment with CBD also enhances neuroprotection, both *in vitro* and *in vivo* (Lastres-Becker et al., 2005; García-Arencibia et al., 2007; Santos et al., 2015).

In open-label observational studies, smoking Cannabis improved motor symptoms, such as tremor, rigidity, and bradykinesia in Parkinson’s patients, and ameliorated sleep and pain scores (Lotan et al., 2014; Shohet et al., 2017). Moreover, purified CBD has shown to produce positive effects specially to treat non-motor symptoms of PD, improving quality of life and mental health in patients (Zuardi et al., 2009; Chagas et al., 2014).

The roles of the ECS regulating immune and cognitive functions also support its modulation as a potential novel therapeutic target in AD. Nevertheless, findings regarding CB1 receptor expression in this disease are still unclear and the outcomes variable. In the 3×Tg-AD transgenic mice model, CB1 mRNA is increased in the prefrontal cortex, dorsal hippocampus, and basolateral amygdala complex, while decreased in ventral hippocampus of animals with 6 and 12 months of age, but not at 2 months of age (Bedse et al., 2014). Interestingly, it was observed in human AD brain samples an hyperactivation of CB1 receptor in earlier stages and a progressive decrease in advanced stages of the disease (Manuel et al., 2014). These results suggest that alterations in the ECS might be age and/or pathology-dependent, indicating a relevant issue to be considered in therapeutic approaches. In contrast, other studies showed that CB1R immunocontent was unchanged in different cortical regions and hippocampus of human *post-mortem* samples, and in cortical areas evaluated by positron emission tomography in individuals with AD pathology (Lee et al., 2010; Mulder et al., 2011; Ahmad et al., 2014).

On the other hand, CB2R, MAGL, and FAAH are increased adjacent to senile amyloid plaques associated with microglia and/or astrocytes, exhibiting positive correlation with Alzheimer’s progression and probably regulating inflammatory mechanisms (Benito et al., 2003; Mulder et al., 2011). In fact, activation of CB2 receptor protects hippocampal neurons from Aβ1-42 toxicity (Zhao et al., 2020). Otherwise, transgenic amyloid mice lacking CB2 receptor expression present an increase in plaque deposition and plaque-associated microglia, in addition to high soluble Aβ42 levels in the brain (Koppel et al., 2014). Additionally, cannabinoid agonists (HU-210, WIN55,212-2, and JWH-133) and JZL184, a MAGL inhibitor, have anti-inflammatory and neuroprotective effects, decreasing microglia effects and reducing the total Aβ burden *in vitro* (Ramírez et al., 2005) and its precursors in the APdE9 mouse model (Ramírez et al., 2005; Pihlaja et al., 2015). ACEA (arachidonyl-2-chloroethylamide), a selective cannabinoid CB1 receptor agonist, also increases cortical neurons viability exposed to Aβ-42 oligomers, inducing cognitive improvement in AβPP/PS1 mice. These effects are correlated with a decreased astroglial reactivity and production of pro-inflammatory proteins, since ACEA did not impair Aβ aggregation (Aso et al., 2012).

Similarly, CBD and THC have demonstrated neuroprotection in chronically treated AβPP/PS1 mice showing improvements in memory tasks and a decrease in soluble Aβ42 levels, astrogliosis, and several neuroinflammation markers (Aso et al., 2015). Also, CBD alone prevented the development of a social recognition deficit in the same animal model (Cheng D. et al., 2014). Furthermore, *in vitro* assays showed that CBD shows neuroprotective effects in PC12 cells through Wnt/β-catenin pathway in Aβ-induced toxicity model (Esposito et al., 2006).

Although the current findings still do not validate a direct effect of the cannabinoid-based medicine in memory or cognition in AD patients, other symptoms might be alleviated using this approach. Data from mice suggest that treatment with CB1 receptor antagonists might restore memory capacity in animals administered with beta-amyloid fragments that lead to memory disturbances (Mazzola et al., 2003). Alternatively, VDM-11, an inhibitor of eCB cellular reuptake, increased rat hippocampal and mouse brain eCB levels, reversing hippocampal damage in rats, and loss of memory retention in the passive avoidance test in mice, when administered from the 3rd day after beta-amyloid peptide (1–42) injection (van der Stelt et al., 2006). Therefore, early, as opposed to late, pharmacological enhancement of brain eCB levels might protect against beta-amyloid neurotoxicity and its consequences, reviewed in Micale et al. (2007). In severely demented patients, a prospective observational study showed that the use of oral Cannabis extract, with THC/CBD, significantly improved behavior problems, reducing rigidity, and simplifying daily care (Broers et al., 2019). Moreover, medical Cannabis oil enriched in THC has differential effects on the Neuropsychiatric Inventory (NPI) scale, probably dependent on the duration and dosage (van den Elsen et al., 2015; Shelef et al., 2016).

Taken together, a growing number of studies have demonstrated beneficial effects of the ECS activation which has proven an excellent target for the treatment of neurodegenerative disease, reducing significative symptoms and improving well-being in these individuals.

## Endocannabinoid System and the Neuroendocrine Regulation of Energy Metabolism

### Endocannabinoid System and the Hypothalamus – Adipose Tissue Axis in Obesity

Obesity is a major health issue (Kelly et al., 2008) and no country succeeded in decreasing the number of obese individuals in the last decades, indicating the limitation of the worldwide public health policies (Burgio et al., 2015). The obesity etiology is multifactorial with interactions of the genetic background and environmental cues (malnutrition, poor PA, toxicant exposure, and stress) that result in unfavorable metabolic phenotype (Rohde et al., 2019). Obesity results from an imbalance between energy intake and expenditure, with the hypothalamus as a major regulator in the CNS.

The hypothalamus is an evolutionarily ancient part of the brain and acts as an integrating node since peripheral inputs are brought together in this region (Burbridge et al., 2016). The hypothalamus is a master homeostatic regulator, capable of modulating activities that are crucial to life, such as energy homeostasis (Roh et al., 2016) and glycemic control (Pozo and Claret, 2018). Interestingly, in obesity and the high-fat diet intake, afferent signals can be differently received and sensed by subsets of hypothalamic nuclei, contributing to the development of metabolic disorders (Formolo et al., 2019).

The hypothalamus receives information on the status of body energy storages through sensory innervation and hormone secretion mainly from the white adipose tissue (WAT) and gastrointestinal tract (Roh et al., 2016). In this context, the adipocyte-derived hormone leptin is a key factor because it is produced according to nutritional status and adipose tissue mass (Friedman, 2019). Leptin activates subsets of hypothalamic neuronal populations, inducing an anorexigenic effect, increasing energy expenditure, and acting as an antidiabetic signal (Bouret et al., 2004; Pozo and Claret, 2018). Obese subjects frequently present hyperleptinemia but leptin resistance (Dragano et al., 2017), which contributes to the positive energy balance due to several mechanisms, including the over activation of the ECS signaling in the CNS (Thanos et al., 2008; Cristino et al., 2013) and adipose tissue (Sarzani et al., 2009).

Hypothalamic leptin action leads to phosphorylation of STAT3 (signal transducer and activator of transcription 3) (pSTAT3) and is especially critical for hypothalamus maturation (Bouret et al., 2004). Interestingly, leptin-deficient obese mice or diet-induced obese mice present increased levels of CB1 and DAGL in the lateral hypothalamus (Cristino et al., 2013), evidencing an inverse relationship between leptin and ECS signaling.

In a rat model of maternal obesity, maternal high-fat diet intake during pregnancy down-regulates hypothalamic STAT3 in neonate rat offspring associated with hypoleptinemia only in male pups. This profile occurred in parallel to increased levels of CBs in the hypothalamus of the neonate offspring (Dias-Rocha et al., 2018; Almeida et al., 2019). Surprisingly, ECS changes were observed before obesity and hyperinsulinemia development in the offspring (Almeida et al., 2017) and remains until adulthood (Dias-Rocha et al., 2018; Almeida et al., 2020). The maternal high-fat diet also increases plasma –n-6/n-3 ratio in newborn rat offspring (Almeida et al., 2019), which might indicate an increased risk factor for metabolic disorders and over activation of the ECS signaling (Freitas et al., 2018). This profile also suggests a disruption in the brain-adipose tissue axis for appetite regulation, since the adipose tissue lipid profile impacts the local eCB content, while the FAAH inhibition leads to diet-induced hyperphagia in adult genetically modified mice (Li et al., 2018).

The overall balance between the anorexigenic and orexigenic hypothalamic neuropeptides defines the final metabolic outcome, and CB1 activation modulates feeding by enhancing the orexigenic signals and preference for fat (D’Addario et al., 2014; McGavin et al., 2019). In a mice model, the CB1 activation specifically in hypothalamic proopiomelanocortin (POMC) neurons increases food intake by increasing selectively the production of β-endorphin, an orexigenic peptide involved in reward, from POMC cleavage (Koch et al., 2015). In leptin-deficient obese mice, CB1-expressing presynaptic neurons change from glutamatergic to predominantly GABAergic in the lateral hypothalamic area, and because CB1 is associated with G_*i/o*_ protein, this remodeling results in increased orexin-A, an orexigenic peptide (Cristino et al., 2013). In rats, the CB1 activation decreases hypothalamic serotonergic activity, an important satiety signal, and induces disinhibition of GABA release to stimulate food intake (Cruz-Martinez et al., 2018).

Cannabinoid receptor type 1 activation promotes the conservation of energy not only promoting food intake by hypothalamic mechanisms but also inhibiting energy expenditure by reducing the uncoupling protein 1 (UCP1) expression and thermogenesis in the brown adipose tissue (BAT), which favors the WAT expansion (Maccarrone et al., 2015). CB1 density in the brain, BAT, and WAT of overweight subjects are modified compared to lean subjected, reflecting the impairment of ECS in obesity (Lahesmaa et al., 2018). CB1 signaling also activates lipogenesis and adipogenesis in WAT depots (Maccarrone et al., 2015; Ruhl et al., 2020), such as visceral (VIS) and subcutaneous (SUB), which present structural and functional differences associated with a CB1 depot-specific expression. The VIS WAT expansion is a greater predictor of mortality than SUB WAT excess (Ibrahim, 2010). CB1 expression is lower in VIS WAT than in SUB WAT of lean subjects, while there is no differential CB1 expression between WAT depots in obese subjects (Bennetzen et al., 2010). CB1 gene expression is twofold higher in SUB WAT from type 2 diabetes subjects, as compared to control subjects (Sidibeh et al., 2017).

Regarding the role of CB2 in energy metabolism, its role as a pro- or anti-inflammatory in the central and peripheral tissues is controversial (Ueda et al., 2007; Deveaux et al., 2009; Chiurchiu et al., 2014; Maccarrone et al., 2015; Verty et al., 2015). Studies have reported a CB2 anti-obesity effect by silencing the activated immune cells in mice adipose tissue (Verty et al., 2015; Notarnicola et al., 2016), as well as a diet enriched with olive oil as responsible for increasing CB2 receptor expression in this tissue (Notarnicola et al., 2016).

### Programming of the Endocannabinoid System During Early Life

**FIGURE 6:**
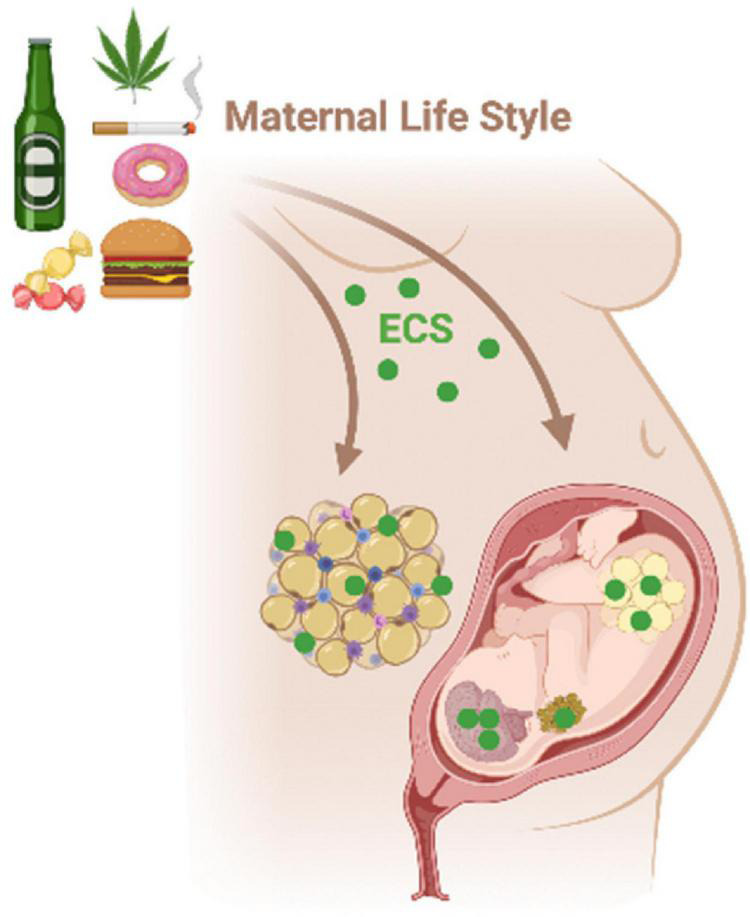
Maternal environmental insults such as diet, smoking, and alcohol consumption during critical periods of life, as gestation, may affect the ECS in the hypothalamus-adipose axis of the mother and the progeny. The ECS disruption during early life can program metabolism in a short- and long-term way increasing the risk to develop metabolic syndrome and behavioral changes throughout life.

The tonus of the ECS in the brain and peripheral tissues may be modulated by unappropriated parental life style and environmental conditions (nutrition, toxicant exposure, and stress) during the perinatal period and adolescence, predisposing offspring to metabolic and behavioral disorders throughout life (Figure 6; Lopez-Gallardo et al., 2012; Stringer et al., 2013; Ramirez-Lopez et al., 2015, 2016a,b; Romano-Lopez et al., 2016; Almeida et al., 2017, 2019, 2020; Dias-Rocha et al., 2018; Gandhi et al., 2018; Miranda et al., 2018; de Oliveira et al., 2019; Soares et al., 2019; Rivera et al., 2020). This phenomenon is known as “metabolic programming” or “ontogenetic plasticity” and involves epigenetic regulation of gene expression (Brenseke et al., 2013; Lillycrop and Burdge, 2015; Gluckman et al., 2019).

Maternal obesity/overweight and hypercaloric diet consumption are major concerns for metabolic programming. Two-thirds of American women at reproductive age are overweight, which represents a risk for their own health and the following generations (Stang and Huffman, 2016).

Maternal high-fat (45% fat) diet decreases serum levels of eCBs in baboon offspring at birth (Gandhi et al., 2018). In rats, a maternal hypercaloric-low protein diet (6% protein, 24% fat) decreases hypothalamic endocannabinoid levels only in newborn male offspring, while decreases the preference for a chocolate diet and induces anxiety-like behavior in these animals in adulthood (Ramirez-Lopez et al., 2015, 2016a). In a rat model, maternal isocaloric high-fat (29% fat) diet increases hypothalamic CB1 and CB2 expression in newborn male and female offspring, respectively, while increasing CB1 expression in BAT only in male offspring at birth (Dias-Rocha et al., 2018; Almeida et al., 2019). In addition, maternal high-fat diet induces a differential regulation of CB1 content between visceral and subcutaneous WAT, suggesting redistribution of fat storages favoring visceral depot (Almeida et al., 2017). These ECS changes occurred in parallel to alteration of molecular markers of adipogenesis, lipogenesis, and thermogenesis across the adipose tissue depots of offspring at weaning (Almeida et al., 2017). Interestingly, there are sex-specific molecular signatures in the offspring at early life, but high-fat offspring of both sexes develop obesity, hyperphagia, and a higher preference for fat in adulthood (Dias-Rocha et al., 2018; de Almeida et al., 2021).

Of note, although many benefits of phytocannabinoids have been discussed in the context of neurodegenerative diseases and stress relief, the use of Cannabis during critical periods of development such as gestation and lactation as well as during adolescence may be harmful. Prenatal Cannabis exposure predicts fetal growth restriction, preterm delivery, and neonatal intensive care (Nashed et al., 2020). In human term placenta, THC increases AEA levels, which might be detrimental for the balance of trophoblast cells turnover leading to alterations in normal placentation and fetal growth (Maia et al., 2019). In pregnant rats, Cannabis exposure reduces placental fetal capillary area and increases collagen deposition, these changes occur in parallel to reduced glucose transporter 1 expression in the labyrinth which may account for intrauterine growth restriction (Natale et al., 2020).

Tetrahydrocannabinol crosses the placenta and binds to fetal CBs, changing neurodevelopment and possibly predisposing the offspring to abnormalities in cognition and emotion in humans and animal models (Richardson et al., 2016; De Genna et al., 2021). In mice, THC exposure from embryonic day 12.5 to embryonic day 16.5, a critical window for corticospinal motor neuron development, results in a transient decrease in CB1 content and binding in whole embryonic brain that is rescued by postnatal day 2. These alterations are associated with increased seizure susceptibility in adult offspring (de Salas-Quiroga et al., 2015). Interestingly, embryonic THC exposure selectively decreases CB1 in the hippocampus of male mice at postnatal day 20 in parallel to decreased CB1-expressing GABA interneurons, resulting in impaired spatial memory in male adult mice (de Salas-Quiroga et al., 2020). In rats, maternal THC exposure from embryonic day 15 to postnatal day 9 decreases CB1 binding in the hippocampus and impairs GABAergic function in adult male offspring (Beggiato et al., 2017) associated with deficits in learning and memory (Campolongo et al., 2007).

The adolescence is also an important vulnerable window of Cannabis exposure because adolescent brain is under relevant developmental plasticity. THC exposure in adolescent rats reduces CB1 content in the PFC and VTA at adulthood and, surprisingly, improves working memory performance in males (Stringfield and Torregrossa, 2021). Interestingly, male adult rats exposed to THC during 10 days in adolescence display increased self-administration of synthetic cannabinoid agonist associated with decreased dopamine levels in the NAc, suggesting addiction-like behavior that occurs in parallel to increased anxiety-like behavior (Scherma et al., 2016).

### Epigenetic Regulation of the Endocannabinoid System

Epigenetics is strongly involved in the regulation of gene expression during early development and in response to psychological, metabolic, and nutritional influences to promote adaptation to environmental challenges throughout life. Epigenetic mechanisms are chemical modifications in the DNA or histones which alter chromatin status and gene transcription levels without changes in the nucleotide DNA sequence.

The main epigenetic marks are DNA methylation and histone acetylation. DNA methylation in the promoter region generally is associated with decreased transcription while histone acetylation shows an opposite fashion of regulation. Lifestyle features such as diet (Di Francesco et al., 2015; Park et al., 2017; Tremblay et al., 2017), exercise (King-Himmelreich et al., 2016; Jonsson et al., 2021), stress (Lomazzo et al., 2017; Cao-Lei et al., 2019), pollutant exposure (Rauschert et al., 2019; Calderon-Garciduenas et al., 2020), drug abuse (Murphy et al., 2018; Grzywacz et al., 2020), and even perinatal environment (Joss-Moore et al., 2015) are known to modulate epigenome in humans and experimental models.

Epigenetics and ECS interplay regulating homeostasis from early embryogenesis to the point-by-point adjustments in adult life (Gomes et al., 2020). Interestingly, the genes encoding the main ECS components (*Cnr1*, *Cnr2*, *Faah*, and *Mgll*) are known to be physiologically regulated by epigenetic mechanisms and in response to diseases or environmental cues (Meccariello et al., 2020).

Most of the studies are focused on the *Cnr1* gene. *Cnr1* contains two exons in the rat and four exons in humans without a classic CpG island (CpG dinucleotide frequency <60%) in the promoter. The methylation levels in the *Cnr1* promoter are relatively high in peripheral tissues or cell types including human and rat colon cells (Di Francesco et al., 2015), and human peripheral blood cells (Rotter et al., 2013), with global promoter methylation ranging from 70 to 95%. In the brain, the *Cnr1* methylation levels are low, as expected, ranging from 10 to 30% (Mancino et al., 2015; D’Addario et al., 2017; Almeida et al., 2019). The inverse association between promoter methylation and *Cnr1* mRNA levels is observed in several studies (Di Francesco et al., 2015; Szutorisz and Hurd, 2016; D’Addario et al., 2017). In humans, there is a progressive decrease in *Cnr1* expression in the hippocampus and PFC from fetal to adult life, which is inversely associated with DNA methylation levels (Tao et al., 2020). In rats, maternal high-fat diet during pregnancy increases the *Cnr1* expression and histone acetylation of its distal promoter in the hypothalamus of male newborns (Almeida et al., 2019). In humans and animal models of schizophrenia, there are higher *Crn1* mRNA levels in blood cells and PFC, respectively, which was associated with decreased methylation in the promoter (D’Addario et al., 2017). Also, the expression of the *Cnr1* gene increases in the PFC of patients with schizophrenia who completed suicide or were exposed to ethanol or THC (Tao et al., 2020). Chronic stress in mice decreases the expression of *Cnr1* in the cingulate cortex associated with reduced levels of histone acetylation (Lomazzo et al., 2017). CB1 activation by AEA is important to regulate the negative feedback and the basal activity of the HPA axis and, during stress, there is a decrease in AEA that may contribute to increased stress response and anxiety behavior (Morena et al., 2016).

### Sex Differences in the Central and Peripheral Endocannabinoid System in Health and Obesity

Body adiposity is under the regulation of both sex steroids and eCBs, and it is well known that males and females present specific fat distribution. While male subjects accumulate fat mainly in the intra-abdominal compartment, females are more likely to accumulate subcutaneous fat (Min et al., 2019). Fat distribution changes throughout life, especially after menopause when females undergo a profound decrease of ovarian hormones with fat redistribution favoring visceral accumulation (Toth et al., 2000).

Interestingly, there are several pieces of evidence pointing to a mutual regulation between ECS and sex hormones in the CNS (Castelli et al., 2014) and peripheral tissues such as the uterus (Maia et al., 2017), gut (Proto et al., 2012), and adipose tissue (de Almeida et al., 2021). In healthy individuals, functional imaging studies have shown that males have more CB1 than females in several brain areas, including the PFC (Laurikainen et al., 2019), a region importantly involved in the hedonic response to palatable foods (Petrovich et al., 2005; Coccurello and Maccarrone, 2018). Additionally, females under treatment with combined oral contraceptives tend to present lower levels of CB1 compared to females with no contraceptives or during menopause, suggesting an inhibitory role of the estrogen levels on ECS (Laurikainen et al., 2019).

Studies in drugs of abuse have shown that women develop Cannabis addiction faster than men (Hernandez-Avila et al., 2004; Cooper and Haney, 2014), a phenomenon that has been confirmed also in experimental models of addiction (Fattore et al., 2007). Estradiol treatment in ovariectomized rats decreased CB1 receptor binding in the PFC (Castelli et al., 2014), evidencing an inverse relationship between estradiol levels and CB also in the rat brain. This association may explain, at least in part, lower CB1 binding in the brain of premenopausal women compared to male individuals.

Experimental studies have also shown that ECS is modulated in a sex-specific manner by several stressful events early in life. Adolescent female rats exposed to maternal deprivation in mid-lactation have increased levels of ECS components in the hippocampus while males exposed to the same insult have an increase of ECS in the PFC (Marco et al., 2014). In a rat model of a maternal high-fat diet during gestation, male offspring have increased CB1 content in the hypothalamus at birth while female offspring had increased CB2 (Dias-Rocha et al., 2018), as mentioned before, highlighting the sex-specific effect of ECS modulation in the early origins of obesity. Adult female rats programmed by maternal high-fat diet early in life have increased CB1 content in WAT associated with decreased estradiol circulating levels, and a similar profile was observed in adipose tissue of ovariectomized adult rats (de Almeida et al., 2021).

The crosstalk between ECS and sex steroid hormones is still reinforced by the characterization of estrogen or androgen response elements in the promoter of the genes encoding the ECS components, suggesting a direct interaction for transcriptional regulation (Grimaldi et al., 2012; Proto et al., 2012; Lee et al., 2013). Therefore, it is important to be aware that nutritional or pharmacological modulation of the ECS to improve health must consider different outcomes depending on sex.

### Endocannabinoid System and Weight Loss

Modulation of the ECS in the CNS and peripheral tissues is an important strategy for weight loss (Quarta and Cota, 2020). Pharmacological CB1 antagonism reduces food intake and preference for palatable foods (rich in fat and sugar) by central mechanisms (Coccurello and Maccarrone, 2018). Also, CB1 antagonism decreases adiposity by a direct effect on white and brown adipocytes, where it reduces lipogenesis (Ma et al., 2018) and increases thermogenesis (Boon et al., 2014), respectively. As known, the anti-obesity drug rimonabant (CB1 inverse agonist) entered in the market in 2006 showing great efficacy in reducing adiposity, leptin, and insulin resistance as well as improving glucose and lipid metabolism (Quarta and Cota, 2020). However, 2 years later, the drug was removed from the market due to relevant side effects as anxiety, depression, and suicidal ideation (Christensen et al., 2007; Van Gaal et al., 2008; Sam et al., 2011). Therefore, there is a huge interest in the development of CB1 antagonism as a pharmacological tool for the treatment of metabolic disorders, with a better safety profile. In this sense, a study was performed in male C57BL/6 N mice undergoing auditory fear conditioning, followed by re-exposure to the tone with TM38837, largely peripherally restricted CB1 antagonist. Authors evaluated fear-promoting consequences of systemic vs. intracerebral injections and showed that TM38837 was at least one order of magnitude less effective in promoting fear responses than rimonabant. Given the equipotency of the two CB1 antagonists with regard to weight loss and metabolic syndrome-like symptoms in rodent obesity models, the results point to a critical dose range in which TM3887 might be beneficial for indications such as obesity and metabolic disorders with limited risk of fear-promoting effects (Micale et al., 2019).

The impact of pharmacological modulation of CB2 on body weight regulation and metabolism is still controversial, but this receptor is involved in the inflammatory status of the WAT. The pharmacological CB2 antagonism improved adiposity and pro-inflammatory state in WAT and liver of obese mice (Deveaux et al., 2009). On the other hand, Rossi et al. (2016) suggested that CB2 activation has anti-obesity effects, since the CB2 blockage increases fat store and reduces browning in human adipocytes. Furthermore, studies have reported a CB2 anti-obesity effect by silencing the activated immune cells in mice adipose tissue (Verty et al., 2015; Notarnicola et al., 2016).

Currently, there are no anti-obesity drugs based on the ECS in the market but pre-clinical studies suggest that CB1 pharmacological modulation using peripherally restrict molecules are promising (Quarta and Cota, 2020). Alternatively, non-pharmacological strategies of weight loss can also modulate the ECS avoiding the side effects in the CNS.

Caloric restriction is well known to be an important strategy for weight loss and improvement of metabolism. Recently, it was demonstrated that these beneficial effects involve a decrease in the circulating levels of AEA in type 2 diabetic patients which was strongly correlated with a decrease of the subcutaneous adipose tissue mass and, possibly, increased content of FAAH in adipose tissue (van Eyk et al., 2018). Low AEA levels may result in attenuation of the ECS tonus in the CNS reducing hungry and in peripheral tissues attenuating lipogenesis and insulin resistance (Bermudez-Siva et al., 2006). In contrast, the investigation of the ECS components in subcutaneous adipose tissue before and after weight loss in obese patients showed no effect on tissue levels of AEA and increased expression of *Faah* mRNA (Bennetzen et al., 2011). In a randomized controlled trial, healthy subjects underwent a 12-week moderate aerobic exercise program for weight loss and showed reduced levels of AEA which was associated with improvement in mood and anger (Belitardo de Oliveira et al., 2019). Polymorphisms of the *Faah* gene have been associated with metabolic benefits after weight loss induced by a 3-month dietary intervention with a high-PUFA diet in obese subjects, such as a decrease in insulin resistance and leptinemia (de Luis et al., 2013), evidencing the important role of the endocannabinoid metabolism in metabolic health. Weight loss in morbid obesity (BMI > 40) is even more challenging and frequently requires a more invasive intervention, such as bariatric surgery. Interestingly, the weight loss and metabolic improvement observed in gastric bypass (RYGB) involve activation of the sympathetic tonus in the gut, which was associated with decreased CB1 and activation of WAT thermogenesis, a phenomenon known as “browning” that increases energy expenditure and resting metabolic rate (Ye et al., 2020). Therefore, the modulation of the peripheral ECS (adipose tissue, gut, and liver) may also represent an important therapeutical target for bodyweight management.

## Endocannabinoids and Inborn Errors of Metabolism

Inborn errors of metabolism (IEM) are genetic diseases caused by qualitative or quantitative deficiency of a specific protein. This protein may be an enzyme, transporter, receptor or else, and can affect different metabolic pathways. At present, more than 1,000 IEM have been described (Ferreira et al., 2019) and the majority significantly impact the quality of life of affected patients.

A few inborn errors of eCB metabolism have been described and can be associated with neurodegeneration (Metzler, 2011). Polyneuropathy, hearing loss, ataxia, retinitis pigmentosa, retinal degeneration and early-onset cataract, as well as cerebellar ataxia and slowly progressive polyneuropathy, are the main findings observed in a disease known as PHARC (Fiskerstrand et al., 2010; Nishiguchi et al., 2014). PHARC is an autosomal recessive disease caused by mutations in the α/β hydrolase domain-12 (ABHD12) gene, leading to increased 2-AG levels (Fiskerstrand et al., 2010).

Fatty acid amide hydrolase deficiency significantly increases AEA sensitivity and endocannabinoid signaling (Cravatt et al., 2001). Individuals presenting mutations in the FAAH gene are more susceptible to drug and/or alcohol abuse (Sipe et al., 2002; Flanagan et al., 2006; Sim et al., 2013). Furthermore, inheritable deletions in the pseudogene FAAHP1, as found in pain sensitivity quantitative trait locus-1 (PAINQTL1), are associated with insensitivity to pain (Habib et al., 2019).

Endocannabinoid system may also be implicated in the pathophysiology of other IEM. For instance, it has been suggested that endocannabinoid metabolism and signaling are impaired in several lysosomal storage diseases (LSD) (Schuchman et al., 2021). Studies using animal models of Niemann-Pick disease type A and B (acid sphingomyelinase deficiency) showed that CB1 receptors are downregulated on the surface of neurons probably due to entrapment of the receptor within lysosomes (Bartoll et al., 2020). Niemann-Pick disease type C is a potentially fatal LSD caused by mutations of NPC1 or NPC2 gene, impairing cholesterol homeostasis (Vanier, 2010). Membrane cholesterol plays a pivotal role in the ECS regulation (Dainese et al., 2010). This neurotransmitter system has been shown to be defective in animal models of Niemann-Pick disease type C (Galles et al., 2018; van Rooden et al., 2018), contributing to the neurodegeneration observed in patients (Oddi et al., 2019). CB2 receptors were shown to be altered in animal models of mucopolysaccharidosis type IIIA and of acid ceramidase deficiency (Farber’s disease) (Bhaumik et al., 1999; Alayoubi et al., 2013). In both diseases the overexpression of CB2 found in many tissues are probably related to the neuroinflammation observed in these animals (Schuchman et al., 2021).

Organic acidurias are IEM characterized by the accumulation of one or more organic acids in tissue and body fluids of patients, with significantly brain function impairment (Wajner, 2019). Glutaric aciduria type I, methylmalonic aciduria and propionic aciduria are organic acidurias in which neurodegeneration is related to different pathophysiological mechanisms including oxidative stress, impairment of bioenergetics, and neurotransmitter systems (Wajner, 2019). *In vitro* experiments showed that WIN55,212-2 protects from neurotoxicity elicited by organic acids accumulating in glutaric aciduria type I, methylmalonic aciduria and propionic aciduria. This protective effect was attributed to a possible ECS imbalance in the pathophysiology of organic acidurias (Colín-González et al., 2015).

## Author Contributions

All authors listed have made a substantial, direct and intellectual contribution to the work, and approved it for publication.

## Conflict of Interest

The authors declare that the research was conducted in the absence of any commercial or financial relationships that could be construed as a potential conflict of interest.

## Publisher’s Note

All claims expressed in this article are solely those of the authors and do not necessarily represent those of their affiliated organizations, or those of the publisher, the editors and the reviewers. Any product that may be evaluated in this article, or claim that may be made by its manufacturer, is not guaranteed or endorsed by the publisher.

## Footnotes

1
https://www.wada-ama.org/en/questions-answers/cannabinoid

## Abbreviations

ALA: alpha linolenic acid
ALEA: alpha-linolenoylethanolamide
AD: Alzheimer’s disease
CBD: cannabidiol
CB1: cannabinoid receptor type 1
CB2: cannabinoid receptor type 2
ECS: endocannabinoid system
CNS: central nervous system
anandamide or AEA: N-arachidonoylethanolamide
2-AG: 2-arachidonoylglycerol
LC-PUFAs: long chain polyunsaturated fatty acids
MAPK: mitogen-activated protein kinase
OEA: O-arachidonoylethanolamide
THC: tetrahydrocannabinol
HP: hemopressin
FAAH: fatty acid amide hydrolase
PNS: peripheral nervous system
GM: gray matter
HPA: hypothalamic-pituitary-adrenal
eCB: endocannabinoid
PEA: palmitoyl-ethanolamide
PD: Parkinson’s disease
DHEA: N-docosahexaenoyl -ethanolamine
EPEA: N-eicosapentaenoyl -ethanolamine
2-DHG: 2-docosahexaenoylglycerol
EPG: 2-eicosapentaenoylglycerol
EEQ-EA: epoxyeicosatetraenoic acid-ethanolamide
EDP-EA: epoxydocosapentaenoic acid-ethanolamide
PA: physical activity
AA: Arachidonic acid.

## References

[B1] Abel E. L. (1975). Cannabis: effects on hunger and thirst. *Behav. Biol.* 15 255–281. 1106391 10.1016/s0091-6773(75)91684-3

[B2] Ahmad R. Goffin K. Van Den Stock J. De Winter F. L. Cleeren E. Bormans G. (2014). In vivo type 1 cannabinoid receptor availability in Alzheimer’s disease. *Eur. Neuropsychopharmacol.* 24 242–250. 24189376 10.1016/j.euroneuro.2013.10.002

[B3] Alayoubi A. M. Wang J. C. Au B. C. Carpentier S. Garcia V. Dworski S. (2013). Systemic ceramide accumulation leads to severe and varied pathological consequences. *EMBO Mol. Med.* 5 827–842. 10.1002/emmm.201202301 23681708 PMC3779446

[B4] Almeida M. M. Dias-Rocha C. P. Reis-Gomes C. F. Wang H. Atella G. C. Cordeiro A. (2019). Maternal high-fat diet impairs leptin signaling and up-regulates type-1 cannabinoid receptor with sex-specific epigenetic changes in the hypothalamus of newborn rats. *Psychoneuroendocrinology* 103 306–315. 10.1016/j.psyneuen.2019.02.004 30776574

[B5] Almeida M. M. Dias-Rocha C. P. Reis-Gomes C. F. Wang H. Cordeiro A. Pazos-Moura C. C. (2020). Maternal high-fat diet up-regulates type-1 cannabinoid receptor with estrogen signaling changes in a sex- and depot- specific manner in white adipose tissue of adult rat offspring. *Eur. J. Nutr*. 60 1313–1326. 32671459 10.1007/s00394-020-02318-w

[B6] Almeida M. M. Dias-Rocha C. P. Souza A. S. Muros M. F. Mendonca L. S. Pazos-Moura C. C. (2017). Perinatal maternal high-fat diet induces early obesity and sex-specific alterations of the endocannabinoid system in white and brown adipose tissue of weanling rat offspring. *Br. J. Nutr.* 118 788–803. 10.1017/S0007114517002884 29110748

[B7] Alpár A. Di Marzo V. Harkany T. (2016). At the tip of an iceberg: prenatal marijuana and its possible relation to neuropsychiatric outcome in the offspring. *Biol. Psychiatry* 79 e33–e45. 10.1016/j.biopsych.2015.09.009 26549491

[B8] Alteba S. Korem N. Akirav I. (2016). Cannabinoids reverse the effects of early stress on neurocognitive performance in adulthood. *Learn. Mem.* 23 349–358. 10.1101/lm.041608.116 27317195 PMC4918780

[B9] Andrade-da-Costa B. L. D. S. Isaac A. R. Augusto R. L. De Souza R. F. Freitas H. R. De Melo Reis R. A. (2019). “Epigenetic effects of omega-3 fatty acids on neurons and astrocytes during brain development and senescence,” in *Omega Fatty Acids in Brain and Neurological Health*, eds Watson R. R. Preedy V. R. (Cambridge, MA: Academic Press), 479–490.

[B10] Arida R. M. Gomes Da Silva S. De Almeida A. A. Cavalheiro E. A. Zavala-Tecuapetla C. Brand S. (2015). Differential effects of exercise on brain opioid receptor binding and activation in rats. *J. Neurochem.* 132 206–217. 25330347 10.1111/jnc.12976

[B11] Ashton C. H. (2001). Pharmacology and effects of cannabis: a brief review. *Br. J. Psychiatry* 178 101–106. 11157422 10.1192/bjp.178.2.101

[B12] Aso E. Palomer E. Juvés S. Maldonado R. Muñoz F. J. Ferrer I. (2012). CB1 agonist ACEA protects neurons and reduces the cognitive impairment of AβPP/PS1 mice. *J. Alzheimers Dis.* 30 439–459. 10.3233/JAD-2012-111862 22451318

[B13] Aso E. Sánchez-Pla A. Vegas-Lozano E. Maldonado R. Ferrer I. (2015). Cannabis-based medicine reduces multiple pathological processes in AβPP/PS1 mice. *J. Alzheimers Dis.* 43 977–991. 10.3233/JAD-141014 25125475

[B14] Assis L. C. Straliotto M. R. Engel D. Hort M. A. Dutra R. C. De Bem A. F. (2014). β-Caryophyllene protects the C6 glioma cells against glutamate-induced excitotoxicity through the Nrf2 pathway. *Neuroscience* 279 220–231. 10.1016/j.neuroscience.2014.08.043 25194788

[B15] Assmann K. E. Adjibade M. Shivappa N. Hébert J. R. Wirth M. D. Touvier M. (2018). The inflammatory potential of the diet at midlife is associated with later healthy aging in French adults. *J. Nutr.* 148 437–444. 10.1093/jn/nxx061 29546305 PMC6251567

[B16] Babson K. A. Sottile J. Morabito D. (2017). Cannabis, cannabinoids, and sleep: a review of the literature. *Curr. Psychiatry Rep.* 19:23. 10.1007/s11920-017-0775-9 28349316

[B17] Bahi A. Al Mansouri S. Al Memari E. Al Ameri M. Nurulain S. M. Ojha S. (2014). β-Caryophyllene, a CB2 receptor agonist produces multiple behavioral changes relevant to anxiety and depression in mice. *Physiol. Behav.* 135 119–124. 10.1016/j.physbeh.2014.06.003 24930711

[B18] Banni S. Di Marzo V. (2010). Effect of dietary fat on endocannabinoids and related mediators: consequences on energy homeostasis, inflammation and mood. *Mol. Nutr. Food Res.* 54 82–92. 10.1002/mnfr.200900516 20013888

[B19] Bara A. Ferland J. N. Rompala G. Szutorisz H. Hurd Y. L. (2021). Cannabis and synaptic reprogramming of the developing brain. *Nat. Rev. Neurosci.* 22 423–438. 10.1038/s41583-021-00465-5 34021274 PMC8445589

[B20] Barna I. Zelena D. Arszovszki A. C. Ledent C. (2004). The role of endogenous cannabinoids in the hypothalamo-pituitary-adrenal axis regulation: in vivo and in vitro studies in CB1 receptor knockout mice. *Life Sci.* 75 2959–2970. 10.1016/j.lfs.2004.06.006 15454346

[B21] Bartoll A. Toledano-Zaragoza A. Casas J. Guzmán M. Schuchman E. H. Ledesma M. D. (2020). Inhibition of fatty acid amide hydrolase prevents pathology in neurovisceral acid sphingomyelinase deficiency by rescuing defective endocannabinoid signaling. *EMBO Mol. Med.* 12:e11776. 10.15252/emmm.201911776 33016621 PMC7645369

[B22] Batetta B. Griinari M. Carta G. Murru E. Ligresti A. Cordeddu L. (2009). Endocannabinoids may mediate the ability of (n-3) fatty acids to reduce ectopic fat and inflammatory mediators in obese Zucker rats. *J. Nutr.* 139 1495–1501. 10.3945/jn.109.104844 19549757

[B23] Battistella G. Fornari E. Annoni J. M. Chtioui H. Dao K. Fabritius M. (2014). Long-term effects of cannabis on brain structure. *Neuropsychopharmacology* 39 2041–2048. 10.1038/npp.2014.67 24633558 PMC4104335

[B24] Bedse G. Romano A. Cianci S. Lavecchia A. M. Lorenzo P. Elphick M. R. (2014). Altered expression of the CB1 cannabinoid receptor in the triple transgenic mouse model of Alzheimer’s disease. *J. Alzheimers Dis.* 40 701–712. 10.3233/JAD-131910 24496074

[B25] Beggiato S. Borelli A. C. Tomasini M. C. Morgano L. Antonelli T. Tanganelli S. (2017). Long-lasting alterations of hippocampal GABAergic neurotransmission in adult rats following perinatal Delta(9)-THC exposure. *Neurobiol. Learn. Mem.* 139 135–143. 10.1016/j.nlm.2016.12.023 28104530

[B26] Behan C. (2020). The benefits of meditation and mindfulness practices during times of crisis such as COVID-19. *Irish J. Psychol. Med.* 37 256–258. 10.1017/ipm.2020.38 32406348 PMC7287297

[B27] Beins E. C. Beiert T. Jenniches I. Hansen J. N. Leidmaa E. Schrickel J. W. (2021). Cannabinoid receptor 1 signalling modulates stress susceptibility and microglial responses to chronic social defeat stress. *Transl. Psychiatry* 11:164. 10.1038/s41398-021-01283-0 33723234 PMC7961142

[B28] Belenguer P. Duarte J. M. N. Schuck P. F. Ferreira G. C. (2019). Mitochondria and the brain: bioenergetics and beyond. *Neurotox Res.* 36 219–238. 31152314 10.1007/s12640-019-00061-7

[B29] Belitardo de Oliveira A. De Mello M. T. Tufik S. Peres M. F. P. (2019). Weight loss and improved mood after aerobic exercise training are linked to lower plasma anandamide in healthy people. *Physiol. Behav.* 201 191–197. 10.1016/j.physbeh.2018.12.018 30578894

[B30] Bénard G. Massa F. Puente N. Lourenço J. Bellocchio L. Soria-Gómez E. (2012). Mitochondrial CB1 receptors regulate neuronal energy metabolism. *Nat. Neurosci.* 15 558–564. 22388959 10.1038/nn.3053

[B31] Benarroch E. (2007). Endocannabinoids in basal ganglia circuits: implications for Parkinson disease. *Neurology* 69 306–309. 17636069 10.1212/01.wnl.0000267407.79757.75

[B32] Benito C. Núñez E. Tolón R. M. Carrier E. J. Rábano A. Hillard C. J. (2003). Cannabinoid CB2 receptors and fatty acid amide hydrolase are selectively overexpressed in neuritic plaque-associated glia in Alzheimer’s disease brains. *J. Neurosci.* 23 11136–11141. 10.1523/JNEUROSCI.23-35-11136.2003 14657172 PMC6741043

[B33] Bennett G. Young E. Butler I. Coe S. (2021). The impact of lockdown during the COVID-19 outbreak on dietary habits in various population groups: a scoping review. *Front. Nutr.* 8:626432. 10.3389/fnut.2021.626432 33748175 PMC7969646

[B34] Bennetzen M. F. Nielsen T. S. Paulsen S. K. Bendix J. Fisker S. Jessen N. (2010). Reduced cannabinoid receptor 1 protein in subcutaneous adipose tissue of obese. *Eur. J. Clin. Invest.* 40 121–126. 20039929 10.1111/j.1365-2362.2009.02231.x

[B35] Bennetzen M. F. Wellner N. Ahmed S. S. Ahmed S. M. Diep T. A. Hansen H. S. (2011). Investigations of the human endocannabinoid system in two subcutaneous adipose tissue depots in lean subjects and in obese subjects before and after weight loss. *Int. J. Obes.* 35 1377–1384. 10.1038/ijo.2011.8 21326208

[B36] Berge K. Piscitelli F. Hoem N. Silvestri C. Meyer I. Banni S. (2013). Chronic treatment with krill powder reduces plasma triglyceride and anandamide levels in mildly obese men. *Lipids Health Dis.* 12:78. 10.1186/1476-511X-12-78 23706001 PMC3680309

[B37] Bermudez-Siva F. J. Serrano A. Diaz-Molina F. J. Sanchez Vera I. Juan-Pico P. Nadal A. (2006). Activation of cannabinoid CB1 receptors induces glucose intolerance in rats. *Eur. J. Pharmacol.* 531 282–284. 16423347 10.1016/j.ejphar.2005.12.016

[B38] Bhaumik M. Muller V. J. Rozaklis T. Johnson L. Dobrenis K. Bhattacharyya R. (1999). A mouse model for mucopolysaccharidosis type III A (Sanfilippo syndrome). *Glycobiology* 9 1389–1396. 10561464 10.1093/glycob/9.12.1389

[B39] Bisogno T. Maccarrone M. (2014). Endocannabinoid signaling and its regulation by nutrients. *Biofactors* 40 373–380. 24753395 10.1002/biof.1167

[B40] Blesching U. (2020). *Your Cannabis CBD:THC Ratio: A Guide to Precision Dosing for Health and Wellness.* Quick Trading Company.

[B41] Boon M. R. Kooijman S. Van Dam A. D. Pelgrom L. R. Berbee J. F. Visseren C. A. (2014). Peripheral cannabinoid 1 receptor blockade activates brown adipose tissue and diminishes dyslipidemia and obesity. *FASEB J.* 28 5361–5375. 10.1096/fj.13-247643 25154875

[B42] Bouret S. G. Draper S. J. Simerly R. B. (2004). Trophic action of leptin on hypothalamic neurons that regulate feeding. *Science* 304 108–110. 15064420 10.1126/science.1095004

[B43] Brenseke B. Prater M. R. Bahamonde J. Gutierrez J. C. (2013). Current thoughts on maternal nutrition and fetal programming of the metabolic syndrome. *J. Pregnancy* 2013:368461. 10.1155/2013/368461 23476780 PMC3586494

[B44] Bridgeman M. B. Abazia D. T. (2017). Medicinal cannabis: history, pharmacology, and implications for the acute care setting. *P & T* 42 180–188. 28250701 PMC5312634

[B45] Broers B. Patà Z. Mina A. Wampfler J. De saussure C. Pautex S. (2019). Prescription of a THC/CBD-based medication to patients with dementia: a pilot study in Geneva. *Med. Cannabis Cannabinoids* 2 56–59. 34676334 10.1159/000498924 PMC8489328

[B46] Brook R. D. Appel L. J. Rubenfire M. Ogedegbe G. Bisognano J. D. Elliott W. J. (2013). Beyond medications and diet: alternative approaches to lowering blood pressure. *Hypertension* 61 1360–1383. 23608661 10.1161/HYP.0b013e318293645f

[B47] Bühlmayer L. Birrer D. Röthlin P. Faude O. Donath L. (2017). Effects of mindfulness practice on performance-relevant parameters and performance outcomes in sports: a meta-analytical review. *Sports Med.* 47 2309–2321. 10.1007/s40279-017-0752-9 28664327

[B48] Burbridge S. Stewart I. Placzek M. (2016). Development of the neuroendocrine hypothalamus. *Compr. Physiol.* 6 623–643. 27065164 10.1002/cphy.c150023

[B49] Burgio E. Lopomo A. Migliore L. (2015). Obesity and diabetes: from genetics to epigenetics. *Mol. Biol. Rep.* 42 799–818. 25253098 10.1007/s11033-014-3751-z

[B50] Cahn B. R. Goodman M. S. Peterson C. T. Maturi R. Mills P. J. (2017). Yoga, meditation and mind-body health: increased BDNF, cortisol awakening response, and altered inflammatory marker expression after a 3-month yoga and meditation retreat. *Front. Hum. Neurosci.* 11:315. 10.3389/fnhum.2017.00315 28694775 PMC5483482

[B51] Calderon-Garciduenas L. Herrera-Soto A. Jury N. Maher B. A. Gonzalez-Maciel A. Reynoso-Robles R. (2020). Reduced repressive epigenetic marks, increased DNA damage and Alzheimer’s disease hallmarks in the brain of humans and mice exposed to particulate urban air pollution. *Environ. Res.* 183:109226. 10.1016/j.envres.2020.109226 32045727

[B52] Camille Melon L. Maguire J. (2016). GABAergic regulation of the HPA and HPG axes and the impact of stress on reproductive function. *J. Steroid Biochem. Mol. Biol.* 160 196–203. 10.1016/j.jsbmb.2015.11.019 26690789 PMC4861672

[B53] Campolongo P. Trezza V. Cassano T. Gaetani S. Morgese M. G. Ubaldi M. (2007). Perinatal exposure to delta-9-tetrahydrocannabinol causes enduring cognitive deficits associated with alteration of cortical gene expression and neurotransmission in rats. *Addict. Biol.* 12 485–495. 10.1111/j.1369-1600.2007.00074.x 17578508

[B54] Campos A. C. Fogaça M. V. Sonego A. B. Guimarães F. S. (2016). Cannabidiol, neuroprotection and neuropsychiatric disorders. *Pharmacol. Res.* 112 119–127. 26845349 10.1016/j.phrs.2016.01.033

[B55] Cao-Lei L. Elgbeili G. Szyf M. Laplante D. P. King S. (2019). Differential genome-wide DNA methylation patterns in childhood obesity. *BMC Res. Notes* 12:174. 10.1186/s13104-019-4189-0 30909978 PMC6434834

[B56] Castelli M. P. Fadda P. Casu A. Spano M. S. Casti A. Fratta W. (2014). Male and female rats differ in brain cannabinoid CB1 receptor density and function and in behavioural traits predisposing to drug addiction: effect of ovarian hormones. *Curr. Pharm. Des.* 20 2100–2113. 10.2174/13816128113199990430 23829370

[B57] Celorrio M. Fernández-Suárez D. Rojo-Bustamante E. Echeverry-Alzate V. Ramírez M. J. Hillard C. J. (2016). Fatty acid amide hydrolase inhibition for the symptomatic relief of Parkinson’s disease. *Brain Behav. Immun.* 57 94–105. 10.1016/j.bbi.2016.06.010 27318096

[B58] Chagas M. H. Zuardi A. W. Tumas V. Pena-Pereira M. A. Sobreira E. T. Bergamaschi M. M. (2014). Effects of cannabidiol in the treatment of patients with Parkinson’s disease: an exploratory double-blind trial. *J. Psychopharmacol.* 28 1088–1098. 10.1177/0269881114550355 25237116

[B59] Chen P. Zhang Q. Zhang H. Gao Y. Zhou Y. Chen Y. (2021). Carnitine palmitoyltransferase 1C reverses cellular senescence of MRC-5 fibroblasts via regulating lipid accumulation and mitochondrial function. *J. Cell. Physiol.* 236 958–970. 10.1002/jcp.29906 32632982

[B60] Cheng D. Spiro A. S. Jenner A. M. Garner B. Karl T. (2014). Long-term cannabidiol treatment prevents the development of social recognition memory deficits in Alzheimer’s disease transgenic mice. *J. Alzheimers Dis.* 42 1383–1396. 10.3233/JAD-140921 25024347

[B61] Cheng Y. Dong Z. Liu S. (2014). β-Caryophyllene ameliorates the Alzheimer-like phenotype in APP/PS1 Mice through CB2 receptor activation and the PPARγ pathway. *Pharmacology* 94 1–12. 10.1159/000362689 25171128

[B62] Chiurchiu V. Lanuti M. Catanzaro G. Fezza F. Rapino C. Maccarrone M. (2014). Detailed characterization of the endocannabinoid system in human macrophages and foam cells, and anti-inflammatory role of type-2 cannabinoid receptor. *Atherosclerosis* 233 55–63. 10.1016/j.atherosclerosis.2013.12.042 24529123

[B63] Choi I. Y. Ju C. Anthony Jalin A. M. Lee D. I. Prather P. L. Kim W. K. (2013). Activation of cannabinoid CB2 receptor-mediated AMPK/CREB pathway reduces cerebral ischemic injury. *Am. J. Pathol.* 182 928–939. 10.1016/j.ajpath.2012.11.024 23414569

[B64] Choi S. Huang B. C. Gamaldo C. E. (2020). Therapeutic uses of cannabis on sleep disorders and related conditions. *J. Clin. Neurophysiol.* 37 39–49. 31895189 10.1097/WNP.0000000000000617

[B65] Christensen R. Kristensen P. K. Bartels E. M. Bliddal H. Astrup A. (2007). Efficacy and safety of the weight-loss drug rimonabant: a meta-analysis of randomised trials. *Lancet* 370 1706–1713. 18022033 10.1016/S0140-6736(07)61721-8

[B66] Coccurello R. Maccarrone M. (2018). Hedonic eating and the “delicious circle”: from lipid-derived mediators to brain dopamine and back. *Front. Neurosci.* 12:271. 10.3389/fnins.2018.00271 29740277 PMC5928395

[B67] Colín-González A. L. Paz-Loyola A. L. Serratos I. N. Seminotti B. Ribeiro C. A. Leipnitz G. (2015). The effect of WIN 55,212-2 suggests a cannabinoid-sensitive component in the early toxicity induced by organic acids accumulating in glutaric acidemia type I and in related disorders of propionate metabolism in rat brain synaptosomes. *Neuroscience* 310 578–588. 10.1016/j.neuroscience.2015.09.043 26431622

[B68] Concannon R. M. Okine B. N. Finn D. P. Dowd E. (2015). Differential upregulation of the cannabinoid CB2 receptor in neurotoxic and inflammation-driven rat models of Parkinson’s disease. *Exp. Neurol.* 269 133–141. 10.1016/j.expneurol.2015.04.007 25895887

[B69] Contrepois K. Wu S. Moneghetti K. J. Hornburg D. Ahadi S. Tsai M.-S. (2020). Molecular choreography of acute exercise. *Cell* 181 1112–1130.e16. 32470399 10.1016/j.cell.2020.04.043 PMC7299174

[B70] Cooper Z. D. Haney M. (2014). Investigation of sex-dependent effects of cannabis in daily cannabis smokers. *Drug Alcohol Depend.* 136 85–91. 10.1016/j.drugalcdep.2013.12.013 24440051 PMC4518446

[B71] Cotrim B. A. Joglar J. Rojas M. J. Del Olmo J. M. Macias-González M. Cuevas M. R. (2012). Unsaturated fatty alcohol derivatives of olive oil phenolic compounds with potential low-density lipoprotein (LDL) antioxidant and antiobesity properties. *J. Agric. Food Chem.* 60 1067–1074. 10.1021/jf203814r 22220510

[B72] Cramer H. Lauche R. Langhorst J. Dobos G. (2013). Yoga for depression: a systematic review and meta-analysis. *Depress. Anxiety* 30 1068–1083. 23922209 10.1002/da.22166

[B73] Cravatt B. F. Demarest K. Patricelli M. P. Bracey M. H. Giang D. K. Martin B. R. (2001). Supersensitivity to anandamide and enhanced endogenous cannabinoid signaling in mice lacking fatty acid amide hydrolase. *Proc. Natl. Acad. Sci. U.S.A.* 98 9371–9376. 10.1073/pnas.161191698 11470906 PMC55427

[B74] Crean R. D. Crane N. A. Mason B. J. (2011). An evidence based review of acute and long-term effects of cannabis use on executive cognitive functions. *J. Addict. Med.* 5 1–8. 21321675 10.1097/ADM.0b013e31820c23fa PMC3037578

[B75] Cristino L. Bisogno T. Di Marzo V. (2020). Cannabinoids and the expanded endocannabinoid system in neurological disorders. *Nat. Rev. Neurol.* 16 9–29. 31831863 10.1038/s41582-019-0284-z

[B76] Cristino L. Busetto G. Imperatore R. Ferrandino I. Palomba L. Silvestri C. (2013). Obesity-driven synaptic remodeling affects endocannabinoid control of orexinergic neurons. *Proc. Natl. Acad. Sci. U.S.A.* 110 E2229–E2238. 10.1073/pnas.1219485110 23630288 PMC3683753

[B77] Crombie K. M. Sartin-Tarm A. Sellnow K. Ahrenholtz R. Lee S. Matalamaki M. (2021). Exercise-induced increases in Anandamide and BDNF during extinction consolidation contribute to reduced threat following reinstatement: preliminary evidence from a randomized controlled trial. *Psychoneuroendocrinology* 132:105355. 10.1016/j.psyneuen.2021.105355 34280820 PMC8487992

[B78] Cruz-Martinez A. M. Tejas-Juarez J. G. Mancilla-Diaz J. M. Floran-Garduno B. Lopez-Alonso V. E. Escartin-Perez R. E. (2018). CB1 receptors in the paraventricular nucleus of the hypothalamus modulate the release of 5-HT and GABA to stimulate food intake in rats. *Eur. Neuropsychopharmacol.* 28 1247–1259. 10.1016/j.euroneuro.2018.08.002 30217553

[B79] Cunha J. M. Carlini E. A. Pereira A. E. Ramos O. L. Pimentel C. Gagliardi R. (1980). Chronic administration of cannabidiol to healthy volunteers and epileptic patients. *Pharmacology* 21 175–185. 7413719 10.1159/000137430

[B80] Cusick S. E. Georgieff M. K. (2016). The role of nutrition in brain development: the golden opportunity of the “first 1000 days”. *J. Pediatr.* 175 16–21. 10.1016/j.jpeds.2016.05.013 27266965 PMC4981537

[B81] Cuttler C. Spradlin A. Cleveland M. J. Craft R. M. (2020). Short- and long-term effects of cannabis on headache and migraine. *J. Pain* 21 722–730. 10.1016/j.jpain.2019.11.001 31715263

[B82] D’Addario C. Micale V. Di Bartolomeo M. Stark T. Pucci M. Sulcova A. (2017). A preliminary study of endocannabinoid system regulation in psychosis: distinct alterations of CNR1 promoter DNA methylation in patients with schizophrenia. *Schizophr. Res.* 188 132–140. 10.1016/j.schres.2017.01.022 28108228

[B83] D’Addario C. Micioni Di Bonaventura M. V. Pucci M. Romano A. Gaetani S. Ciccocioppo R. (2014). Endocannabinoid signaling and food addiction. *Neurosci. Biobehav. Rev.* 47 203–224. 25173635 10.1016/j.neubiorev.2014.08.008

[B84] Dainese E. Oddi S. Maccarrone M. (2010). Interaction of endocannabinoid receptors with biological membranes. *Curr. Med. Chem.* 17 1487–1499. 20166920 10.2174/092986710790980087

[B85] D’Angelo S. Motti M. L. Meccariello R. (2020). ω-3 and ω-6 polyunsaturated fatty acids, obesity and cancer. *Nutrients* 12:2751. 10.3390/nu12092751 32927614 PMC7551151

[B86] Danhauer S. C. Addington E. L. Sohl S. J. Chaoul A. Cohen L. (2017). Review of yoga therapy during cancer treatment. *Support Care Cancer* 25 1357–1372. 28064385 10.1007/s00520-016-3556-9 PMC5777241

[B87] Darlington D. N. Miyamoto M. Keil L. C. Dallman M. F. (1989). Paraventricular stimulation with glutamate elicits bradycardia and pituitary responses. *Am. J. Physiol.* 256 R112–R119. 10.1152/ajpregu.1989.256.1.R112 2563205

[B88] Dauer W. Przedborski S. (2003). Parkinson’s disease: mechanisms and models. *Neuron* 39 889–909. 12971891 10.1016/s0896-6273(03)00568-3

[B89] de Almeida M. M. Dias-Rocha C. P. Reis-Gomes C. F. Wang H. Cordeiro A. Pazos-Moura C. C. (2021). Maternal high-fat diet up-regulates type-1 cannabinoid receptor with estrogen signaling changes in a sex- and depot- specific manner in white adipose tissue of adult rat offspring. *Eur. J. Nutr.* 60 1313–1326. 10.1007/s00394-020-02318-w 32671459

[B90] de Aquino C. C. Leitão R. A. Oliveira Alves L. A. Coelho-Santos V. Guerrant R. L. Ribeiro C. F. (2019). Effect of hypoproteic and high-fat diets on hippocampal blood-brain barrier permeability and oxidative stress. *Front. Nutr.* 5:131. 10.3389/fnut.2018.00131 30687711 PMC6333637

[B91] De Genna N. M. Goldschmidt L. Richardson G. A. Cornelius M. D. Day N. L. (2021). Prenatal exposure to tobacco and cannabis, early cannabis initiation, and daily dual use of combustible cigarettes and cannabis during young adulthood. *Addict. Behav.* 116:106820. 10.1016/j.addbeh.2021.106820 33516042 PMC7953574

[B92] de Luis D. A. Izaola O. Aller R. De La Fuente B. Pacheco D. (2013). Effects of C358A polymorphism of the endocannabinoid degrading enzyme fatty acid amide hydrolase (FAAH) on weight loss, adipocytokines levels, and insulin resistance after a high polyunsaturated fat diet in obese patients. *J. Endocrinol. Invest.* 36 965–969. 10.1007/BF03346760 24445122

[B93] de Oliveira E. Quitete F. T. Bernardino D. N. Guarda D. S. Caramez F. A. H. Soares P. N. (2019). Maternal coconut oil intake on lactation programs for endocannabinoid system dysfunction in adult offspring. *Food Chem. Toxicol.* 130 12–21. 10.1016/j.fct.2019.05.002 31059745

[B94] De Petrocellis L. Cascio M. G. Di Marzo V. (2004). The endocannabinoid system: a general view and latest additions. *Br. J. Pharmacol.* 141 765–774. 10.1038/sj.bjp.0705666 14744801 PMC1574255

[B95] de Salas-Quiroga A. Diaz-Alonso J. Garcia-Rincon D. Remmers F. Vega D. Gomez-Canas M. (2015). Prenatal exposure to cannabinoids evokes long-lasting functional alterations by targeting CB1 receptors on developing cortical neurons. *Proc. Natl. Acad. Sci. U.S.A.* 112 13693–13698. 10.1073/pnas.1514962112 26460022 PMC4640742

[B96] de Salas-Quiroga A. Garcia-Rincon D. Gomez-Dominguez D. Valero M. Simon-Sanchez S. Paraiso-Luna J. (2020). Long-term hippocampal interneuronopathy drives sex-dimorphic spatial memory impairment induced by prenatal THC exposure. *Neuropsychopharmacology* 45 877–886. 10.1038/s41386-020-0621-3 31982904 PMC7075920

[B97] Deahl M. (1991). Cannabis and memory loss. *Br. J. Addict.* 86 249–252. 2025687 10.1111/j.1360-0443.1991.tb01776.x

[B98] Demirakca T. Sartorius A. Ende G. Meyer N. Welzel H. Skopp G. (2011). Diminished gray matter in the hippocampus of cannabis users: possible protective effects of cannabidiol. *Drug Alcohol Depend.* 114 242–245. 10.1016/j.drugalcdep.2010.09.020 21050680

[B99] Deveaux V. Cadoudal T. Ichigotani Y. Teixeira-Clerc F. Louvet A. Manin S. (2009). Cannabinoid CB2 receptor potentiates obesity-associated inflammation, insulin resistance and hepatic steatosis. *PLoS One* 4:e5844. 10.1371/journal.pone.0005844 19513120 PMC2688760

[B100] Devine M. J. Kittler J. T. (2018). Mitochondria at the neuronal presynapse in health and disease. *Nat. Rev. Neurosci.* 19 63–80. 10.1038/nrn.2017.170 29348666

[B101] Di Bartolomeo M. Stark T. Maurel O. M. Iannotti F. A. Kuchar M. Ruda-Kucerova J. (2021). Crosstalk between the transcriptional regulation of dopamine D2 and cannabinoid CB1 receptors in schizophrenia: analyses in patients and in perinatal Δ9-tetrahydrocannabinol-exposed rats. *Pharmacol. Res.* 164:105357. 10.1016/j.phrs.2020.105357 33285233

[B102] Di Francesco A. Falconi A. Di Germanio C. Micioni Di Bonaventura M. V. Costa A. Caramuta S. (2015). Extravirgin olive oil up-regulates CB(1) tumor suppressor gene in human colon cancer cells and in rat colon via epigenetic mechanisms. *J. Nutr. Biochem.* 26 250–258. 10.1016/j.jnutbio.2014.10.013 25533906

[B103] Di Liegro C. M. Schiera G. Proia P. Di Liegro I. (2019). Physical activity and brain health. *Genes* 10:720. 10.3390/genes10090720 PMC6770965 31533339

[B104] Di Marzo V. (1998). ‘Endocannabinoids’ and other fatty acid derivatives with cannabimimetic properties: biochemistry and possible physiopathological relevance. *Biochim. Biophys. Acta* 1392 153–175. 10.1016/s0005-2760(98)00042-3 9630590

[B105] Di Marzo V. Piscitelli F. (2015). The endocannabinoid system and its modulation by phytocannabinoids. *Neurotherapeutics* 12 692–698. 26271952 10.1007/s13311-015-0374-6 PMC4604172

[B106] Dias-Rocha C. P. Almeida M. M. Santana E. M. Costa J. C. B. Franco J. G. Pazos-Moura C. C. (2018). Maternal high-fat diet induces sex-specific endocannabinoid system changes in newborn rats and programs adiposity, energy expenditure and food preference in adulthood. *J. Nutr. Biochem.* 51 56–68. 10.1016/j.jnutbio.2017.09.019 29102876

[B107] Dienel G. A. (2019). Brain glucose metabolism: integration of energetics with function. *Physiol. Rev.* 99 949–1045. 30565508 10.1152/physrev.00062.2017

[B108] Docter S. Khan M. Gohal C. Ravi B. Bhandari M. Gandhi R. (2020). Cannabis use and sport: a systematic review. *Sports Health* 12 189–199. 32023171 10.1177/1941738120901670 PMC7040945

[B109] Dodd G. T. Mancini G. Lutz B. Luckman S. M. (2010). The peptide hemopressin acts through CB1 cannabinoid receptors to reduce food intake in rats and mice. *J. Neurosci.* 30 7369–7376. 10.1523/JNEUROSCI.5455-09.2010 20505104 PMC6632410

[B110] Dragano N. R. Haddad-Tovolli R. Velloso L. A. (2017). Leptin, neuroinflammation and obesity. *Front. Horm. Res.* 48 84–96. 10.1159/000452908 28245454

[B111] Dyall S. C. Mandhair H. K. Fincham R. E. A. Kerr D. M. Roche M. Molina-Holgado F. (2016). Distinctive effects of eicosapentaenoic and docosahexaenoic acids in regulating neural stem cell fate are mediated via endocannabinoid signalling pathways. *Neuropharmacology* 107 387–395. 10.1016/j.neuropharm.2016.03.055 27044662

[B112] Egertová M. Cravatt B. F. Elphick M. R. (2003). Comparative analysis of fatty acid amide hydrolase and cb(1) cannabinoid receptor expression in the mouse brain: evidence of a widespread role for fatty acid amide hydrolase in regulation of endocannabinoid signaling. *Neuroscience* 119 481–496. 10.1016/s0306-4522(03)00145-3 12770562

[B113] El Swefy S. Hasan R. A. Ibrahim A. Mahmoud M. F. (2016). Curcumin and hemopressin treatment attenuates cholestasis-induced liver fibrosis in rats: role of CB1 receptors. *Naunyn Schmiedebergs Arch. Pharmacol.* 389 103–116. 10.1007/s00210-015-1181-7 26475620

[B114] Esposito G. De Filippis D. Carnuccio R. Izzo A. A. Iuvone T. (2006). The marijuana component cannabidiol inhibits beta-amyloid-induced tau protein hyperphosphorylation through Wnt/beta-catenin pathway rescue in PC12 cells. *J. Mol. Med.* 84 253–258. 10.1007/s00109-005-0025-1 16389547

[B115] Facci L. Dal Toso R. Romanello S. Buriani A. Skaper S. D. Leon A. (1995). Mast cells express a peripheral cannabinoid receptor with differential sensitivity to anandamide and palmitoylethanolamide. *Proc. Natl. Acad. Sci. U.S.A.* 92 3376–3380. 10.1073/pnas.92.8.3376 7724569 PMC42169

[B116] Fang R. Li X. (2015). A regular yoga intervention for staff nurse sleep quality and work stress: a randomised controlled trial. *J. Clin. Nurs.* 24 3374–3379. 10.1111/jocn.12983 26478577

[B117] Fattore L. Spano M. S. Altea S. Angius F. Fadda P. Fratta W. (2007). Cannabinoid self-administration in rats: sex differences and the influence of ovarian function. *Br. J. Pharmacol.* 152 795–804. 17891164 10.1038/sj.bjp.0707465 PMC2190022

[B118] Fernández-Suárez D. Celorrio M. Riezu-Boj J. I. Ugarte A. Pacheco R. González H. (2014). Monoacylglycerol lipase inhibitor JZL184 is neuroprotective and alters glial cell phenotype in the chronic MPTP mouse model. *Neurobiol. Aging* 35 2603–2616. 10.1016/j.neurobiolaging.2014.05.021 24973119

[B119] Ferrari D. Chiozzi P. Falzoni S. Dal Susino M. Collo G. Buell G. (1997). ATP-mediated cytotoxicity in microglial cells. *Neuropharmacology* 36 1295–1301. 9364484 10.1016/s0028-3908(97)00137-8

[B120] Ferreira C. R. Van Karnebeek C. D. M. Vockley J. Blau N. (2019). A proposed nosology of inborn errors of metabolism. *Genet. Med.* 21 102–106. 29884839 10.1038/s41436-018-0022-8 PMC6286709

[B121] Ferreira G. C. McKenna M. C. (2017). L-carnitine and acetyl-L-carnitine roles and neuroprotection in developing brain. *Neurochem. Res.* 42 1661–1675. 28508995 10.1007/s11064-017-2288-7 PMC5621476

[B122] Field T. Diego M. Delgado J. Medina L. (2013). Tai chi/yoga reduces prenatal depression, anxiety and sleep disturbances. *Complement. Ther. Clin. Pract.* 19 6–10. 10.1016/j.ctcp.2012.10.001 23337557 PMC3730281

[B123] Figueiredo P. R. Tolomeo S. Steele J. D. Baldacchino A. (2020). Neurocognitive consequences of chronic cannabis use: a systematic review and meta-analysis. *Neurosci. Biobehav. Rev.* 108 358–369. 10.1016/j.neubiorev.2019.10.014 31715191

[B124] Filbey F. M. Aslan S. Calhoun V. D. Spence J. S. Damaraju E. Caprihan A. (2014). Long-term effects of marijuana use on the brain. *Proc. Natl. Acad. Sci. U.S.A.* 111 16913–16918. 25385625 10.1073/pnas.1415297111 PMC4250161

[B125] Fiskerstrand T. H’mida-Ben Brahim D. Johansson S. M’zahem A. Haukanes B. I. Drouot N. (2010). Mutations in ABHD12 cause the neurodegenerative disease PHARC: an inborn error of endocannabinoid metabolism. *Am. J. Hum. Genet.* 87 410–417. 10.1016/j.ajhg.2010.08.002 20797687 PMC2933347

[B126] Flanagan J. M. Gerber A. L. Cadet J. L. Beutler E. Sipe J. C. (2006). The fatty acid amide hydrolase 385 A/A (P129T) variant: haplotype analysis of an ancient missense mutation and validation of risk for drug addiction. *Hum. Genet.* 120 581–588. 10.1007/s00439-006-0250-x 16972078

[B127] Foltin R. W. Brady J. V. Fischman M. W. (1986). Behavioral analysis of marijuana effects on food intake in humans. *Pharmacol. Biochem. Behav.* 25 577–582. 3774823 10.1016/0091-3057(86)90144-9

[B128] Ford C. G. Vowles K. E. Smith B. W. Kinney A. Y. (2020). Mindfulness and meditative movement interventions for men living with cancer: a meta-analysis. *Ann. Behav. Med.* 54 360–373. 10.1093/abm/kaz053 31773148 PMC7168578

[B129] Formolo D. A. Gaspar J. M. Melo H. M. Eichwald T. Zepeda R. J. Latini A. (2019). Deep brain stimulation for obesity: a review and future directions. *Front. Neurosci.* 13:323. 10.3389/fnins.2019.00323 31057350 PMC6482165

[B130] Freitas H. R. Ferreira G. D. C. Trevenzoli I. H. Oliveira K. J. De Melo Reis R. A. (2017). Fatty acids, antioxidants and physical activity in brain aging. *Nutrients* 9:1263. 10.3390/nu9111263 PMC5707735 29156608

[B131] Freitas H. R. Isaac A. R. Malcher-Lopes R. Diaz B. L. Trevenzoli I. H. De Melo Reis R. A. (2018). Polyunsaturated fatty acids and endocannabinoids in health and disease. *Nutr. Neurosci.* 21 695–714. 28686542 10.1080/1028415X.2017.1347373

[B132] Friedman J. M. (2019). Leptin and the endocrine control of energy balance. *Nat. Metab.* 1 754–764. 32694767 10.1038/s42255-019-0095-y

[B133] Fuss J. Steinle J. Bindila L. Auer M. K. Kirchherr H. Lutz B. (2015). A runner’s high depends on cannabinoid receptors in mice. *Proc. Natl. Acad. Sci. U.S.A.* 112 13105–13108. 10.1073/pnas.1514996112 26438875 PMC4620874

[B134] Galles C. Prez G. M. Penkov S. Boland S. Porta E. O. J. Altabe S. G. (2018). Endocannabinoids in *Caenorhabditis elegans* are essential for the mobilization of cholesterol from internal reserves. *Sci. Rep.* 8:6398. 10.1038/s41598-018-24925-8 29686301 PMC5913221

[B135] Gandhi K. Li C. German N. Skobowiat C. Carrillo M. Kallem R. R. (2018). Effect of maternal high-fat diet on key components of the placental and hepatic endocannabinoid system. *Am. J. Physiol. Endocrinol. Metab.* 314 E322–E333. 10.1152/ajpendo.00119.2017 29138223 PMC5966752

[B136] García-Arencibia M. González S. De Lago E. Ramos J. A. Mechoulam R. Fernández-Ruiz J. (2007). Evaluation of the neuroprotective effect of cannabinoids in a rat model of Parkinson’s disease: importance of antioxidant and cannabinoid receptor-independent properties. *Brain Res.* 1134 162–170. 10.1016/j.brainres.2006.11.063 17196181

[B137] Geisler B. P. (2016). Cardiovascular benefits of the Mediterranean diet are driven by stroke reduction and possibly by decreased atrial fibrillation incidence. *Am. J. Med.* 129:e11. 10.1016/j.amjmed.2015.04.046 26703006

[B138] Gertsch J. Leonti M. Raduner S. Racz I. Chen J. Z. Xie X. Q. (2008). Beta-caryophyllene is a dietary cannabinoid. *Proc. Natl. Acad. Sci. U.S.A.* 105 9099–9104. 18574142 10.1073/pnas.0803601105 PMC2449371

[B139] Gertsch J. Pertwee R. G. Di Marzo V. (2010). Phytocannabinoids beyond the cannabis plant – do they exist? *Br. J. Pharmacol.* 160 523–529. 10.1111/j.1476-5381.2010.00745.x 20590562 PMC2931553

[B140] Ghaffari B. D. Kluger B. (2014). Mechanisms for alternative treatments in Parkinson’s disease: acupuncture, tai chi, and other treatments. *Curr. Neurol. Neurosci. Rep.* 14:451. 10.1007/s11910-014-0451-y 24760476

[B141] Gjerstad J. K. Lightman S. L. Spiga F. (2018). Role of glucocorticoid negative feedback in the regulation of HPA axis pulsatility. *Stress* 21 403–416. 29764284 10.1080/10253890.2018.1470238 PMC6220752

[B142] Glass M. Dragunow M. Faull R. L. (2000). The pattern of neurodegeneration in Huntington’s disease: a comparative study of cannabinoid, dopamine, adenosine and GABA(A) receptor alterations in the human basal ganglia in Huntington’s disease. *Neuroscience* 97 505–519. 10828533 10.1016/s0306-4522(00)00008-7

[B143] Gluckman P. D. Hanson M. A. Low F. M. (2019). Evolutionary and developmental mismatches are consequences of adaptive developmental plasticity in humans and have implications for later disease risk. *Philos. Trans. R Soc. Lond. B Biol. Sci.* 374:20180109. 10.1098/rstb.2018.0109 30966891 PMC6460082

[B144] Gomes I. Grushko J. S. Golebiewska U. Hoogendoorn S. Gupta A. Heimann A. S. (2009). Novel endogenous peptide agonists of cannabinoid receptors. *FASEB J.* 23 3020–3029. 19380512 10.1096/fj.09-132142 PMC2735371

[B145] Gomes T. M. Dias Da Silva D. Carmo H. Carvalho F. Silva J. P. (2020). Epigenetics and the endocannabinoid system signaling: an intricate interplay modulating neurodevelopment. *Pharmacol. Res.* 162:105237. 10.1016/j.phrs.2020.105237 33053442

[B146] Grimaldi P. Pucci M. Di Siena S. Di Giacomo D. Pirazzi V. Geremia R. (2012). The faah gene is the first direct target of estrogen in the testis: role of histone demethylase LSD1. *Cell. Mol. Life Sci.* 69 4177–4190. 10.1007/s00018-012-1074-6 22802127 PMC11114663

[B147] Grzywacz A. Barczak W. Chmielowiec J. Chmielowiec K. Suchanecka A. Trybek G. (2020). Contribution of dopamine transporter gene methylation status to cannabis dependency. *Brain Sci.* 10:400. 10.3390/brainsci10060400 32586035 PMC7348832

[B148] Gubellini P. Picconi B. Bari M. Battista N. Calabresi P. Centonze D. (2002). Experimental Parkinsonism alters endocannabinoid degradation: implications for striatal glutamatergic transmission. *J. Neurosci.* 22 6900–6907. 10.1523/JNEUROSCI.22-16-06900.2002 12177188 PMC6757864

[B149] Habib A. M. Okorokov A. L. Hill M. N. Bras J. T. Lee M. C. Li S. (2019). Microdeletion in a FAAH pseudogene identified in a patient with high anandamide concentrations and pain insensitivity. *Br. J. Anaesth.* 123 e249–e253. 10.1016/j.bja.2019.02.019 30929760 PMC6676009

[B150] Haspula D. Clark M. A. (2020). Cannabinoid receptors: an update on cell signaling, pathophysiological roles and therapeutic opportunities in neurological, cardiovascular, and inflammatory diseases. *Int. J. Mol. Sci.* 21:7693. 10.3390/ijms21207693 33080916 PMC7590033

[B151] Hebert-Chatelain E. Desprez T. Serrat R. Bellocchio L. Soria-Gomez E. Busquets-Garcia A. (2016). A cannabinoid link between mitochondria and memory. *Nature* 539 555–559. 27828947 10.1038/nature20127

[B152] Heimann A. S. Dale C. S. Guimarães F. S. Reis R. A. M. Navon A. Shmuelov M. A. (2020a). Hemopressin as a breakthrough for the cannabinoid field. *Neuropharmacology* 183:108406. 10.1016/j.neuropharm.2020.108406 33212113 PMC8609950

[B153] Heimann A. S. Giardini A. C. Sant’anna M. B. Dos Santos N. B. Gewehr M. C. F. Munhoz C. D. (2020b). NFKF is a synthetic fragment derived from rat hemopressin that protects mice from neurodegeneration. *Neurosci. Lett.* 721:134765. 10.1016/j.neulet.2020.134765 32004656

[B154] Herkenham M. Lynn A. B. Johnson M. R. Melvin L. S. De Costa B. R. Rice K. C. (1991). Characterization and localization of cannabinoid receptors in rat brain: a quantitative in vitro autoradiographic study. *J. Neurosci.* 11 563–583. 10.1523/JNEUROSCI.11-02-00563.1991 1992016 PMC6575215

[B155] Herkenham M. Lynn A. B. Little M. D. Johnson M. R. Melvin L. S. De Costa B. R. (1990). Cannabinoid receptor localization in brain. *Proc. Natl. Acad. Sci. U.S.A.* 87 1932–1936. 2308954 10.1073/pnas.87.5.1932 PMC53598

[B156] Herman J. P. Tasker J. G. Ziegler D. R. Cullinan W. E. (2002). Local circuit regulation of paraventricular nucleus stress integration: glutamate-GABA connections. *Pharmacol. Biochem. Behav.* 71 457–468. 10.1016/s0091-3057(01)00681-5 11830180

[B157] Hernandez-Avila C. A. Rounsaville B. J. Kranzler H. R. (2004). Opioid-, cannabis- and alcohol-dependent women show more rapid progression to substance abuse treatment. *Drug Alcohol Depend.* 74 265–272. 10.1016/j.drugalcdep.2004.02.001 15194204

[B158] Heyman E. Gamelin F. X. Goekint M. Piscitelli F. Roelands B. Leclair E. (2012). Intense exercise increases circulating endocannabinoid and BDNF levels in humans–possible implications for reward and depression. *Psychoneuroendocrinology* 37 844–851. 10.1016/j.psyneuen.2011.09.017 22029953

[B159] Hill M. N. Titterness A. K. Morrish A. C. Carrier E. J. Lee T. T. Gil-Mohapel J. (2010). Endogenous cannabinoid signaling is required for voluntary exercise-induced enhancement of progenitor cell proliferation in the hippocampus. *Hippocampus* 20 513–523. 10.1002/hipo.20647 19489006 PMC2847038

[B160] Holland J. (2010). *The Pot Book: A Complete Guide to Cannabis.* Rochester, VT: Inner Traditions/Bear.

[B161] Horváth B. Mukhopadhyay P. Kechrid M. Patel V. Tanchian G. Wink D. A. (2012). β-Caryophyllene ameliorates cisplatin-induced nephrotoxicity in a cannabinoid 2 receptor-dependent manner. *Free Radic. Biol. Med.* 52 1325–1333. 10.1016/j.freeradbiomed.2012.01.014 22326488 PMC3312970

[B162] Hurley M. J. Mash D. C. Jenner P. (2003). Expression of cannabinoid CB1 receptor mRNA in basal ganglia of normal and parkinsonian human brain. *J. Neural Transm.* 110 1279–1288. 10.1007/s00702-003-0033-7 14628192

[B163] Ibrahim M. M. (2010). Subcutaneous and visceral adipose tissue: structural and functional differences. *Obes. Rev.* 11 11–18. 19656312 10.1111/j.1467-789X.2009.00623.x

[B164] Irving A. Abdulrazzaq G. Chan S. L. F. Penman J. Harvey J. Alexander S. P. H. (2017). Cannabinoid receptor-related orphan G protein-coupled receptors. *Adv. Pharmacol.* 80 223–247. 28826536 10.1016/bs.apha.2017.04.004

[B165] Isaac A. R. De Velasco P. C. Fraga K. Y. D. Tavares-Do-Carmo M. D. G. Campos R. M. P. Iannotti F. A. (2021). Maternal omega-3 intake differentially affects the endocannabinoid system in the progeny’s neocortex and hippocampus: impact on synaptic markers: n-3 maternal diet sway progeny’s synaptic markers. *J. Nutr. Biochem.* 96:108782. 10.1016/j.jnutbio.2021.108782 34038760

[B166] Jacobs T. L. Epel E. S. Lin J. Blackburn E. H. Wolkowitz O. M. Bridwell D. A. (2011). Intensive meditation training, immune cell telomerase activity, and psychological mediators. *Psychoneuroendocrinology* 36 664–681. 10.1016/j.psyneuen.2010.09.010 21035949

[B167] Jager G. Kahn R. S. Van Den Brink W. Van Ree J. M. Ramsey N. F. (2006). Long-term effects of frequent cannabis use on working memory and attention: an fMRI study. *Psychopharmacology* 185 358–368. 10.1007/s00213-005-0298-7 16521034

[B168] Jannuzzi L. B. Pereira-Acacio A. Ferreira B. S. N. Silva-Pereira D. Veloso-Santos J. P. M. Alves-Bezerra D. S. (2021). Undernutrition – thirty years of the regional basic diet: the legacy of Naíde Teodósio in different fields of knowledge. *Nutr. Neurosci.* 1–22. 10.1080/1028415X.2021.1915631 33871318

[B169] Javed H. Azimullah S. Haque M. E. Ojha S. K. (2016). Cannabinoid type 2 (CB2) receptors activation protects against oxidative stress and neuroinflammation associated dopaminergic neurodegeneration in rotenone model of Parkinson’s disease. *Front. Neurosci.* 10:321. 10.3389/fnins.2016.00321 27531971 PMC4969295

[B170] Jimenez-Blasco D. Busquets-Garcia A. Hebert-Chatelain E. Serrat R. Vicente-Gutierrez C. Ioannidou C. (2020). Glucose metabolism links astroglial mitochondria to cannabinoid effects. *Nature* 583 603–608. 10.1038/s41586-020-2470-y 32641832

[B171] Jin K. Simpkins J. W. Ji X. Leis M. Stambler I. (2014). The critical need to promote research of aging and aging-related diseases to improve health and longevity of the elderly population. *Aging Dis.* 6 1–5. 10.14336/AD.2014.1210 25657847 PMC4306469

[B172] Jindal V. Gupta S. Das R. (2013). Molecular mechanisms of meditation. *Mol. Neurobiol.* 48 808–811. 23737355 10.1007/s12035-013-8468-9

[B173] Jones A. M. Carter H. (2000). The effect of endurance training on parameters of aerobic fitness. *Sports Med.* 29 373–386. 10870864 10.2165/00007256-200029060-00001

[B174] Jones P. J. Lin L. Gillingham L. G. Yang H. Omar J. M. (2014). Modulation of plasma N-acylethanolamine levels and physiological parameters by dietary fatty acid composition in humans. *J. Lipid Res.* 55 2655–2664. 10.1194/jlr.P051235 25262934 PMC4242457

[B175] Jonsson J. Renault K. M. Garcia-Calzon S. Perfilyev A. Estampador A. C. Norgaard K. (2021). Lifestyle intervention in pregnant women with obesity impacts cord blood DNA methylation which associates with body composition in the offspring. *Diabetes* 70 854–866. 10.2337/db20-0487 33431374 PMC7980200

[B176] Joss-Moore L. A. Lane R. H. Albertine K. H. (2015). Epigenetic contributions to the developmental origins of adult lung disease. *Biochem Cell Biol* 93 119–127. 25493710 10.1139/bcb-2014-0093 PMC5683896

[B177] Kano M. Ohno-Shosaku T. Hashimotodani Y. Uchigashima M. Watanabe M. (2009). Endocannabinoid-mediated control of synaptic transmission. *Physiol Rev* 89 309–380. 19126760 10.1152/physrev.00019.2008

[B178] Katona I. Freund T. F. (2008). Endocannabinoid signaling as a synaptic circuit breaker in neurological disease. *Nat Med* 14 923–930. 18776886 10.1038/nm.f.1869

[B179] Kaur R. Ambwani S. R. Singh S. (2016). Endocannabinoid system: a multi-facet therapeutic target. *Curr. Clin. Pharmacol.* 11 110–117. 10.2174/1574884711666160418105339 27086601

[B180] Kelly T. Yang W. Chen C. S. Reynolds K. He J. (2008). Global burden of obesity in 2005 and projections to 2030. *Int. J. Obes.* 32 1431–1437. 10.1038/ijo.2008.102 18607383

[B181] Khalsa D. S. Newberg A. B. (2021). Spiritual fitness: a new dimension in Alzheimer’s disease prevention. *J. Alzheimers Dis.* 80 505–519. 10.3233/JAD-201433 33554917 PMC8075383

[B182] King-Himmelreich T. S. Schramm S. Wolters M. C. Schmetzer J. Moser C. V. Knothe C. (2016). The impact of endurance exercise on global and AMPK gene-specific DNA methylation. *Biochem. Biophys. Res. Commun.* 474 284–290. 10.1016/j.bbrc.2016.04.078 27103439

[B183] Kirkham T. C. Williams C. M. (2001). Endogenous cannabinoids and appetite. *Nutr. Res. Rev.* 14 65–86. 19087417 10.1079/NRR200118

[B184] Klauke A. L. Racz I. Pradier B. Markert A. Zimmer A. M. Gertsch J. (2014). The cannabinoid CB2 receptor-selective phytocannabinoid beta-caryophyllene exerts analgesic effects in mouse models of inflammatory and neuropathic pain. *Eur. Neuropsychopharmacol.* 24 608–620. 24210682 10.1016/j.euroneuro.2013.10.008

[B185] Koch M. Varela L. Kim J. G. Kim J. D. Hernández-Nuño F. Simonds S. E. (2015). Hypothalamic POMC neurons promote cannabinoid-induced feeding. *Nature* 519 45–50. 10.1038/nature14260 25707796 PMC4496586

[B186] Koppel J. Vingtdeux V. Marambaud P. D’abramo C. Jimenez H. Stauber M. (2014). CB2 receptor deficiency increases amyloid pathology and alters tau processing in a transgenic mouse model of Alzheimer’s disease. *Mol. Med.* 20 29–36. 24722782 10.2119/molmed.2013.00140.revised PMC3951462

[B187] Korte G. Dreiseitel A. Schreier P. Oehme A. Locher S. Geiger S. (2010). Tea catechins’ affinity for human cannabinoid receptors. *Phytomedicine* 17 19–22. 10.1016/j.phymed.2009.10.001 19897346

[B188] Kucerova J. Tabiova K. Drago F. Micale V. (2014). Therapeutic potential of cannabinoids in schizophrenia. *Recent Pat. CNS Drug Discov.* 9 13–25. 24605939 10.2174/1574889809666140307115532

[B189] Kwok J. Y. Y. Kwan J. C. Y. Auyeung M. Mok V. C. T. Lau C. K. Y. Choi K. C. (2019). Effects of mindfulness yoga vs stretching and resistance training exercises on anxiety and depression for people with Parkinson disease: a randomized clinical trial. *JAMA Neurol.* 76 755–763. 10.1001/jamaneurol.2019.0534 30958514 PMC6583059

[B190] Labbé K. Murley A. Nunnari J. (2014). Determinants and functions of mitochondrial behavior. *Annu. Rev. Cell Dev. Biol.* 30 357–391. 25288115 10.1146/annurev-cellbio-101011-155756

[B191] Labra V. C. Santibáñez C. A. Gajardo-Gómez R. Díaz E. F. Gómez G. I. Orellana J. A. (2018). The neuroglial dialog between cannabinoids and hemichannels. *Front. Mol. Neurosci.* 11:79. 10.3389/fnmol.2018.00079 29662436 PMC5890195

[B192] Lafourcade M. Larrieu T. Mato S. Duffaud A. Sepers M. Matias I. (2011). Nutritional omega-3 deficiency abolishes endocannabinoid-mediated neuronal functions. *Nat. Neurosci.* 14 345–350. 10.1038/nn.2736 21278728

[B193] LaFrance E. M. Glodosky N. C. Bonn-Miller M. Cuttler C. (2020). Short and long-term effects of cannabis on symptoms of post-traumatic stress disorder. *J. Affect. Disord.* 274 298–304. 10.1016/j.jad.2020.05.132 32469819

[B194] Lahesmaa M. Eriksson O. Gnad T. Oikonen V. Bucci M. Hirvonen J. (2018). Cannabinoid type 1 receptors are upregulated during acute activation of brown adipose tissue. *Diabetes* 67 1226–1236. 29650773 10.2337/db17-1366

[B195] Larasati Y. A. Yoneda-Kato N. Nakamae I. Yokoyama T. Meiyanto E. Kato J. Y. (2018). Curcumin targets multiple enzymes involved in the ROS metabolic pathway to suppress tumor cell growth. *Sci. Rep.* 8:2039. 10.1038/s41598-018-20179-6 29391517 PMC5794879

[B196] Larrieu T. Madore C. Joffre C. Layé S. (2012). Nutritional n-3 polyunsaturated fatty acids deficiency alters cannabinoid receptor signaling pathway in the brain and associated anxiety-like behavior in mice. *J. Physiol. Biochem.* 68 671–681. 10.1007/s13105-012-0179-6 22707188

[B197] Lastres-Becker I. Molina-Holgado F. Ramos J. A. Mechoulam R. Fernández-Ruiz J. (2005). Cannabinoids provide neuroprotection against 6-hydroxydopamine toxicity in vivo and in vitro: relevance to Parkinson’s disease. *Neurobiol. Dis.* 19 96–107. 10.1016/j.nbd.2004.11.009 15837565

[B198] Laurikainen H. Tuominen L. Tikka M. Merisaari H. Armio R. L. Sormunen E. (2019). Sex difference in brain CB1 receptor availability in man. *Neuroimage* 184 834–842. 30296558 10.1016/j.neuroimage.2018.10.013

[B199] Lee J. H. Agacinski G. Williams J. H. Wilcock G. K. Esiri M. M. Francis P. T. (2010). Intact cannabinoid CB1 receptors in the Alzheimer’s disease cortex. *Neurochem. Int.* 57 985–989. 10.1016/j.neuint.2010.10.010 21034788

[B200] Lee J. Wolfgang M. J. (2012). Metabolomic profiling reveals a role for CPT1c in neuronal oxidative metabolism. *BMC Biochem.* 13:23. 10.1186/1471-2091-13-23 23098614 PMC3523003

[B201] Lee K. S. Asgar J. Zhang Y. Chung M. K. Ro J. Y. (2013). The role of androgen receptor in transcriptional modulation of cannabinoid receptor type 1 gene in rat trigeminal ganglia. *Neuroscience* 254 395–403. 10.1016/j.neuroscience.2013.09.014 24055403 PMC3870904

[B202] Li M. D. Vera N. B. Yang Y. Zhang B. Ni W. Ziso-Qejvanaj E. (2018). Adipocyte OGT governs diet-induced hyperphagia and obesity. *Nat. Commun.* 9:5103. 10.1038/s41467-018-07461-x 30504766 PMC6269424

[B203] Ligresti A. De Petrocellis L. Di Marzo V. (2016). From phytocannabinoids to cannabinoid receptors and endocannabinoids: pleiotropic physiological and pathological roles through complex pharmacology. *Physiol. Rev.* 96 1593–1659. 10.1152/physrev.00002.2016 27630175

[B204] Lillycrop K. A. Burdge G. C. (2015). Maternal diet as a modifier of offspring epigenetics. *J. Dev. Orig. Health Dis.* 6 88–95. 25857738 10.1017/S2040174415000124

[B205] Lim H. K. Lee H. R. Do S. H. (2015). Stimulation of cannabinoid receptors by using *Rubus coreanus* extracts to control osteoporosis in aged male rats. *Aging Male* 18 124–132. 10.3109/13685538.2014.949661 25136745

[B206] Liu M. W. Su M. X. Wang Y. H. Wei W. Qin L. F. Liu X. (2014). Effect of melilotus extract on lung injury by upregulating the expression of cannabinoid CB2 receptors in septic rats. *BMC Complement. Altern. Med.* 14:94. 10.1186/1472-6882-14-94 24612782 PMC3995869

[B207] Llorente-Berzal A. Terzian A. L. Di Marzo V. Micale V. Viveros M. P. Wotjak C. T. (2015). 2-AG promotes the expression of conditioned fear via cannabinoid receptor type 1 on GABAergic neurons. *Psychopharmacology* 232 2811–2825. 10.1007/s00213-015-3917-y 25814137

[B208] Lomazzo E. Konig F. Abassi L. Jelinek R. Lutz B. (2017). Chronic stress leads to epigenetic dysregulation in the neuropeptide-Y and cannabinoid CB1 receptor genes in the mouse cingulate cortex. *Neuropharmacology* 113 301–313. 10.1016/j.neuropharm.2016.10.008 27737789

[B209] Lopez-Gallardo M. Lopez-Rodriguez A. B. Llorente-Berzal A. Rotllant D. Mackie K. Armario A. (2012). Maternal deprivation and adolescent cannabinoid exposure impact hippocampal astrocytes, CB1 receptors and brain-derived neurotrophic factor in a sexually dimorphic fashion. *Neuroscience* 204 90–103. 10.1016/j.neuroscience.2011.09.063 22001306 PMC3659815

[B210] Lotan I. Treves T. A. Roditi Y. Djaldetti R. (2014). Cannabis (medical marijuana) treatment for motor and non-motor symptoms of Parkinson disease: an open-label observational study. *Clin. Neuropharmacol.* 37 41–44. 10.1097/WNF.0000000000000016 24614667

[B211] Ma H. Zhang G. Mou C. Fu X. Chen Y. (2018). Peripheral CB1 receptor neutral antagonist, AM6545, ameliorates hypometabolic obesity and improves adipokine secretion in monosodium glutamate induced obese mice. *Front. Pharmacol.* 9:156. 10.3389/fphar.2018.00156 29615900 PMC5869198

[B212] Maccarrone M. Bab I. Biro T. Cabral G. A. Dey S. K. Di Marzo V. (2015). Endocannabinoid signaling at the periphery: 50 years after THC. *Trends Pharmacol. Sci.* 36 277–296. 10.1016/j.tips.2015.02.008 25796370 PMC4420685

[B213] Maia J. Almada M. Silva A. Correia-Da-Silva G. Teixeira N. Sa S. I. (2017). The endocannabinoid system expression in the female reproductive tract is modulated by estrogen. *J. Steroid Biochem. Mol. Biol.* 174 40–47. 28743542 10.1016/j.jsbmb.2017.07.023

[B214] Maia J. Midao L. Cunha S. C. Almada M. Fonseca B. M. Braga J. (2019). Effects of cannabis tetrahydrocannabinol on endocannabinoid homeostasis in human placenta. *Arch. Toxicol.* 93 649–658. 30659320 10.1007/s00204-019-02389-7

[B215] Mallipeddi S. Janero D. R. Zvonok N. Makriyannis A. (2017). Functional selectivity at G-protein coupled receptors: advancing cannabinoid receptors as drug targets. *Biochem. Pharmacol.* 128 1–11. 10.1016/j.bcp.2016.11.014 27890725 PMC5470118

[B216] Mancino S. Burokas A. Gutierrez-Cuesta J. Gutierrez-Martos M. Martin-Garcia E. Pucci M. (2015). Epigenetic and proteomic expression changes promoted by eating addictive-like behavior. *Neuropsychopharmacology* 40 2788–2800. 10.1038/npp.2015.129 25944409 PMC4864655

[B217] Manuel I. González De San Román E. Giralt M. T. Ferrer I. Rodríguez-Puertas R. (2014). Type-1 cannabinoid receptor activity during Alzheimer’s disease progression. *J. Alzheimers Dis.* 42 761–766. 24946872 10.3233/JAD-140492

[B218] Marco E. M. Echeverry-Alzate V. Lopez-Moreno J. A. Gine E. Penasco S. Viveros M. P. (2014). Consequences of early life stress on the expression of endocannabinoid-related genes in the rat brain. *Behav. Pharmacol.* 25 547–556. 10.1097/FBP.0000000000000068 25083571

[B219] Marsicano G. Lutz B. (1999). Expression of the cannabinoid receptor CB1 in distinct neuronal subpopulations in the adult mouse forebrain. *Eur. J. Neurosci.* 11 4213–4225. 10594647 10.1046/j.1460-9568.1999.00847.x

[B220] Mazier W. Saucisse N. Gatta-Cherifi B. Cota D. (2015). The endocannabinoid system: pivotal orchestrator of obesity and metabolic disease. *Trends Endocrinol. Metab.* 26 524–537. 10.1016/j.tem.2015.07.007 26412154

[B221] Mazzola C. Micale V. Drago F. (2003). Amnesia induced by beta-amyloid fragments is counteracted by cannabinoid CB1 receptor blockade. *Eur. J. Pharmacol.* 477 219–225. 10.1016/j.ejphar.2003.08.026 14522360

[B222] McDougle D. R. Watson J. E. Abdeen A. A. Adili R. Caputo M. P. Krapf J. E. (2017). Anti-inflammatory ω-3 endocannabinoid epoxides. *Proc. Natl. Acad. Sci. U.S.A.* 114 E6034–E6043. 28687674 10.1073/pnas.1610325114 PMC5544256

[B223] McGavin J. J. Cochkanoff N. L. Poole E. I. Crosby K. M. (2019). 2-arachidonylglycerol interacts with nitric oxide in the dorsomedial hypothalamus to increase food intake and body weight in young male rats. *Neurosci. Lett.* 698 27–32. 10.1016/j.neulet.2019.01.008 30615975

[B224] McKenna M. C. Scafidi S. Robertson C. L. (2015). Metabolic alterations in developing brain after injury: knowns and unknowns. *Neurochem. Res.* 40 2527–2543. 26148530 10.1007/s11064-015-1600-7 PMC4961252

[B225] McKenna M. C. Schuck P. F. Ferreira G. C. (2019). Fundamentals of CNS energy metabolism and alterations in lysosomal storage diseases. *J. Neurochem.* 148 590–599. 10.1111/jnc.14577 30144055

[B226] Meccariello R. Santoro A. D’angelo S. Morrone R. Fasano S. Viggiano A. (2020). The epigenetics of the endocannabinoid system. *Int. J. Mol. Sci.* 21:1113. 10.3390/ijms21031113 PMC7037698 32046164

[B227] Mechoulam R. Gaoni Y. (1965). Hashish. IV. The isolation and structure of cannabinolic cannabidiolic and cannabigerolic acids. *Tetrahedron* 21 1223–1229. 10.1016/0040-4020(65)80064-3 5879350

[B228] Mechoulam R. Parker L. A. (2013). The endocannabinoid system and the brain. *Annu. Rev. Psychol.* 64 21–47. 22804774 10.1146/annurev-psych-113011-143739

[B229] Meslier V. Laiola M. Roager H. M. De Filippis F. Roume H. Quinquis B. (2020). Mediterranean diet intervention in overweight and obese subjects lowers plasma cholesterol and causes changes in the gut microbiome and metabolome independently of energy intake. *Gut* 69 1258–1268. 10.1136/gutjnl-2019-320438 32075887 PMC7306983

[B230] Metzler M. (2011). Disturbances in endocannabinoid metabolism causes autosomal recessive neurodegeneration. *Clin. Genet.* 79 221–222. 10.1111/j.1399-0004.2010.01611.x 21199494

[B231] Micale V. Drago F. (2018). Endocannabinoid system, stress and HPA axis. *Eur. J. Pharmacol.* 834 230–239. 30036537 10.1016/j.ejphar.2018.07.039

[B232] Micale V. Di Marzo V. Sulcova A. Wotjak C. T. Drago F. (2013). Endocannabinoid system and mood disorders: priming a target for new therapies. *Pharmacol. Ther.* 138 18–37. 10.1016/j.pharmthera.2012.12.002 23261685

[B233] Micale V. Drago F. Noerregaard P. K. Elling C. E. Wotjak C. T. (2019). The cannabinoid CB1 antagonist TM38837 with limited penetrance to the brain shows reduced fear-promoting effects in mice. *Front. Pharmacol.* 10:207. 10.3389/fphar.2019.00207 30949045 PMC6435594

[B234] Micale V. Mazzola C. Drago F. (2007). Endocannabinoids and neurodegenerative diseases. *Pharmacol. Res.* 56 382–392. 17950616 10.1016/j.phrs.2007.09.008

[B235] Micale V. Stepan J. Jurik A. Pamplona F. A. Marsch R. Drago F. (2017). Extinction of avoidance behavior by safety learning depends on endocannabinoid signaling in the hippocampus. *J. Psychiatr. Res.* 90 46–59. 10.1016/j.jpsychires.2017.02.002 28222356

[B236] Micale V. Tabiova K. Kucerova J. Drago F. (2015). “Role of the endocannabinoid system in depression: from preclinical to clinical evidence,” in *Cannabinoid Modulation of Emotion, Memory, and Motivation*, eds Campolongo P. Fattore L. (New York, NY: Springer New York), 97–129.

[B237] Min Y. Ma X. Sankaran K. Ru Y. Chen L. Baiocchi M. (2019). Sex-specific association between gut microbiome and fat distribution. *Nat. Commun.* 10:2408. 10.1038/s41467-019-10440-5 PMC6546740 31160598

[B238] Miranda R. A. De Almeida M. M. Rocha C. De Brito Fassarella L. De Souza L. L. Souza A. F. P. (2018). Maternal high-fat diet consumption induces sex-dependent alterations of the endocannabinoid system and redox homeostasis in liver of adult rat offspring. *Sci. Rep.* 8:14751. 10.1038/s41598-018-32906-0 30282988 PMC6170403

[B239] Morena M. Patel S. Bains J. S. Hill M. N. (2016). Neurobiological interactions between stress and the endocannabinoid system. *Neuropsychopharmacology* 41 80–102. 26068727 10.1038/npp.2015.166 PMC4677118

[B240] Mulder J. Zilberter M. Pasquaré S. J. Alpár A. Schulte G. Ferreira S. G. (2011). Molecular reorganization of endocannabinoid signalling in Alzheimer’s disease. *Brain* 134 1041–1060. 10.1093/brain/awr046 21459826 PMC3069704

[B241] Muller C. Morales P. Reggio P. H. (2018). Cannabinoid ligands targeting TRP channels. *Front. Mol. Neurosci.* 11:487. 10.3389/fnmol.2018.00487 30697147 PMC6340993

[B242] Muniyappa R. Sable S. Ouwerkerk R. Mari A. Gharib A. M. Walter M. (2013). Metabolic effects of chronic cannabis smoking. *Diabetes Care* 36 2415–2422. 23530011 10.2337/dc12-2303 PMC3714514

[B243] Murphy S. K. Itchon-Ramos N. Visco Z. Huang Z. Grenier C. Schrott R. (2018). Cannabinoid exposure and altered DNA methylation in rat and human sperm. *Epigenetics* 13 1208–1221. 30521419 10.1080/15592294.2018.1554521 PMC6986792

[B244] Muzik O. Diwadkar V. A. (2019). Hierarchical control systems for the regulation of physiological homeostasis and affect: can their interactions modulate mood and anhedonia? *Neurosci. Biobehav. Rev.* 105 251–261. 10.1016/j.neubiorev.2019.08.015 31442518

[B245] Nashed M. G. Hardy D. B. Laviolette S. R. (2020). Prenatal cannabinoid exposure: emerging evidence of physiological and neuropsychiatric abnormalities. *Front. Psychiatry* 11:624275. 10.3389/fpsyt.2020.624275 33519564 PMC7841012

[B246] Natale B. V. Gustin K. N. Lee K. Holloway A. C. Laviolette S. R. Natale D. R. C. (2020). Delta9-tetrahydrocannabinol exposure during rat pregnancy leads to symmetrical fetal growth restriction and labyrinth-specific vascular defects in the placenta. *Sci. Rep.* 10:544. 10.1038/s41598-019-57318-6 31953475 PMC6969028

[B247] Niquet J. Seo D. W. Wasterlain C. G. (2006). Mitochondrial pathways of neuronal necrosis. *Biochem. Soc. Trans.* 34 1347–1351. 17073816 10.1042/BST0341347

[B248] Nishiguchi K. M. Avila-Fernandez A. Van Huet R. A. Corton M. Pérez-Carro R. Martín-Garrido E. (2014). Exome sequencing extends the phenotypic spectrum for ABHD12 mutations: from syndromic to nonsyndromic retinal degeneration. *Ophthalmology* 121 1620–1627. 10.1016/j.ophtha.2014.02.008 24697911

[B249] Notarnicola M. Tutino V. Tafaro A. Bianco G. Guglielmi E. Caruso M. G. (2016). Dietary olive oil induces cannabinoid CB2 receptor expression in adipose tissue of Apc(Min/+) transgenic mice. *Nutr. Healthy Aging* 4 73–80. 10.3233/NHA-160008 28035344 PMC5166557

[B250] O’Callaghan R. M. Ohle R. Kelly ÁM. (2007). The effects of forced exercise on hippocampal plasticity in the rat: a comparison of LTP, spatial- and non-spatial learning. *Behav. Brain Res.* 176 362–366. 10.1016/j.bbr.2006.10.018 17113656

[B251] Oddi S. Caporali P. Dragotto J. Totaro A. Maiolati M. Scipioni L. (2019). The endocannabinoid system is affected by cholesterol dyshomeostasis: insights from a murine model of Niemann Pick type C disease. *Neurobiol. Dis.* 130:104531. 10.1016/j.nbd.2019.104531 31302243

[B252] Oliveira C. D. C. Castor M. Castor C. Costa ÁF. Ferreira R. C. M. Silva J. F. D. (2019). Evidence for the involvement of opioid and cannabinoid systems in the peripheral antinociception mediated by resveratrol. *Toxicol. Appl. Pharmacol.* 369 30–38. 10.1016/j.taap.2019.02.004 30763598

[B253] Oliver E. E. Hughes E. K. Puckett M. K. Chen R. Lowther W. T. Howlett A. C. (2020). Cannabinoid receptor interacting protein 1a (CRIP1a) in health and disease. *Biomolecules* 10:1609. 10.3390/biom10121609 33261012 PMC7761089

[B254] Otrubova K. Ezzili C. Boger D. L. (2011). The discovery and development of inhibitors of fatty acid amide hydrolase (FAAH). *Bioorg. Med. Chem. Lett.* 21 4674–4685. 21764305 10.1016/j.bmcl.2011.06.096 PMC3146581

[B255] Pacher P. Bátkai S. Kunos G. (2006). The endocannabinoid system as an emerging target of pharmacotherapy. *Pharmacol. Rev.* 58 389–462. 16968947 10.1124/pr.58.3.2 PMC2241751

[B256] Pagotto U. Marsicano G. Cota D. Lutz B. Pasquali R. (2006). The emerging role of the endocannabinoid system in endocrine regulation and energy balance. *Endocr. Rev.* 27 73–100. 16306385 10.1210/er.2005-0009

[B257] Palu A. K. Kim A. H. West B. J. Deng S. Jensen J. White L. (2008). The effects of *Morinda citrifolia* L. (noni) on the immune system: its molecular mechanisms of action. *J. Ethnopharmacol.* 115 502–506. 10.1016/j.jep.2007.10.023 18063495

[B258] Pan J. P. Zhang H. Q. Wei W. Guo Y. F. Na X. Cao X. H. (2011). Some subtypes of endocannabinoid/endovanilloid receptors mediate docosahexaenoic acid-induced enhanced spatial memory in rats. *Brain Res.* 1412 18–27. 10.1016/j.brainres.2011.07.015 21803345

[B259] Park H. J. Bailey L. B. Shade D. C. Hausman D. B. Hohos N. M. Meagher R. B. (2017). Distinctions in gene-specific changes in DNA methylation in response to folic acid supplementation between women with normal weight and obesity. *Obes. Res. Clin. Pract.* 11 665–676. 10.1016/j.orcp.2017.06.004 28733112

[B260] Pertwee R. G. (2010). Receptors and channels targeted by synthetic cannabinoid receptor agonists and antagonists. *Curr. Med. Chem.* 17 1360–1381. 10.2174/092986710790980050 20166927 PMC3013229

[B261] Petrosino S. Di Marzo V. (2010). FAAH and MAGL inhibitors: therapeutic opportunities from regulating endocannabinoid levels. *Curr. Opin. Investig. Drugs* 11 51–62. 20047159

[B262] Petrovich G. D. Holland P. C. Gallagher M. (2005). Amygdalar and prefrontal pathways to the lateral hypothalamus are activated by a learned cue that stimulates eating. *J. Neurosci.* 25 8295–8302. 10.1523/JNEUROSCI.2480-05.2005 16148237 PMC6725549

[B263] Pihlaja R. Takkinen J. Eskola O. Vasara J. López-Picón F. R. Haaparanta-Solin M. (2015). Monoacylglycerol lipase inhibitor JZL184 reduces neuroinflammatory response in APdE9 mice and in adult mouse glial cells. *J. Neuroinflammation* 12:81. 10.1186/s12974-015-0305-9 25927213 PMC4416350

[B264] Pisani A. Centonze D. Bernardi G. Calabresi P. (2005). Striatal synaptic plasticity: implications for motor learning and Parkinson’s disease. *Mov. Disord.* 20 395–402. 15719415 10.1002/mds.20394

[B265] Pistis M. O’Sullivan S. E. (2017). The role of nuclear hormone receptors in cannabinoid function. *Adv. Pharmacol.* 80 291–328. 28826538 10.1016/bs.apha.2017.03.008

[B266] Pozo M. Claret M. (2018). Hypothalamic control of systemic glucose homeostasis: the pancreas connection. *Trends Endocrinol. Metab.* 29 581–594. 10.1016/j.tem.2018.05.001 29866501

[B267] Price D. A. Martinez A. A. Seillier A. Koek W. Acosta Y. Fernandez E. (2009). WIN55,212-2, a cannabinoid receptor agonist, protects against nigrostriatal cell loss in the 1-methyl-4-phenyl-1,2,3,6-tetrahydropyridine mouse model of Parkinson’s disease. *Eur. J. Neurosci.* 29 2177–2186. 19490092 10.1111/j.1460-9568.2009.06764.x PMC2755595

[B268] Price N. Van Der Leij F. Jackson V. Corstorphine C. Thomson R. Sorensen A. (2002). A novel brain-expressed protein related to carnitine palmitoyltransferase I. *Genomics* 80 433–442. 10.1006/geno.2002.6845 12376098

[B269] Priestley R. Glass M. Kendall D. (2017). Functional selectivity at cannabinoid receptors. *Adv. Pharmacol.* 80 207–221. 28826535 10.1016/bs.apha.2017.03.005

[B270] Prini P. Zamberletti E. Manenti C. Gabaglio M. Parolaro D. Rubino T. (2020). Neurobiological mechanisms underlying cannabis-induced memory impairment. *Eur. Neuropsychopharmacol.* 36 181–190. 10.1016/j.euroneuro.2020.02.002 32139186

[B271] Proto M. C. Gazzerro P. Di Croce L. Santoro A. Malfitano A. M. Pisanti S. (2012). Interaction of endocannabinoid system and steroid hormones in the control of colon cancer cell growth. *J. Cell. Physiol.* 227 250–258. 10.1002/jcp.22727 21412772

[B272] Quarta C. Cota D. (2020). Anti-obesity therapy with peripheral CB1 blockers: from promise to safe(?) practice. *Int. J. Obes.* 44 2179–2193. 10.1038/s41366-020-0577-8 32317751

[B273] Quezada S. M. Cross R. K. (2019). Cannabis and turmeric as complementary treatments for IBD and other digestive diseases. *Curr. Gastroenterol. Rep.* 21:2. 10.1007/s11894-019-0670-0 30635796

[B274] Radd-Vagenas S. Duffy S. L. Naismith S. L. Brew B. J. Flood V. M. Fiatarone Singh M. A. (2018). Effect of the Mediterranean diet on cognition and brain morphology and function: a systematic review of randomized controlled trials. *Am. J. Clin. Nutr.* 107 389–404. 10.1093/ajcn/nqx070 29566197

[B275] Ramer R. Schwarz R. Hinz B. (2019). Modulation of the endocannabinoid system as a potential anticancer strategy. *Front. Pharmacol.* 10:430. 10.3389/fphar.2019.00430 31143113 PMC6520667

[B276] Ramírez B. G. Blázquez C. Gómez Del Pulgar T. Guzmán M. De Ceballos M. L. (2005). Prevention of Alzheimer’s disease pathology by cannabinoids: neuroprotection mediated by blockade of microglial activation. *J. Neurosci.* 25 1904–1913. 10.1523/JNEUROSCI.4540-04.2005 15728830 PMC6726060

[B277] Ramirez-Lopez M. T. Arco R. Decara J. Vazquez M. Noemi Blanco R. Alen F. (2016a). Exposure to a highly caloric palatable diet during the perinatal period affects the expression of the endogenous cannabinoid system in the brain, liver and adipose tissue of adult rat offspring. *PLoS One* 11:e0165432. 10.1371/journal.pone.0165432 27806128 PMC5091916

[B278] Ramirez-Lopez M. T. Arco R. Decara J. Vazquez M. Rivera P. Blanco R. N. (2016b). Long-term effects of prenatal exposure to undernutrition on cannabinoid receptor-related behaviors: sex and tissue-specific alterations in the mRNA expression of cannabinoid receptors and lipid metabolic regulators. *Front. Behav. Neurosci.* 10:241. 10.3389/fnbeh.2016.00241 28082878 PMC5187359

[B279] Ramirez-Lopez M. T. Vazquez M. Bindila L. Lomazzo E. Hofmann C. Blanco R. N. (2015). Exposure to a highly caloric palatable diet during pregestational and gestational periods affects hypothalamic and hippocampal endocannabinoid levels at birth and induces adiposity and anxiety-like behaviors in male rat offspring. *Front. Behav. Neurosci.* 9:339. 10.3389/fnbeh.2015.00339 26778987 PMC4701936

[B280] Rauschert S. Melton P. E. Burdge G. Craig J. M. Godfrey K. M. Holbrook J. D. (2019). Maternal smoking during pregnancy induces persistent epigenetic changes into adolescence, independent of postnatal smoke exposure and is associated with cardiometabolic risk. *Front. Genet.* 10:770. 10.3389/fgene.2019.00770 31616461 PMC6764289

[B281] Regehr W. G. Carey M. R. Best A. R. (2009). Activity-dependent regulation of synapses by retrograde messengers. *Neuron* 63 154–170. 19640475 10.1016/j.neuron.2009.06.021 PMC3251517

[B282] Ren M. Tang Z. Wu X. Spengler R. Jiang H. Yang Y. (2019). The origins of cannabis smoking: chemical residue evidence from the first millennium BCE in the Pamirs. *Sci. Adv.* 5:eaaw1391. 10.1126/sciadv.aaw1391 31206023 PMC6561734

[B283] Richardson K. A. Hester A. K. Mclemore G. L. (2016). Prenatal cannabis exposure – the “first hit” to the endocannabinoid system. *Neurotoxicol. Teratol.* 58 5–14. 27567698 10.1016/j.ntt.2016.08.003

[B284] Ritter S. L. Hall R. A. (2009). Fine-tuning of GPCR activity by receptor-interacting proteins. *Nat. Rev. Mol. Cell Biol.* 10 819–830. 19935667 10.1038/nrm2803 PMC2825052

[B285] Rivera P. Guerra-Cantera S. Vargas A. Diaz F. Garcia-Ubeda R. Tovar R. (2020). Maternal hypercaloric diet affects factors involved in lipid metabolism and the endogenous cannabinoid systems in the hypothalamus of adult offspring: sex-specific response of astrocytes to palmitic acid and anandamide. *Nutr. Neurosci.* 1–14. 10.1080/1028415X.2020.1821519 32954972

[B286] Rodrigues R. J. Tome A. R. Cunha R. A. (2015). ATP as a multi-target danger signal in the brain. *Front. Neurosci.* 9:148. 10.3389/fnins.2015.00148 25972780 PMC4412015

[B287] Rogers J. P. Chesney E. Oliver D. Pollak T. A. Mcguire P. Fusar-Poli P. (2020). Psychiatric and neuropsychiatric presentations associated with severe coronavirus infections: a systematic review and meta-analysis with comparison to the COVID-19 pandemic. *Lancet Psychiatry* 7 611–627. 10.1016/S2215-0366(20)30203-0 32437679 PMC7234781

[B288] Roh E. Song D. K. Kim M. S. (2016). Emerging role of the brain in the homeostatic regulation of energy and glucose metabolism. *Exp. Mol. Med.* 48:e216. 10.1038/emm.2016.4 PMC4892882 26964832

[B289] Rohde K. Keller M. La Cour Poulsen L. Bluher M. Kovacs P. Bottcher Y. (2019). Genetics and epigenetics in obesity. *Metabolism* 92 37–50. 30399374 10.1016/j.metabol.2018.10.007

[B290] Romano-Lopez A. Mendez-Diaz M. Garcia F. G. Regalado-Santiago C. Ruiz-Contreras A. E. Prospero-Garcia O. (2016). Maternal separation and early stress cause long-lasting effects on dopaminergic and endocannabinergic systems and alters dendritic morphology in the nucleus accumbens and frontal cortex in rats. *Dev. Neurobiol.* 76 819–831. 10.1002/dneu.22361 26539755

[B291] Rossi F. Bellini G. Luongo L. Manzo I. Tolone S. Tortora C. (2016). Cannabinoid receptor 2 as antiobesity target: inflammation, fat storage, and browning modulation. *J. Clin. Endocrinol. Metab.* 101 3469–3478. 10.1210/jc.2015-4381 27294325

[B292] Rossmeisl M. Jilkova Z. M. Kuda O. Jelenik T. Medrikova D. Stankova B. (2012). Metabolic effects of n-3 PUFA as phospholipids are superior to triglycerides in mice fed a high-fat diet: possible role of endocannabinoids. *PLoS One* 7:e38834. 10.1371/journal.pone.0038834 22701720 PMC3372498

[B293] Rotter A. Bayerlein K. Hansbauer M. Weiland J. Sperling W. Kornhuber J. (2013). CB1 and CB2 receptor expression and promoter methylation in patients with cannabis dependence. *Eur. Addict. Res.* 19 13–20. 10.1159/000338642 22948261

[B294] Rozenfeld R. (2011). Type I cannabinoid receptor trafficking: all roads lead to lysosome. *Traffic* 12 12–18. 10.1111/j.1600-0854.2010.01130.x 21040297

[B295] Rozenfeld R. Devi L. A. (2008). Regulation of CB1 cannabinoid receptor trafficking by the adaptor protein AP-3. *FASEB J.* 22 2311–2322. 18267983 10.1096/fj.07-102731 PMC3127579

[B296] Ruhl T. Karthaus N. Kim B. S. Beier J. P. (2020). The endocannabinoid receptors CB1 and CB2 affect the regenerative potential of adipose tissue MSCs. *Exp. Cell Res.* 389:111881. 10.1016/j.yexcr.2020.111881 32006556

[B297] Russo E. B. Guy G. W. Robson P. J. (2007). Cannabis, pain, and sleep: lessons from therapeutic clinical trials of Sativex, a cannabis-based medicine. *Chem. Biodivers.* 4 1729–1743. 10.1002/cbdv.200790150 17712817

[B298] Sadhasivam S. Alankar S. Maturi R. Vishnubhotla R. V. Mudigonda M. Pawale D. (2020). Inner engineering practices and advanced 4-day isha yoga retreat are associated with cannabimimetic effects with increased endocannabinoids and short-term and sustained improvement in mental health: a prospective observational study of meditators. *Evid. Based Complement. Alternat. Med.* 2020:8438272. 10.1155/2020/8438272 32595741 PMC7293737

[B299] Saeed S. A. Cunningham K. Bloch R. M. (2019). Depression and anxiety disorders: benefits of exercise, yoga, and meditation. *Am. Fam. Physician* 99 620–627. 31083878

[B300] Sagar K. A. Dahlgren M. K. Lambros A. M. Smith R. T. El-Abboud C. Gruber S. A. (2021). An observational, longitudinal study of cognition in medical cannabis patients over the course of 12 months of treatment: preliminary results. *J. Int. Neuropsychol. Soc.* 27 648–660. 10.1017/S1355617721000114 34261553

[B301] Sam A. H. Salem V. Ghatei M. A. (2011). Rimonabant: from RIO to Ban. *J. Obes.* 2011:432607. 10.1155/2011/432607 21773005 PMC3136184

[B302] Santos N. A. Martins N. M. Sisti F. M. Fernandes L. S. Ferreira R. S. Queiroz R. H. (2015). The neuroprotection of cannabidiol against MPP?-induced toxicity in PC12 cells involves trkA receptors, upregulation of axonal and synaptic proteins, neuritogenesis, and might be relevant to Parkinson’s disease. *Toxicol. In Vitro* 30(1 Pt B) 231–240. 10.1016/j.tiv.2015.11.004 26556726

[B303] Sarzani R. Bordicchia M. Marcucci P. Bedetta S. Santini S. Giovagnoli A. (2009). Altered pattern of cannabinoid type 1 receptor expression in adipose tissue of dysmetabolic and overweight patients. *Metabolism* 58 361–367. 10.1016/j.metabol.2008.10.009 19217452

[B304] Scherma M. Dessi C. Muntoni A. L. Lecca S. Satta V. Luchicchi A. (2016). Adolescent delta(9)-tetrahydrocannabinol exposure alters WIN55,212-2 self-administration in adult rats. *Neuropsychopharmacology* 41 1416–1426. 10.1038/npp.2015.295 26388146 PMC4793126

[B305] Schonke M. Martinez-Tellez B. Rensen P. C. (2020). Role of the endocannabinoid system in the regulation of the skeletal muscle response to exercise. *Curr. Opin. Pharmacol.* 52 52–60. 10.1016/j.coph.2020.05.003 32619926

[B306] Schuchman E. H. Ledesma M. D. Simonaro C. M. (2021). New paradigms for the treatment of lysosomal storage diseases: targeting the endocannabinoid system as a therapeutic strategy. *Orphanet J. Rare Dis.* 16:151. 10.1186/s13023-021-01779-4 33766102 PMC7992818

[B307] Schwartz R. H. Gruenewald P. J. Klitzner M. Fedio P. (1989). Short-term memory impairment in cannabis-dependent adolescents. *Am. J. Dis. Child* 143 1214–1219. 10.1001/archpedi.1989.02150220110030 2801665

[B308] Scotter E. L. Abood M. E. Glass M. (2010). The endocannabinoid system as a target for the treatment of neurodegenerative disease. *Br. J. Pharmacol.* 160 480–498. 20590559 10.1111/j.1476-5381.2010.00735.x PMC2931550

[B309] Scuderi C. Bronzuoli M. R. Facchinetti R. Pace L. Ferraro L. Broad K. D. (2018). Ultramicronized palmitoylethanolamide rescues learning and memory impairments in a triple transgenic mouse model of Alzheimer’s disease by exerting anti-inflammatory and neuroprotective effects. *Transl. Psychiatry* 8:32. 10.1038/s41398-017-0076-4 29382825 PMC5802581

[B310] Selkoe D. J. (2011). Alzheimer’s disease. *Cold Spring Harb. Perspect. Biol.* 3:a004457. 10.1101/cshperspect.a004457 PMC3119915 21576255

[B311] Sharma C. Sadek B. Goyal S. N. Sinha S. Kamal M. A. Ojha S. (2015). Small molecules from nature targeting G-protein coupled cannabinoid receptors: potential leads for drug discovery and development. *Evid. Based Complement. Alternat. Med.* 2015:238482. 10.1155/2015/238482 26664449 PMC4664820

[B312] Sharon H. Maron-Katz A. Ben Simon E. Flusser Y. Hendler T. Tarrasch R. (2016). Mindfulness meditation modulates pain through endogenous opioids. *Am. J. Med.* 129 755–758. 27039954 10.1016/j.amjmed.2016.03.002

[B313] Shelef A. Barak Y. Berger U. Paleacu D. Tadger S. Plopsky I. (2016). Safety and efficacy of medical cannabis oil for behavioral and psychological symptoms of dementia: an-open label, add-on, pilot study. *J. Alzheimers Dis.* 51 15–19. 10.3233/JAD-150915 26757043

[B314] Shohet A. Khlebtovsky A. Roizen N. Roditi Y. Djaldetti R. (2017). Effect of medical cannabis on thermal quantitative measurements of pain in patients with Parkinson’s disease. *Eur. J. Pain* 21 486–493. 10.1002/ejp.942 27723182

[B315] Sidibeh C. O. Pereira M. J. Lau Borjesson J. Kamble P. G. Skrtic S. Katsogiannos P. (2017). Role of cannabinoid receptor 1 in human adipose tissue for lipolysis regulation and insulin resistance. *Endocrine* 55 839–852. 27858284 10.1007/s12020-016-1172-6 PMC5316391

[B316] Sierra A. Y. Gratacós E. Carrasco P. Clotet J. Ureña J. Serra D. (2008). CPT1c is localized in endoplasmic reticulum of neurons and has carnitine palmitoyltransferase activity. *J. Biol. Chem.* 283 6878–6885. 18192268 10.1074/jbc.M707965200

[B317] Sim M. S. Hatim A. Reynolds G. P. Mohamed Z. (2013). Association of a functional FAAH polymorphism with methamphetamine-induced symptoms and dependence in a Malaysian population. *Pharmacogenomics* 14 505–514. 10.2217/pgs.13.25 23556448

[B318] Simopoulos A. P. (2016). An increase in the omega-6/omega-3 fatty acid ratio increases the risk for obesity. *Nutrients* 8:128. 10.3390/nu8030128 PMC4808858 26950145

[B319] Simopoulos A. P. (2020). Omega-6 and omega-3 fatty acids: endocannabinoids, genetics and obesity?. *OCL* 27:7.

[B320] Sipe J. C. Chiang K. Gerber A. L. Beutler E. Cravatt B. F. (2002). A missense mutation in human fatty acid amide hydrolase associated with problem drug use. *Proc. Natl. Acad. Sci. U.S.A.* 99 8394–8399. 12060782 10.1073/pnas.082235799 PMC123078

[B321] Soares P. N. Miranda R. A. Peixoto T. C. Caramez F. A. H. Guarda D. S. Manhaes A. C. (2019). Cigarette smoke during lactation in rat female progeny: late effects on endocannabinoid and dopaminergic systems. *Life Sci.* 232:116575. 10.1016/j.lfs.2019.116575 31211999

[B322] Solowij N. Michie P. T. (2007). Cannabis and cognitive dysfunction: parallels with endophenotypes of schizophrenia? *J. Psychiatry Neurosci.* 32 30–52. 17245472 PMC1764544

[B323] Soya H. Nakamura T. Deocaris C. C. Kimpara A. Iimura M. Fujikawa T. (2007). BDNF induction with mild exercise in the rat hippocampus. *Biochem. Biophys. Res. Commun.* 358 961–967. 17524360 10.1016/j.bbrc.2007.04.173

[B324] Stang J. Huffman L. G. (2016). Position of the academy of nutrition and dietetics: obesity, reproduction, and pregnancy outcomes. *J. Acad. Nutr. Diet* 116 677–691. 10.1016/j.jand.2016.01.008 27017177

[B325] Stark T. Di Bartolomeo M. Di Marco R. Drazanova E. Platania C. B. M. Iannotti F. A. (2020). Altered dopamine D3 receptor gene expression in MAM model of schizophrenia is reversed by peripubertal cannabidiol treatment. *Biochem. Pharmacol.* 177:114004. 10.1016/j.bcp.2020.114004 32360362

[B326] Stark T. Ruda-Kucerova J. Iannotti F. A. D’addario C. Di Marco R. Pekarik V. (2019). Peripubertal cannabidiol treatment rescues behavioral and neurochemical abnormalities in the MAM model of schizophrenia. *Neuropharmacology* 146 212–221. 10.1016/j.neuropharm.2018.11.035 30496751

[B327] Stringer R. L. Laufer B. I. Kleiber M. L. Singh S. M. (2013). Reduced expression of brain cannabinoid receptor 1 (Cnr1) is coupled with an increased complementary micro-RNA (miR-26b) in a mouse model of fetal alcohol spectrum disorders. *Clin. Epigenetics* 5:14. 10.1186/1868-7083-5-14 23915435 PMC3751098

[B328] Stringfield S. J. Torregrossa M. M. (2021). Intravenous self-administration of delta-9-THC in adolescent rats produces long-lasting alterations in behavior and receptor protein expression. *Psychopharmacology* 238 305–319. 10.1007/s00213-020-05684-9 33111197 PMC7796919

[B329] Styrczewska M. Kulma A. Ratajczak K. Amarowicz R. Szopa J. (2012). Cannabinoid-like anti-inflammatory compounds from flax fiber. *Cell. Mol. Biol. Lett.* 17 479–499. 10.2478/s11658-012-0023-6 22706678 PMC6275574

[B330] Suijun W. Zhen Y. Ying G. Yanfang W. (2014). A role for trans-caryophyllene in the moderation of insulin secretion. *Biochem. Biophys. Res. Commun.* 444 451–454. 10.1016/j.bbrc.2013.11.136 24486541

[B331] Szutorisz H. Hurd Y. L. (2016). Epigenetic effects of cannabis exposure. *Biol. Psychiatry* 79 586–594. 26546076 10.1016/j.biopsych.2015.09.014 PMC4789113

[B332] Tagliamonte S. Laiola M. Ferracane R. Vitale M. Gallo M. A. Meslier V. (2021). Mediterranean diet consumption affects the endocannabinoid system in overweight and obese subjects: possible links with gut microbiome, insulin resistance and inflammation. *Eur. J. Nutr*. 60 3703–3716. 10.1007/s00394-021-02538-8 33763720 PMC8437855

[B333] Tao R. Li C. Jaffe A. E. Shin J. H. Deep-Soboslay A. Yamin R. (2020). Cannabinoid receptor CNR1 expression and DNA methylation in human prefrontal cortex, hippocampus and caudate in brain development and schizophrenia. *Transl. Psychiatry* 10:158. 10.1038/s41398-020-0832-8 32433545 PMC7237456

[B334] Terzian A. L. Drago F. Wotjak C. T. Micale V. (2011). The dopamine and cannabinoid interaction in the modulation of emotions and cognition: assessing the role of cannabinoid CB1 receptor in neurons expressing dopamine D1 receptors. *Front. Behav. Neurosci.* 5:49. 10.3389/fnbeh.2011.00049 21887137 PMC3156975

[B335] Terzian A. L. Micale V. Wotjak C. T. (2014). Cannabinoid receptor type 1 receptors on GABAergic vs. Glutamatergic neurons differentially gate sex-dependent social interest in mice. *Eur. J. Neurosci.* 40 2293–2298. 10.1111/ejn.12561 24698342

[B336] Thanos P. K. Ramalhete R. C. Michaelides M. Piyis Y. K. Wang G. J. Volkow N. D. (2008). Leptin receptor deficiency is associated with upregulation of cannabinoid 1 receptors in limbic brain regions. *Synapse* 62 637–642. 10.1002/syn.20531 18563836 PMC2659017

[B337] Thomazeau A. Bosch-Bouju C. Manzoni O. Layé S. (2017). Nutritional n-3 PUFA deficiency abolishes endocannabinoid gating of hippocampal long-term potentiation. *Cereb. Cortex* 27 2571–2579. 10.1093/cercor/bhw052 26946127

[B338] Thornton C. Hagberg H. (2015). Role of mitochondria in apoptotic and necroptotic cell death in the developing brain. *Clin. Chim. Acta* 451 35–38. 10.1016/j.cca.2015.01.026 25661091 PMC4661434

[B339] Thorsdottir I. Tomasson H. Gunnarsdottir I. Gisladottir E. Kiely M. Parra M. D. (2007). Randomized trial of weight-loss-diets for young adults varying in fish and fish oil content. *Int. J. Obes.* 31 1560–1566. 10.1038/sj.ijo.0803643 17502874

[B340] Toniolo E. F. Maique E. T. Ferreira W. A. Jr. Heimann A. S. Ferro E. S. Ramos-Ortolaza D. L. (2014). Hemopressin, an inverse agonist of cannabinoid receptors, inhibits neuropathic pain in rats. *Peptides* 56 125–131. 10.1016/j.peptides.2014.03.016 24703998 PMC4112957

[B341] Toth M. J. Tchernof A. Sites C. K. Poehlman E. T. (2000). Effect of menopausal status on body composition and abdominal fat distribution. *Int. J. Obes. Relat. Metab. Disord.* 24 226–231. 10702775 10.1038/sj.ijo.0801118

[B342] Tremblay B. L. Guenard F. Rudkowska I. Lemieux S. Couture P. Vohl M. C. (2017). Epigenetic changes in blood leukocytes following an omega-3 fatty acid supplementation. *Clin. Epigenetics* 9:43. 10.1186/s13148-017-0345-3 PMC5405524 28450971

[B343] Tripathi R. K. P. (2020). A perspective review on fatty acid amide hydrolase (FAAH) inhibitors as potential therapeutic agents. *Eur. J. Med. Chem.* 188:111953. 10.1016/j.ejmech.2019.111953 31945644

[B344] Tsuboi H. Sakakibara H. Matsunaga M. Tatsumi A. Yamakawa-Kobayashi K. Yoshida N. (2020). Omega-3 eicosapentaenoic acid is related to happiness and a sense of fulfillment-a study among female nursing workers. *Nutrients* 12:3462. 10.3390/nu12113462 33187281 PMC7696953

[B345] Tsuyama S. Oikawa D. Tsuji Y. Akimoto Y. Jikuya H. Furuse M. (2009). Dietary conjugated linoleic acid modifies the brain endocannabinoid system in mice. *Nutr. Neurosci.* 12 155–159. 10.1179/147683009X423373 19622239

[B346] Tysnes O. B. Storstein A. (2017). Epidemiology of Parkinson’s disease. *J. Neural Transm.* 124 901–905. 28150045 10.1007/s00702-017-1686-y

[B347] Ueda Y. Miyagawa N. Wakitani K. (2007). Involvement of cannabinoid CB2 receptors in the IgE-mediated triphasic cutaneous reaction in mice. *Life Sci.* 80 414–419. 10.1016/j.lfs.2006.09.026 17055000

[B348] van den Elsen G. A. Ahmed A. I. Verkes R. J. Kramers C. Feuth T. Rosenberg P. B. (2015). Tetrahydrocannabinol for neuropsychiatric symptoms in dementia: a randomized controlled trial. *Neurology* 84 2338–2346. 25972490 10.1212/WNL.0000000000001675 PMC4464746

[B349] van der Stelt M. Mazzola C. Esposito G. Matias I. Petrosino S. De Filippis D. (2006). Endocannabinoids and beta-amyloid-induced neurotoxicity in vivo: effect of pharmacological elevation of endocannabinoid levels. *Cell. Mol. Life Sci.* 63 1410–1424. 10.1007/s00018-006-6037-3 16732431 PMC11136405

[B350] van Eyk H. J. Van Schinkel L. D. Kantae V. Dronkers C. E. A. Westenberg J. J. M. De Roos A. (2018). Caloric restriction lowers endocannabinoid tonus and improves cardiac function in type 2 diabetes. *Nutr. Diabetes* 8:6. 10.1038/s41387-017-0016-7 29343706 PMC5851430

[B351] Van Gaal L. Pi-Sunyer X. Despres J. P. Mccarthy C. Scheen A. (2008). Efficacy and safety of rimonabant for improvement of multiple cardiometabolic risk factors in overweight/obese patients: pooled 1-year data from the Rimonabant in Obesity (RIO) program. *Diabetes Care* 31 Suppl. 2 S229–S240. 10.2337/dc08-s258 18227491

[B352] van Rooden E. J. Van Esbroeck A. C. M. Baggelaar M. P. Deng H. Florea B. I. Marques A. R. A. (2018). Chemical proteomic analysis of serine hydrolase activity in niemann-pick type C mouse brain. *Front. Neurosci.* 12:440. 10.3389/fnins.2018.00440 30018533 PMC6037894

[B353] Vanier M. T. (2010). Niemann-Pick disease type C. *Orphanet J. Rare Dis.* 5:16. 10.1186/1750-1172-5-16 PMC2902432 20525256

[B354] Velayudhan L. Van Diepen E. Marudkar M. Hands O. Suribhatla S. Prettyman R. (2014). Therapeutic potential of cannabinoids in neurodegenerative disorders: a selective review. *Curr. Pharm. Des.* 20 2218–2230. 23829360 10.2174/13816128113199990434

[B355] Velusami C. C. Agarwal A. Mookambeswaran V. (2013). Effect of Nelumbo nucifera petal extracts on lipase, adipogenesis, adipolysis, and central receptors of obesity. *Evid. Based Complement. Alternat. Med.* 2013:145925. 10.1155/2013/145925 24348689 PMC3848046

[B356] Verty A. N. Stefanidis A. Mcainch A. J. Hryciw D. H. Oldfield B. (2015). Anti-obesity effect of the CB2 receptor agonist JWH-015 in diet-induced obese mice. *PLoS One* 10:e0140592. 10.1371/journal.pone.0140592 26588700 PMC4654496

[B357] Volkow N. D. Baler R. D. Compton W. M. Weiss S. R. (2014). Adverse health effects of marijuana use. *N. Engl. J. Med.* 370 2219–2227. 24897085 10.1056/NEJMra1402309 PMC4827335

[B358] Vučković S. Srebro D. Vujović K. S. Vučetić Č Prostran M. (2018). Cannabinoids and pain: new insights from old molecules. *Front. Pharmacol.* 9:1259. 10.3389/fphar.2018.01259 30542280 PMC6277878

[B359] Wade M. R. Degroot A. Nomikos G. G. (2006). Cannabinoid CB1 receptor antagonism modulates plasma corticosterone in rodents. *Eur. J. Pharmacol.* 551 162–167. 17030030 10.1016/j.ejphar.2006.08.083

[B360] Wajner M. (2019). Neurological manifestations of organic acidurias. *Nat. Rev. Neurol.* 15 253–271. 30914790 10.1038/s41582-019-0161-9

[B361] Walsh S. Mnich K. Mackie K. Gorman A. M. Finn D. P. Dowd E. (2010). Loss of cannabinoid CB1 receptor expression in the 6-hydroxydopamine-induced nigrostriatal terminal lesion model of Parkinson’s disease in the rat. *Brain Res. Bull.* 81 543–548. 10.1016/j.brainresbull.2010.01.009 20097273 PMC3659808

[B362] Wang Y. Yu T. Zhou Y. Wang S. Zhou X. Wang L. (2020). Carnitine palmitoyltransferase 1C contributes to progressive cellular senescence. *Aging* 12 6733–6755. 10.18632/aging.103033 32289751 PMC7202531

[B363] Warburton D. E. R. Nicol C. W. Bredin S. S. D. (2006). Health benefits of physical activity: the evidence. *CMAJ* 174 801–809. 16534088 10.1503/cmaj.051351 PMC1402378

[B364] Ware M. A. Jensen D. Barrette A. Vernec A. Derman W. (2018). Cannabis and the health and performance of the elite athlete. *Clin. J. Sport Med.* 28 480–484. 30153174 10.1097/JSM.0000000000000650 PMC6116792

[B365] Wellman M. Abizaid A. (2015). Growth hormone secretagogue receptor dimers: a new pharmacological target. *eNeuro* 2:ENEURO.0053-14.2015. 10.1523/ENEURO.0053-14.2015 26464979 PMC4596092

[B366] Whiting P. F. Wolff R. F. Deshpande S. Di Nisio M. Duffy S. Hernandez A. V. (2015). Cannabinoids for medical use: a systematic review and meta-analysis. *JAMA* 313 2456–2473. 26103030 10.1001/jama.2015.6358

[B367] Widmer R. J. Flammer A. J. Lerman L. O. Lerman A. (2015). The Mediterranean diet, its components, and cardiovascular disease. *Am. J. Med.* 128 229–238. 25447615 10.1016/j.amjmed.2014.10.014 PMC4339461

[B368] Wirdefeldt K. Adami H. O. Cole P. Trichopoulos D. Mandel J. (2011). Epidemiology and etiology of Parkinson’s disease: a review of the evidence. *Eur. J. Epidemiol.* 26 Suppl. 1 S1–S58. 21626386 10.1007/s10654-011-9581-6

[B369] Wolfgang M. J. Kurama T. Dai Y. Suwa A. Asaumi M. Matsumoto S. (2006). The brain-specific carnitine palmitoyltransferase-1c regulates energy homeostasis. *Proc. Natl. Acad. Sci. U.S.A.* 103 7282–7287. 16651524 10.1073/pnas.0602205103 PMC1564279

[B370] Woodyard C. (2011). Exploring the therapeutic effects of yoga and its ability to increase quality of life. *Int. J. Yoga* 4 49–54. 10.4103/0973-6131.85485 22022122 PMC3193654

[B371] World Health Organization (2015). *World Report on Ageing and Health.* Geneva: World Health Organization.

[B372] Wrann C. D. White J. P. Salogiannnis J. Laznik-Bogoslavski D. Wu J. Ma D. (2013). Exercise induces hippocampal BDNF through a PGC-1α/FNDC5 pathway. *Cell Metab.* 18 649–659. 24120943 10.1016/j.cmet.2013.09.008 PMC3980968

[B373] Xapelli S. Agasse F. Grade S. Bernardino L. Ribeiro F. F. Schitine C. S. (2014). Modulation of subventricular zone oligodendrogenesis: a role for hemopressin? *Front. Cell. Neurosci.* 8:59. 10.3389/fncel.2014.00059 24578683 PMC3936357

[B374] Xapelli S. Agasse F. Sarda-Arroyo L. Bernardino L. Santos T. Ribeiro F. F. (2013). Activation of type 1 cannabinoid receptor (CB1R) promotes neurogenesis in murine subventricular zone cell cultures. *PLoS One* 8:e63529. 10.1371/journal.pone.0063529 23704915 PMC3660454

[B375] Ye Y. Abu El Haija M. Morgan D. A. Guo D. Song Y. Frank A. (2020). Endocannabinoid receptor-1 and sympathetic nervous system mediate the beneficial metabolic effects of gastric bypass. *Cell Rep.* 33:108270. 10.1016/j.celrep.2020.108270 33113371 PMC7660289

[B376] Zhang X. Zong B. Zhao W. Li L. (2021). Effects of mind-body exercise on brain structure and function: a systematic review on MRI studies. *Brain Sci.* 11:205. 10.3390/brainsci11020205 PMC7915202 33562412

[B377] Zhang Z. Guo Y. Zhang S. Zhang Y. Wang Y. Ni W. (2013). Curcumin modulates cannabinoid receptors in liver fibrosis in vivo and inhibits extracellular matrix expression in hepatic stellate cells by suppressing cannabinoid receptor type-1 in vitro. *Eur. J. Pharmacol.* 721 133–140. 10.1016/j.ejphar.2013.09.042 24076327

[B378] Zhao J. Wang M. Liu W. Ma Z. Wu J. (2020). Activation of cannabinoid receptor 2 protects rat hippocampal neurons against Aβ-induced neuronal toxicity. *Neurosci. Lett.* 735:135207. 10.1016/j.neulet.2020.135207 32592731

[B379] Zolotov Y. Gruber S. A. (2021). Cannabis and aging: research remains in its infancy. *Am. J. Drug Alcohol Abuse* 47 523–526. 10.1080/00952990.2021.1949334 34376078

[B380] Zuardi A. W. Crippa J. A. Hallak J. E. Pinto J. P. Chagas M. H. Rodrigues G. G. (2009). Cannabidiol for the treatment of psychosis in Parkinson’s disease. *J. Psychopharmacol.* 23 979–983. 18801821 10.1177/0269881108096519

